# Supplementary material for: Fe-catalyzed Decarbonylative Alkylative Spirocyclization of N-Arylcinnamamides: Access to Alkylated 1-Azaspirocyclohexadienones
Source: Molecules. 2020 Jan 21;25(3):432. doi: 10.3390/molecules25030432 (PMC7037460; doi:10.3390/molecules25030432)
Supplement: Supplementary file 1 [file molecules-25-00432-s001.pdf]

Supplementary Materials

Fe-catalyzed Decarbonylative Alkylative Spirocyclization of *N*-Arylcinnamamides: Access to Alkylated 1-Azaspirocyclohexadienones

Xiang Peng‡, Ren-Xiang Liu‡, Xiang-Yan Xiao and Luo Yang*

Table of contents

I. General information 1

II. General experimental procedures 1

III. Spectra data of products **3a-3l**, **4b-4k**, **6**, **5** 1

IV. References 12

V. Copies of ^{1}H and ^{13}C NMR spectra of products **3a-3l**, **4b-4k, 6, 5** 12

I. General information

Unless otherwise noted, all commercially available compounds were used as provided without further purification. Dry solvents (toluene, ethyl acetate, dichloroethane, acetonitrile, chlorobenzene, fluorobenzene) were used as commercially available. Thin-layer chromatography (TLC) was performed using E. Merck silica gel 60 F254 precoated plates (0.25 mm) or Sorbent Silica Gel 60 F254 plates. The developed chromatography was analyzed by UV lamp (254 nm). Unless other noted, High-resolution mass spectra (HRMS) were obtained from a JEOL JMS-700 instrument (ESI). Melting points are uncorrected. Nuclear magnetic resonance (NMR) spectra were recorded on a Bruker Avance 400 spectrometer at ambient temperature. Chemical shifts for ^{1}H NMR spectra are reported in parts per million (ppm) from tetramethylsilane with the solvent resonance as the internal standard (chloroform: δ 7.26 ppm). Chemical shifts for ^{13}C NMR spectra are reported in parts per million (ppm) from tetramethylsilane with the solvent as the internal standard ($CDCl_3$: δ 77.16 ppm). Data are reported as following: chemical shift, multiplicity (s = singlet, d = doublet, dd = doublet of doublets, t = triplet, q = quartet, m = multiplet, br = broad signal), coupling constant (Hz), and integration.

II. General experimental procedures

An oven-dried microwave reaction vessel was charged with $FeCl_2$ (2.5 mol%) in EA (0.5 mL, pre-prepared solution), *N*-(4-hydroxyphenyl)-*N*-methylcinnamamide (**1a,** 0.1 mmol , 1.0 equiv), isobutyraldehyde (**2a**, 0.5 mmol, 5.0 equiv) and DTBP (0.3 mmol, 3.0 equiv). The vessel was sealed and heated at 122°C (oil bath temperature) for 24 h. Afterwards the resulting mixture was cooled to room temperature, the solvent was removed in vacuo. The residue was purified by column chromatography on silica gel with a mixture of ethyl acetate/petroleum ether (1:3) as eluent to give products **3a**.

III. Spectra data of products 3a-3l, 4b-4k, 6, 5

(3a) 3-isopropyl-1-methyl-4-phenyl-1-azaspiro[4.5]deca-6,9-diene-2,8-dione

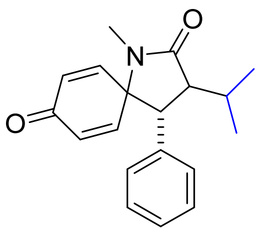

The title compound was prepared according to the general procedure described above by the reaction between *N*-(4-hydroxyphenyl)-*N*-methylcinnamamide (**1a**) with isobutyraldehyde (**2a**), and purified by flash column chromatography as yellow oil (19.8 mg, 67%).

^{1}H NMR (400 MHz, $CDCl_3$) δ 7.27 – 7.24 (m, 3H), 7.10 (dd, *J* = 7.6, 2.4 Hz, 2H), 6.78 (dd, *J* = 10.0, 3.2 Hz, 1H), 6.55 (dd, *J* = 10.2, 3.0 Hz, 1H), 6.39 (dd, *J* = 10.2, 2.0 Hz, 1H), 6.00 (dd, *J* = 10.2, 2.0 Hz, 1H), 3.43 (d, *J* = 12.0 Hz, 1H), 3.14 (dd, *J* = 11.8, 3.6 Hz, 1H), 2.73 (s, 3H), 2.38 – 2.34 (m, 1H), 1.01 (d, *J* = 6.8 Hz, 3H), 0.83 (d, *J* = 7.2 Hz, 3H). ^{13}C NMR (100 MHz, $CDCl_3$) δ 184.46, 175.22, 149.40, 147.05, 135.04, 132.33, 131.51, 128.63, 128.21, 64.95, 50.84, 49.05, 28.07, 27.14, 20.14, 18.78. IR (cm^{-1}): 3032, 2965, 2932, 2875, 1672, 1630, 1606, 1498, 1446, 1454, 1418, 1393, 1374, 1260, 1141, 1119, 1065, 991, 865, 794, 724, 700. HRMS: calcd. for $C_{19}H_{21}NO_2$ Na^+ $[M+Na]^+$: 318.1465; Found: 318.1442.

(3b) 1-methyl-3-(pentan-3-yl)-4-phenyl-1-azaspiro[4.5]deca-6,9-diene-2,8-dione

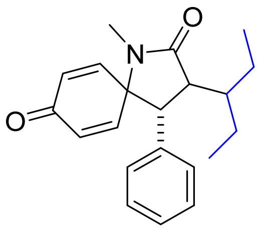

The title compound was prepared according to the general procedure described above by the reaction between *N*-(4-hydroxyphenyl)-*N*-methylcinnamamide (**1a**) with 2-ethylbutanal (**2b**), and purified by flash column chromatography as yellow oil (23.0 mg, 71%).

^{1}H NMR (400 MHz, $CDCl_3$) δ 7.26 – 7.24 (m, 3H), 7.09 (dd, *J* = 7.6, 2.8 Hz, 2H), 6.77 (dd, *J* = 10.0, 3.2 Hz, 1H), 6.60 (dd, *J* = 10.4, 3.2 Hz, 1H), 6.37 (dd, *J* = 10.0, 2.0 Hz, 1H), 6.00 (dd, *J* = 10.0, 2.0 Hz, 1H), 3.45 (d, *J* = 11.8 Hz, 1H), 3.31 (dd, *J* = 11.8, 2.6 Hz, 1H), 2.74 (s, 3H), 1.78 – 1.74 (m, 2H), 1.53 – 1.46 (m, 1H), 1.39 – 1.32 (m, 2H), 0.96 (t, *J* = 7.4 Hz, 3H), 0.80 (t, *J* = 7.3 Hz, 3H). ^{13}C NMR (100 MHz, $CDCl_3$) δ 184.46, 175.15, 149.45, 146.94, 134.72, 132.36, 131.58, 128.68, 128.59, 128.28, 65.02, 51.48, 48.30, 34.68, 27.12, 26.47, 16.47, 12.44. IR (cm^{-1}): 3032, 2961, 2931, 2875, 1692, 1672, 1630, 1454, 1419, 1392, 1376, 1260, 1173, 1141, 1119, 1066, 992, 865, 723,700, 662, 563. HRMS: calcd. for $C_{21}H_{25}NO_2$ Na^+ $[M+Na]^+$: 346.1778; Found: 346.1753 .

(3c) 3-(sec-butyl)-1-methyl-4-phenyl-1-azaspiro[4.5]deca-6,9-diene-2,8-dione

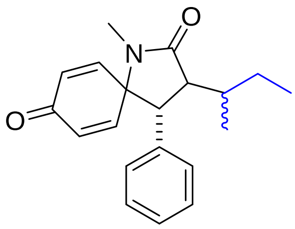

The title compound was prepared according to the general procedure described above by the reaction between *N*-(4-hydroxyphenyl)-*N*-methylcinnamamide (**1a**) with 2-methylbutanal (**2c**), and purified by flash column chromatography as yellow oil (21.0 mg, 68%).

^{1}H NMR (400 MHz, $CDCl_3$) δ 7.27 – 7.24 (m, 3H), 7.09 (d, *J* = 9.6, 2.4 Hz, 2H), 6.78 (dd, *J* = 10.0, 2.8 Hz, 1H), 6.54 (dd, *J* = 10.0, 3.2 Hz, 1H), 6.39 (dd, *J* = 10.4, 2.0 Hz, 1H), 6.00 (dd, *J* = 10.0, 2.0 Hz, 1H), 3.45 (d, *J* = 12.0 Hz, 1H), 3.20 (dd, *J* = 12.0, 2.8 Hz, 1H), 2.73 (s, 3H), 1.93 – 1.85 (m, 1H), 1.60 – 1.56 (m, 1H), 1.45 – 1.37 (m, 1H), 0.93 (t, *J* = 7.4 Hz, 3H), 0.85 (d, *J* = 7.0 Hz, 3H). ^{13}C NMR (100 MHz, $CDCl_3$) δ 184.46, 175.15, 149.45, 146.94, 134.72, 132.36, 131.58, 128.68, 128.59, 128.28, 65.02, 51.48, 48.30, 34.68, 27.12, 26.47, 16.47, 12.44. IR (cm^{-1}): 3059, 3032, 2962, 2932, 2875, 1672, 1630, 1498, 1454, 1419, 1392, 1375, 1260, 1172, 1143, 1065, 990, 865, 767, 700, 621. HRMS: calcd. for $C_{20}H_{23}NO_2$ Na^+ $[M+Na]^+$: 332.1621; Found: 332.1605.

(3d) 1-methyl-3-(pentan-2-yl)-4-phenyl-1-azaspiro[4.5]deca-6,9-diene-2,8-dione

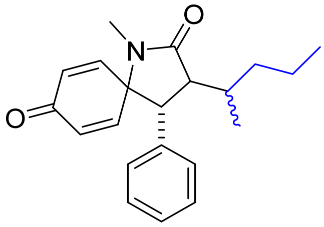

The title compound was prepared according to the general procedure described above by the reaction between *N*-(4-hydroxyphenyl)-*N*-methylcinnamamide (**1a**) with 2-methylpentanal (**2d**), and purified by flash column chromatography as yellow oil (22.3 mg, 69%).

^{1}H NMR (400 MHz, $CDCl_3$) δ 7.26 – 7.23 (m, 3H), 7.09 (dd, *J* = 7.6, 2.4 Hz, 2H), 6.76 (dd, *J* = 10.0, 2.8 Hz, 1H), 6.57 (dd, *J* = 10.0, 2.8 Hz, 1H), 6.38 (dd, *J* = 10.0, 2.0 Hz, 1H), 6.00 (dd, *J* = 10.0, 2.0 Hz, 1H), 3.44 (d, *J* = 10.8 Hz, 1H), 3.21 (dd, *J* = 11.6, 3.2 Hz, 1H), 2.74 (s, 3H), 2.30 – 2.23 (m, 1H), 1.37 – 1.23 (m, 2H), 1.16 – 1.01 (m, 2H), 0.96 (d, *J* = 6.9 Hz, 3H), 0.70 (t, *J* = 7.4 Hz, 3H). ^{13}C NMR (100 MHz, $CDCl_3$) δ 184.48, 175.64, 149.44, 147.26, 146.96, 135.09, 132.34, 132.24, 131.58, 131.47, 128.66, 128.58, 128.33, 128.29, 128.23, 65.05, 50.43, 48.48, 48.25, 36.50, 35.77, 32.70, 27.24, 20.94, 20.67, 15.97, 14.31, 13.98. IR (cm^{-1}): 3059, 3032, 2958, 2928, 2872, 1672, 1630, 1498, 1454, 1421, 1377, 1261, 1172, 1119, 1067, 990, 865, 794, 724, 700, 621. HRMS: calcd. for $C_{21}H_{25}NO_2$ Na^+ $[M+Na]^+$: 346.1778; Found: 346.1752.

(3e) 3-(heptan-3-yl)-1-methyl-4-phenyl-1-azaspiro[4.5]deca-6,9-diene-2,8-dione[1]

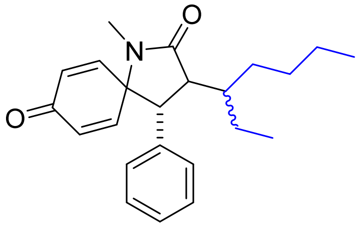

The title compound was prepared according to the general procedure described above by the reaction between *N*-(4-hydroxyphenyl)-*N*-methylcinnamamide (**1a**) with 2-ethylhexanal (**2e**), and purified by flash column chromatography as yellow oil (21.4 mg, 61%).

^{1}H NMR (400 MHz, $CDCl_3$) δ 7.25 – 7.22 (m, 3H), 7.09 (dd, *J* = 7.6, 2.4 Hz, 2H), 6.77 (dt, *J* = 10.0, 2.8 Hz, 1H), 6.60 (dt, *J* = 10.2, 3.2 Hz, 1H), 6.37 (dd, *J* = 10.0, 2.0 Hz, 1H), 6.00 (dt, *J* = 10.2, 2.2

Hz, 1H), 3.44 (dd, J = 11.8, 3.2 Hz, 1H), 3.30 (dd, J = 12.0, 3.0 Hz, 1H), 2.74 (s, 3H), 1.84 – 1.79 (m, 1H), 1.53 – 1.37 (m, 1H), 1.37 – 1.26 (m, 4H), 1.23 – 1.11 (m, 3H), 0.95 (t, J = 8.6 Hz, 3H), 0.81 – 0.72 (m, 3H). ^{13}C NMR (100 MHz, $CDCl_3$) δ 184.48, 175.79, 149.53, 147.23, 134.93, 132.23, 131.54, 128.61, 128.44, 128.29, 65.08, 51.27, 46.04, 45.96, 40.21, 40.10, 31.12, 30.60, 30.31, 30.01, 27.22, 24.47, 24.24, 23.10, 22.80, 14.24, 14.02, 12.69, 12.24. IR (cm^{-1}): 3060, 3032, 2958, 2929, 2872, 1691, 1672, 1630, 1499, 1455, 1393, 1376, 1259, 1173, 1119, 1066, 991, 865, 766,722, 700, 662.

(3f) 3-(1-(4-isopropylphenyl)propan-2-yl)-1-methyl-4-phenyl-1-azaspiro[4.5]deca-6,9-diene-2,8-dione

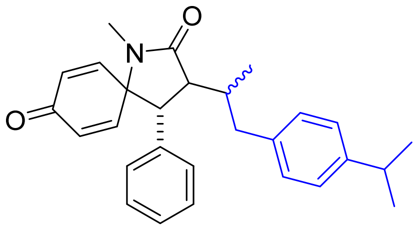

The title compound was prepared according to the general procedure described above by the reaction between *N*-(4-hydroxyphenyl)-*N*-methylcinnamamide (**1a**) with 3-(4-isopropylphenyl)-2-methylpropanal (**2f**), and purified by flash column chromatography as yellow oil (30.6 mg, 74%).

^{1}H NMR (400 MHz, $CDCl_3$) δ 7.24 – 7.23 (m, 1H), 7.17 – 7.13 (m, 2.5H), 7.20 – 7.07 (m, 2.5H), 7.00 (dd, J = 6.0, 2.0 Hz, 1H), 6.89 (dd, J = 6.0, 1.6 Hz, 1H), 6.80 – 6.73 (m, 1.5H), 6.65 (dd, J = 10.0, 3.2 Hz, 0.5H), 6.53 (dd, J = 11.4, 3.2 Hz, 0.5H), 6.43 – 6.32 (m, 1.5H), 5.96 (td, J = 10.1, 2.0 Hz, 1H), 3.44 – 3.38 (m, 1H), 3.22 – 3.14 (m, 1H), 3.08 – 3.02 (m, 1H), 2.91 – 2.84 (m, 1H), 2.72 (d, J = 8 Hz, 3H), 2.67 (t, J = 6.8 Hz, 0.5H), 2.49 (q, J = 6.8 Hz, 0.5H), 2.28 (d, 5.6 Hz, 0.5H), 2.06 – 2.00(m 1H), 1.28 – 1.21 (m, 6H), 0.99 (dd, J = 6.8, 4.4 Hz, 3H). ^{13}C NMR (100 MHz, $CDCl_3$) δ 174.77, 149.42, 149.36, 147.09, 146.84, 146.76, 146.58, 137.97, 137.48, 134.68, 133.85, 132.44, 132.18, 131.53, 131.45, 129.49, 129.06, 128.61, 128.58, 128.54, 128.30, 128.20, 128.00, 126.41, 126.33, 64.95, 64.89, 51.61, 50.78, 47.81, 45.16, 40.13, 40.02, 34.93, 34.56, 33.86, 33.79, 27.19, 26.98, 24.33, 24.19, 16.74, 15.80. IR (cm^{-1}): 3049, 3030, 2960, 2928, 2873, 1689, 1631, 1499, 1454, 1392, 1378, 1265, 1172, 1114, 1058, 990, 864, 735, 700, 570. HRMS: calcd. for $C_{28}H_{31}NO_2Na^+$ $[M+Na]^+$: 436.2247; Found: 436.2217.

(3g) 3-cyclohexyl-1-methyl-4-phenyl-1-azaspiro[4.5]deca-6,9-diene-2,8-dione[1]

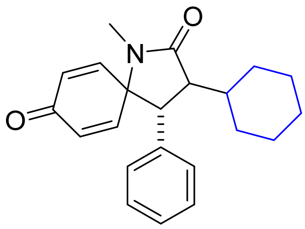

The title compound was prepared according to the general procedure described above by the reaction between *N*-(4-hydroxyphenyl)-*N*-methylcinnamamide (**1a**) with cyclohexanecarbaldehyde (**2g**), and purified by flash column chromatography as yellow oil (24.1 mg, 72%).

^{1}H NMR (400 MHz, $CDCl_3$) δ 7.27 – 7.24(m, 3H), 7.09 (dd, J = 7.6, 2.4 Hz, 2H), 6.76 (dd, J = 10.0, 3.2 Hz, 1H), 6.54 (dd, J = 10.2, 3.0 Hz, 1H), 6.39 (dd, J = 10.4, 2.0 Hz, 1H), 5.98 (dd, J = 10.2, 2.0

Hz, 1H), 3.48 (d, J = 12.0 Hz, 1H), 3.11 (dd, J = 11.8, 3.6 Hz, 1H), 2.73 (s, 3H), 2.01 – 1.94 (m, 1H), 1.73 (d, J = 13.2, 1H), 1.63 – 1.56 (m, 3H), 1.38 – 1.13 (m, 4H), 1.08 – 0.99 (m, 1H), 0.87 – 0.81 (m, 1H). ^{13}C NMR (100 MHz, $CDCl_3$) δ 184.50, 175.30, 149.45, 147.09, 135.06, 132.33, 131.45, 128.63, 128.22, 128.19, 65.00, 51.02, 48.81, 38.27, 30.98, 29.14, 27.19, 26.71, 26.56, 26.23. IR (cm^{-1}): 3057, 3032, 2925, 2852, 1690, 1672, 1499, 1450, 1419, 1393, 1377, 1260, 1172, 1134, 1096, 1069, 991, 864, 796, 732, 657, 569.

(3h) 3-cyclopentyl-1-methyl-4-phenyl-1-azaspiro[4.5]deca-6,9-diene-2,8-dione[1]

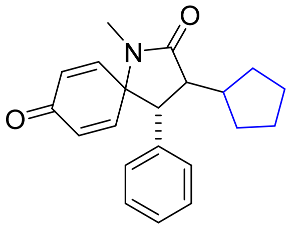

The title compound was prepared according to the general procedure described above by the reaction between *N*-(4-hydroxyphenyl)-*N*-methylcinnamamide (**1a**) with cyclopentanecarbaldehyde (**2h**), and purified by flash column chromatography as yellow oil (19.6 mg, 61%).

^{1}H NMR (400 MHz, $CDCl_3$) δ 7.27 – 7.24 (m, 3H), 7.09 (dd, J = 7.6, 2.4 Hz, 2H), 6.77 (dd, J = 10.2, 3.0 Hz, 1H), 6.54 (dd, J = 10.2, 3.1 Hz, 1H), 6.39 (dd, J = 10.2, 2.0 Hz, 1H), 5.99 (dd, J = 10.2, 2.0 Hz, 1H), 3.38 (d, J = 11.6 Hz, 1H), 3.21 (dd, J = 11.8, 6.2 Hz, 1H), 2.73 (s, 3H), 2.24 – 2.18 (m, 1H), 2.05 – 1.96 (m, 1H), 1.87 – 1.76 (m, 3H), 1.50 – 1.38 (m, 3H), 1.25 – 1.21 (m, 1H). ^{13}C NMR (100 MHz, $CDCl_3$) δ 184.50, 175.30, 149.45, 147.09, 135.06, 132.33, 131.45, 128.63, 128.22, 128.19, 65.06, 53.45, 46.83, 41.20, 29.85, 29.56, 27.16, 25.18, 24.99. IR (cm^{-1}): 3059, 3031, 2923, 2869, 1730, 1692, 1671, 1630, 1453, 1442, 1393, 1375, 1260, 1172, 1075, 991, 865, 794, 732,700, 645, 569.

(3i) 3-(tert-butyl)-1-methyl-4-phenyl-1-azaspiro[4.5]deca-6,9-diene-2,8-dione

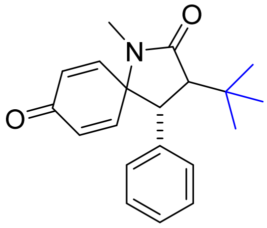

The title compound was prepared according to the general procedure described above by the reaction between *N*-(4-hydroxyphenyl)-*N*-methylcinnamamide (**1a**) with pivalaldehyde (**2i**), and purified by flash column chromatography as yellow oil (16.7 mg, 52%).

^{1}H NMR (400 MHz, $CDCl_3$) δ 7.27 (s, 3H), 7.21 (s, 2H), 6.77 (dd, J = 10.0, 3.2 Hz, 1H), 6.46 (dd, J = 10.2, 3.2 Hz, 1H), 6.38 (dd, J = 10.0, 2.0 Hz, 1H), 5.92 (dd, J = 10.2, 2.0 Hz, 1H), 3.40 (d, J = 11.2 Hz, 1H), 3.01 (d, J = 11.6 Hz, 1H), 2.70 (s, 3H), 1.00 (s, 9H). ^{13}C NMR (100 MHz, $CDCl_3$) δ 184.48 , 175.09 , 149.54 , 147.98 , 136.60 , 132.11 , 131.04 , 128.02 , 64.35 , 52.50 , 51.33 , 33.87 , 28.08 , 27.22 . IR (cm^{-1}): 3031, 2958, 2870, 1688, 1672, 1630, 1605, 1468, 1420, 1392, 1370, 1260, 1244, 1171, 1119, 1093, 864, 794, 720, 735,700, 610, 563. HRMS: calcd. for $C_{20}H_{23}NO_2$ Na^+ $[M+Na]^+$: 332.1621; Found: 332.1597.

(3j) 3-isobutyl-1-methyl-4-phenyl-1-azaspiro[4.5]deca-6,9-diene-2,8-dione

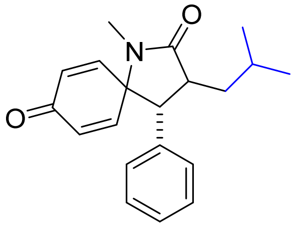

The title compound was prepared according to the general procedure described above by the reaction between *N*-(4-hydroxyphenyl)-*N*-methylcinnamamide (**1a**) with 3-methylbutanal (**2j**), and purified by flash column chromatography as yellow oil (19.8 mg, 64%).

^{1}H NMR (400 MHz, $CDCl_3$) δ 7.27 – 7.24 (m, 3H), 7.09 (dd, *J* = 7.6, 2.4 Hz, 2H), 6.76 (dd, *J* = 10.0, 3.2 Hz, 1H), 6.59 (dd, *J* = 10.4, 3.0 Hz, 1H), 6.39 (dd, *J* = 10.0, 2.0 Hz, 1H), 6.01 (dd, *J* = 10.2, 2.0 Hz, 1H), 3.25 (d, *J* = 11.6 Hz, 1H), 3.15 – 3.09 (m, 1H), 2.74 (s, 3H), 1.89 – 1.83 (m, 1H), 1.74 – 1.67 (m, 1H), 1.38 – 1.31 (m, 1H), 0.86 (d, *J* = 7.2 Hz, 3H), 0.80 (d, *J* = 6.4 Hz, 3H). ^{13}C NMR (100 MHz, $CDCl_3$) δ 184.45, 176.38, 149.36, 146.81, 134.34, 132.40, 131.59, 128.66, 128.36, 128.25, 65.23, 56.35, 41.66, 41.10, 27.23, 25.20, 22.93, 22.37. IR (cm^{-1}): 3056, 3033, 2956, 2927, 2869, 1692, 1672, 1630, 1467, 1454, 1393, 1376, 1262, 1172, 1137, 1080, 1060, 866, 790, 721, 700. HRMS: calcd. for $C_{20}H_{23}NO_2$ Na^+ $[M+Na]^+$: 332.1621; Found: 332.1597.

(3k) 3-ethyl-1-methyl-4-phenyl-1-azaspiro[4.5]deca-6,9-diene-2,8-dione

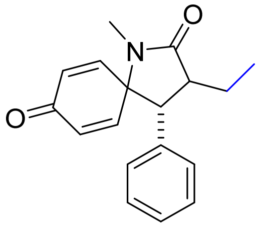

The title compound was prepared according to the general procedure described above by the reaction between *N*-(4-hydroxyphenyl)-*N*-methylcinnamamide (**1a**) with propionaldehyde (**2k**), and purified by flash column chromatography as yellow oil (16.1 mg, 56%).

^{1}H NMR (400 MHz, $CDCl_3$) δ 7.27 – 7.24 (m, 3H), 7.10 (dd, *J* = 8.0, 2.4 Hz 2H), 6.79 (dd, *J* = 10.0, 3.2 Hz, 1H), 6.57 (dd, *J* = 10.2, 3.2 Hz, 1H), 6.41 (dd, *J* = 10.0, 2.0 Hz, 1H), 6.02 (dd, *J* = 10.2, 2.0 Hz, 1H), 3.34 (d, *J* = 12.0 Hz, 1H), 3.13 – 3.06 (m, 1H), 2.75 (s, 3H), 1.96 – 1.86 (m, 1H), 1.77 – 1.68 (m, 1H), 0.89 (t, *J* = 7.4 Hz, 3H). ^{13}C NMR (100 MHz, $CDCl_3$) δ 184.46, 175.72, 149.32, 146.57, 134.25, 132.53, 131.70, 128.70, 128.34, 128.17, 65.14, 54.39, 44.64, 27.17, 23.01, 11.18 . IR (cm^{-1}): 3056, 3032, 2965, 2931, 2877, 1692, 1672, 1629, 1454, 1420, 1394, 1376, 1262, 1174, 1125, 1060, 988, 865, 719, 699, 659. HRMS: calcd. for $C_{18}H_{19}NO_2$ Na^+ $[M+Na]^+$: 304.1308; Found: 304.1286.

(3l) 3-benzyl-1-methyl-4-phenyl-1-azaspiro[4.5]deca-6,9-diene-2,8-dione

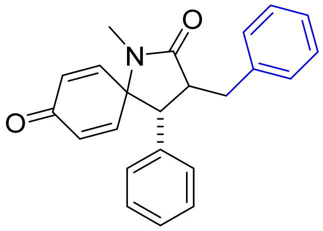

The title compound was prepared according to the general procedure described above by the reaction between *N*-(4-hydroxyphenyl)-*N*-methylcinnamamide (**1a**) with 2-phenylacetaldehyde (**2l**), and purified by flash column chromatography as yellow oil (14.4 mg, 42%).

^{1}H NMR (400 MHz, $CDCl_3$) δ 7.24 – 7.16 (m, 6H), 7.07 – 7.04 (m, 2H), 6.99 – 6.97 (m, 2H), 6.56 (dd, *J* = 10.2, 3.0 Hz, 1H), 6.47 (dd, *J* = 10.0, 3.0 Hz, 1H), 6.30 (dd, *J* = 10.2, 2.0 Hz, 1H), 6.00 (dd, *J* = 10.2, 2.0 Hz, 1H), 3.42 (dt, *J* = 12.2, 5.4 Hz, 1H), 3.30 – 3.20 (m, 2H), 2.93 (dd, *J* = 13.6, 5.8 Hz, 1H), 2.71 (s, 3H). ^{13}C NMR (100 MHz, $CDCl_3$) δ 184.41, 174.77, 149.24, 146.36, 137.36, 133.43, 132.39, 131.76, 129.98, 128.69, 128.47, 128.28, 128.27, 126.71, 65.01, 52.74, 45.16, 34.06, 27.23. IR (cm^{-1}): 3060, 3029, 2922, 2853, 1693, 1672, 1630, 1496, 1453, 1393, 1377, 1261, 1172, 1130, 1092, 1075, 865, 788, 721, 699. HRMS: calcd. for $C_{23}H_{21}NO_2$ Na^+ $[M+Na]^+$: 366.1465; Found: 366.1443.

(4b) 3-isopropyl-1-methyl-4-phenyl-1-azaspiro[4.5]deca-6,9-diene-2,8-dione

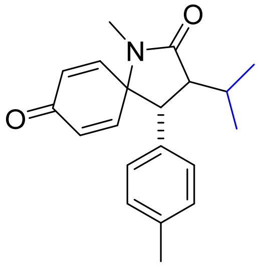

The title compound was prepared according to the general procedure described above by the reaction between *N*-(4-hydroxyphenyl)-*N*-methyl-3-(p-tolyl)acrylamide (**1b**) with isobutyraldehyde (**2a**), and purified by flash column chromatography as yellow oil (22.2 mg, 72%).

^{1}H NMR (400 MHz, $CDCl_3$) δ 7.05 (d, *J* = 7.6 Hz, 2H), 6.53 (d, *J* = 7.6 Hz, 2H), 6.77 (dd, *J* = 10.0, 3.2 Hz, 1H), 6.56 (dd, *J* = 10.2, 3.0 Hz, 1H), 6.38 (dd, *J* = 10.0, 2.0 Hz, 1H), 6.01 (dd, *J* = 10.2, 1.8 Hz, 1H), 3.40 (d, *J* = 11.6 Hz, 1H), 3.11 (dd, *J* = 12.0, 3.6 Hz, 1H), 2.73 (s, 3H), 2.36 – 2.31 (m, 1H), 2.28 (s, 3H), 1.01 (d, *J* = 6.8 Hz, 3H), 0.83 (d, *J* = 7.2 Hz, 3H). ^{13}C NMR (100 MHz, $CDCl_3$) δ 184.60, 175.34, 149.56, 147.20, 137.93, 132.28, 131.95, 131.50, 129.33, 128.09, 65.05, 50.58, 49.13, 28.07, 27.14, 21.16, 20.13, 18.83. IR (cm^{-1}): 3046, 3030, 2960, 2929, 2876, 1690, 1672, 1629, 1516, 1465, 1419, 1393, 1375, 1263, 1065, 992, 864, 736, 639, 575. HRMS: calcd. for $C_{20}H_{23}NO_2$ Na^+ $[M+Na]^+$: 332.1621; Found: 332.1602.

(4c) 4-(4-chlorophenyl)-3-isopropyl-1-methyl-1-azaspiro[4.5]deca-6,9-diene-2,8-dione

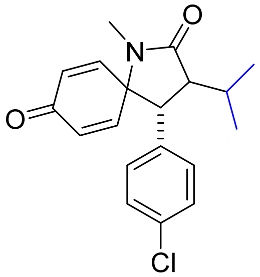

The title compound was prepared according to the general procedure described above by the reaction between 3-(4-chlorophenyl)-*N*-(4-hydroxyphenyl)-N-methylacrylamide (**1c**) with isobutyraldehyde (**2a**), and purified by flash column chromatography as yellow oil (22.7 mg, 69%).

^{1}H NMR (400 MHz, $CDCl_3$) δ 7.24 (dd, *J* = 6.4, 2.0 Hz, 2H), 7.05 (dd *J* = 6.4, 2.0 Hz, 2H), 6.76 (dd, *J* = 10.2, 3.0 Hz, 1H), 6.54 (dd, *J* = 10.2, 3.2 Hz, 1H), 6.40 (dd, *J* = 10.0, 2.0 Hz, 1H), 6.05 (dd, *J* = 10.2, 2.0 Hz, 1H), 3.40 (d, *J* = 12.0 Hz, 1H), 3.08 (dd, *J* = 12.0, 3.6 Hz, 1H), 2.73 (s, 3H), 2.38 – 2.30 (m, 1H), 1.00 (d, *J* = 6.8 Hz, 3H), 0.82 (d, *J* = 6.8 Hz, 3H). ^{13}C NMR (100 MHz, $CDCl_3$) δ 184.23, 174.90, 149.09, 146.66, 134.10, 133.70, 132.53, 131.86, 129.53, 128.91, 64.83, 50.23, 49.23, 28.06, 27.19, 20.29, 18.68. IR (cm^{-1}): 3050, 2961, 2931, 2874, 1692, 1672, 1630, 1494, 1466, 1417, 1392, 1373, 1259, 1173, 1121, 1092, 1014, 866, 830, 736. HRMS: calcd. for $C_{19}H_{20}ClNO_2$ Na^+ $[M+Na]^+$: 352.1075; Found: 352.1059.

(4d) 4-(3-chlorophenyl)-3-isopropyl-1-methyl-1-azaspiro[4.5]deca-6,9-diene-2,8-dione

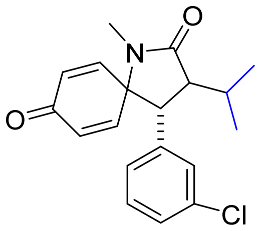

The title compound was prepared according to the general procedure described above by the reaction between 3-(3-chlorophenyl)-*N*-(4-hydroxyphenyl)-*N*-methylacrylamide (**1d**) with isobutyraldehyde (**2a**), and purified by flash column chromatography as yellow oil (21.1 mg, 64%).

^{1}H NMR (400 MHz, $CDCl_3$) δ 7.25 – 7.20 (m, 2H), 7.10 (s 1H), 7.00 (dt, *J* = 7.0, 1.8 Hz, 1H), 6.76 (dd, *J* = 10.2, 3.2 Hz, 1H), 6.56 (dd, *J* = 10.2, 3.0 Hz, 1H), 6.42 (dd, *J* = 10.0, 2.0 Hz, 1H), 6.06 (dd, *J* = 10.2, 2.0 Hz, 1H), 3.40 (d, *J* = 12.0 Hz, 1H), 3.08 (dd, *J* = 12.0, 3.6 Hz, 1H), 2.73 (s, 3H), 2.38 – 2.30 (m, 1H), 1.01 (d, *J* = 6.8 Hz, 3H), 0.83 (d, *J* = 6.8 Hz, 3H). ^{13}C NMR (100 MHz, $CDCl_3$) δ 184.22, 174.78, 148.99, 146.52, 137.35, 134.58, 132.62, 131.87, 129.96, 128.55, 128.35, 126.53, 64.76, 50.52, 49.20, 28.09, 27.17, 20.23, 18.75 . IR (cm^{-1}): 3056, 2961, 2931, 2874, 1691, 1672, 1630, 1468, 1422, 1392, 1375, 1261, 1173, 1119, 1082, 880, 852, 736, 690, 623. HRMS: calcd. for $C_{19}H_{20}ClNO_2$ Na^+ $[M+Na]^+$: 352.1075; Found: 352.1053.

(4e) 4-(2-chlorophenyl)-3-isopopyl-1-methyl-1-azaspiro[4.5]deca-6,9-diene-2,8-dione

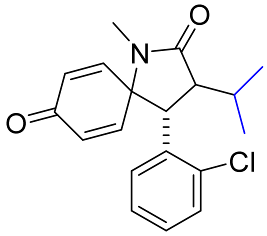

The title compound was prepared according to the general procedure described above by the reaction between 3-(2-chlorophenyl)-*N*-(4-hydroxyphenyl)-*N*-methylacrylamide (**1e**) with isobutyraldehyde (**2a**), and purified by flash column chromatography as yellow oil (21.0 mg, 63%).

^{1}H NMR (400 MHz, $CDCl_3$) δ 7.34 – 7.24 (m, 3H), 7.22 – 7.16 (m, 2H), 6.88 (dd, *J* = 10.2, 3.0 Hz, 1H), 6.68 (dd, *J* = 10.2, 3.2 Hz, 1H), 6.33 (dd, *J* = 10.2, 2.0 Hz, 1H), 6.12 (dd, *J* = 10.2, 2.0 Hz, 1H), 4.25 (d, *J* = 11.8 Hz, 1H), 3.04 (dd, *J* = 11.8, 3.6 Hz, 1H), 2.74 (s, 3H), 2.39 – 2.31 (m, 1H), 1.00 (d, *J* = 6.8 Hz, 3H), 0.76 (d, *J* = 7.0 Hz, 3H). ^{13}C NMR (100 MHz, $CDCl_3$) δ 184.41, 174.88, 149.76, 146.58, 133.44, 131.83, 131.81, 130.45, 135.01, 129.37, 129.22, 126.68, 64.64, 51.18, 46.06, 28.23, 26.94, 20.50, 18.43. IR (cm^{-1}): 3059, 2960, 2931, 2873, 1692, 1672, 1631, 1468, 1422, 1392, 1374,

1260, 1174, 1116, 1065, 1037, 864, 750, 736, 698. HRMS: calcd. for $C_{19}H_{20}ClNO_2$ Na^+ $[M+Na]^+$: 352.1075; Found: 352.1054.

(4f) 4-(4-bromophenyl)-3-isopropyl-1-methyl-1-azaspiro[4.5]deca-6,9-diene-2,8-dione

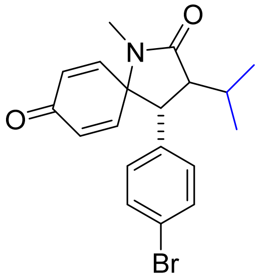

The title compound was prepared according to the general procedure described above by the reaction between 3-(4-bromophenyl)-*N*-(4-hydroxyphenyl)-*N*-methylacrylamide (**1f**) with isobutyraldehyde (**2a**), and purified by flash column chromatography as yellow oil (23.5 mg, 63%).

^{1}H NMR (400 MHz, $CDCl_3$) δ 7.41 – 7.38 (m, 2H), 6.99 (dd, *J* = 6.4, 2.0 Hz, 2H), 6.75 (dd, *J* = 10.0, 3.2 Hz, 1H), 6.53 (dd, *J* = 10.2, 3.0 Hz, 1H), 6.40 (dd, *J* = 10.2, 2.0 Hz, 1H), 6.05 (dd, *J* = 10.2, 2.0 Hz, 1H), 3.39 (d, *J* = 12.0 Hz, 1H), 3.07 (dd, *J* = 12.0, 3.6 Hz, 1H), 2.73 (s, 3H), 2.38 – 2.30 (m, 1H), 0.99 (d, *J* = 7.0 Hz, 3H), 0.82 (d, *J* = 7.0 Hz, 3H). ^{13}C NMR (100 MHz, $CDCl_3$) δ 184.22, 174.83, 149.09, 146.60, 134.26, 132.56, 131.90, 131.88, 129.86, 122.24, 64.73, 50.31, 49.19, 28.09, 27.19, 20.31, 18.70. IR (cm^{-1}): 3048, 2960, 2929, 2873, 1693, 1671, 1630, 1491, 1466, 1392, 1374, 1260, 1173, 1120, 1010, 866, 827, 755, 629, 571. HRMS: calcd. for $C_{19}H_{20}BrNO_2$ $Na^+[M+Na]^+$: 396.0570; Found: 396.0550.

(4g) 3-isopropyl-1-methyl-4-(4-(trifluoromethyl)phenyl)-1-azaspiro[4.5]deca-6,9-diene-2,8-dio-ne

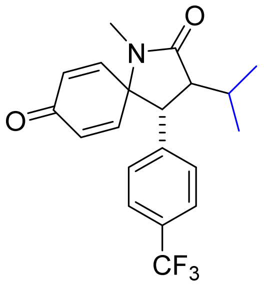

The title compound was prepared according to the general procedure described above by the reaction between *N*-(4-hydroxyphenyl)-*N*-methyl-3-(4-(trifluoromethyl)phenyl)acrylamide (**1g**) with isobutyraldehyde (**2a**), and purified by flash column chromatography as yellow oil (23.6 mg, 65%).

^{1}H NMR (400 MHz, $CDCl_3$) δ 7.54 (d, *J* = 7.8 Hz, 2H), 7.25 (t, *J* = 7.4 Hz, 2H), 6.79 (dd, *J* = 10.2, 3.2 Hz, 1H), 6.55 (dd, *J* = 10.2, 3.2 Hz, 1H), 6.42 (dd, *J* = 10.0, 2.0 Hz, 1H), 6.04 (dd, *J* = 10.2, 2.0 Hz, 1H), 3.49 (d, *J* = 11.8 Hz, 1H), 3.15 (dd, *J* = 12.0, 3.8 Hz, 1H), 2.74 (s, 3H), 2.39 – 2.31 (m, 1H), 1.01 (d, *J* = 7.0 Hz, 3H), 0.82 (d, *J* = 7.0 Hz, 3H). ^{13}C NMR (100 MHz, $CDCl_3$) δ 184.05, 174.67, 148.90, 146.28, 139.43, 132.68, 131.97, 128.69, 125.73, 125.69, 64.68, 50.57, 49.24, 28.13, 27.17, 20.30, 18.72. ^{19}F NMR (376 MHz, CDCl3) δ 62.65 (s, 1F). IR (cm^{-1}): 3050, 2962, 2933, 2876, 1692, 1672, 1631, 1468, 1422, 1392, 1375, 1327, 1166, 1124, 1069, 1017, 869, 853, 737, 602. HRMS: calcd. for $C_{20}H_{20}F_3NO_2$ Na^+ $[M+Na]^+$: 386.1338; Found: 386.1319.

(4h) 1-ethyl-3-isopropyl-4-phenyl-1-azaspiro[4.5]deca-6,9-diene-2,8-dione

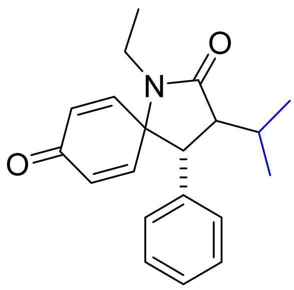

The title compound was prepared according to the general procedure described above by the reaction between *N*-ethyl-*N*-(4-hydroxyphenyl)cinnamamide (**1h**) with isobutyraldehyde (**2a**), and purified by flash column chromatography as yellow oil (22.0 mg, 71%).

^{1}H NMR (400 MHz, $CDCl_3$) δ 7.27 – 7.23 (m, 3H), 7.09 (dd, *J* = 8.0, 2.0 Hz, 2H), 6.82 (dd, *J* = 10.1, 3.1 Hz, 1H), 6.62 (dd, *J* = 10.2, 3.0 Hz, 1H), 6.37 (dd, *J* = 10.1, 2.0 Hz, 1H), 5.95 (dd, *J* = 10.2, 2.0 Hz, 1H), 3.42 (d, *J* = 12.0 Hz, 1H), 3.36 – 3.27 (m, 1H), 3.14 (dd, *J* = 12.0, 3.6 Hz, 1H), 3.10 – 3.01 (m, 1H), 2.38 – 2.30 (m, 1H), 1.13 (t, *J* = 7.2 Hz, 3H), 1.01 (d, *J* = 7.0 Hz, 3H), 0.82 (d, *J* = 7.2 Hz, 3H). ^{13}C NMR (100 MHz, $CDCl_3$) δ 184.71, 175.02, 149.52, 148.09, 135.02, 131.86, 130.60, 128.61, 128.28, 128.23, 65.24, 51.25, 49.04, 36.86, 28.11, 20.17, 18.71, 15.29. IR (cm^{-1}): 3057, 3033, 2962, 2934, 2874, 1678, 1629, 1498, 1454, 1402, 1376, 1310, 1262, 1140, 1125, 1064, 940, 866, 724, 700. HRMS: calcd. for $C_{20}H_{23}NO_2$ Na^+ $[M+Na]^+$: 332.1621; Found: 332.1597.

(4i) 1-benzyl-3-isopropyl-4-phenyl-1-azaspiro[4.5]deca-6,9-diene-2,8-dione

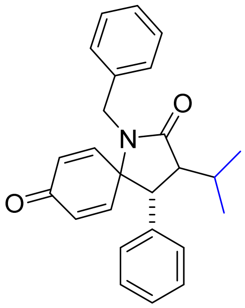

The title compound was prepared according to the general procedure described above by the reaction between *N*-benzyl-*N*-(4-hydroxyphenyl)cinnamamide (**1i**) with isobutyraldehyde (**2a**), and purified by flash column chromatography as yellow oil (27.8 mg, 75%).

^{1}H NMR (400 MHz, $CDCl_3$) δ 7.27 – 7.23 (m, 3H), 7.22 – 7.18 (m, 5H), 7.04 (dd, *J* = 6.8, 3.0 Hz, 2H), 6.54 (dd, *J* = 10.4, 3.2 Hz, 1H), 6.47 (dd, *J* = 10.2, 3.0 Hz, 1H), 6.18 (dd, *J* = 10.0, 2.0 Hz, 1H), 5.81 (dd, *J* = 10.2, 2.0 Hz, 1H), 4.66 (d, *J* = 15.0 Hz, 1H), 4.07 (d, *J* = 14.8 Hz, 1H), 3.42 (d, *J* = 12.0 Hz, 1H), 3.22 (dd, *J* = 12.0, 3.8 Hz, 1H), 2.43 – 2.35 (m, 1H), 1.07 (d, *J* = 6.8 Hz, 3H), 0.85 (d, *J* = 6.8 Hz, 3H). ^{13}C NMR (100 MHz, $CDCl_3$) δ 184.70, 175.31, 149.35, 147.30, 137.95, 134.66, 131.35, 130.71, 128.64, 128.60, 128.53, 128.30, 128.24, 127.78, 65.26, 51.27, 48.88, 45.36, 28.14, 20.11, 18.86. IR (cm^{-1}): 3061, 3031, 2961, 2930, 2873, 1670, 1629,1496, 1454, 1399, 1264, 1179, 1065, 1011, 958, 930, 863, 792, 734, 700. HRMS: calcd. for $C_{25}H_{25}NO_2$ Na^+ $[M+Na]^+$: 394.1778; Found: 394.1761.

(4j) 3-isopropyl-1-methyl-4-(naphthalen-2-yl)-1-azaspiro[4.5]deca-6,9-diene-2,8-dione

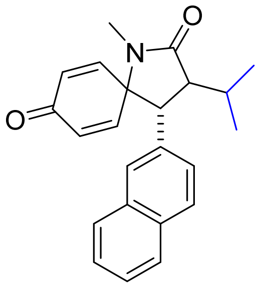

The title compound was prepared according to the general procedure described above by the reaction between*N*-(4-hydroxyphenyl)-*N*-methyl-3-(naphthalen-2-yl)acrylamide (**1j**) with isobutyraldehyde (**2a**), and purified by flash column chromatography as white solid (21.7 mg, 62%).

M.p. 84.4 – 86.8 °C. ^{1}H NMR (400 MHz, $CDCl_3$) δ 7.80 – 7.73 (m, 3H), 7.58 – 7.54 (m, 1H), 7.50 – 7.45 (m, 2H), 7.22 (dd, *J* = 8.4, 2.0 Hz, 1H), 6.84 (dd, *J* = 10.2, 3.2 Hz, 1H), 6.63 (dd, *J* = 10.2, 3.0 Hz, 1H), 6.41 (dd, *J* = 10.2, 2.0 Hz, 1H), 5.94 (dd, *J* = 10.2, 2.0 Hz, 1H), 3.61 (d, *J* = 11.8 Hz, 1H), 3.27 (dd, *J* = 11.8, 3.8 Hz, 1H), 2.76 (s, 3H), 2.42 – 2.34(m, 1H), 1.04 (d, *J* = 6.8 Hz, 3H), 0.83 (d, *J* = 7.0 Hz, 3H). ^{13}C NMR (100 MHz, $CDCl_3$) δ 184.38, 175.20, 149.49, 146.96, 133.11, 132.76, 132.40, 131.58, 133.02, 128.46, 127.88, 127.80, 127.46, 126.63, 126.46, 125.83, 65.04, 50.97, 49.34, 28.21, 27.15, 20.25, 18.80. IR (cm^{-1}): 3052, 2960, 2931, 2874, 1691, 1672, 1629, 1466, 1418, 1392, 1374, 1261, 1173, 1141, 1064, 992, 854, 822, 735, 607. HRMS: calcd. for $C_{23}H_{23}NO_2$ Na^+ $[M+Na]^+$: 368.1621; Found: 368.1605.

(4k) 4-(furan-2-yl)-3-isopropyl-1-methyl-1-azaspiro[4.5]deca-6,9-diene-2,8-dione

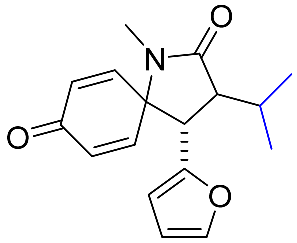

The title compound was prepared according to the general procedure described above by the reaction between 3-(furan-2-yl)-*N*-(4-hydroxyphenyl)-*N*-methylacrylamide (**1k**) with isobutyraldehyde (**2a**), and purified by flash column chromatography as yellow oil (20.5 mg, 72%).

^{1}H NMR (400 MHz, $CDCl_3$) δ 7.29 – 7.28 (m, 1H), 6.70 (dd, *J* = 10.0, 3.1 Hz, 1H), 6.63 (dd, *J* = 10.2, 3.0 Hz, 1H), 6.43 (dd, *J* = 10.0, 2.0 Hz, 1H), 6.24 (dd, *J* = 3.2, 2.0 Hz, 1H), 6.10 – 6.07 (m, 2H), 3.48 (d, *J* = 11.8 Hz, 1H), 3.17 (dd, *J* = 12.0, 4.0 Hz, 1H), 2.72 (s, 3H), 2.42 – 2.34 (m, 1H), 0.98 (d, *J* = 6.8 Hz, 3H), 0.86 (d, *J* = 7.2 Hz, 3H). ^{13}C NMR (100 MHz, $CDCl_3$) δ 184.51, 174.69, 149.14, 149.04, 146.70, 142.45, 132.12, 131.24, 110.67, 108.59, 64.25, 48.72, 44.06, 27.76, 27.04, 19.70, 18.14. IR (cm^{-1}): 3049, 2962, 2933, 2876, 1697, 1674, 1631, 1505, 1420, 1390, 1373, 1261, 1173, 1149, 1118, 1065, 1021, 859, 736, 599. HRMS: calcd. for $C_{17}H_{19}NO_3$ Na^+ $[M+Na]^+$: 308.1257; Found: 308.1248.

(6) 3-isopropyl-6-methoxy-1-methyl-4-phenyl-3,4-dihydroquinolin-2(1H)-one

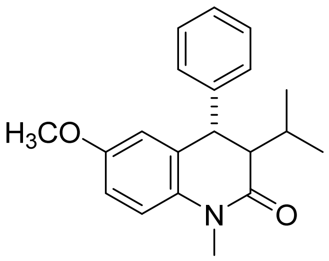

The title compound was prepared according to the general procedure described above by the reaction between *N*-(4-methoxyphenyl)-*N*-methylcinnamamide with isobutyraldehyde (**2a**), and purified by flash column chromatography as colorless oil (22.9 mg, 74%).

^{1}H NMR (400 MHz, $CDCl_3$) δ 7.24 – 7.13 (m, 3H), 7.00 – 6.97 (m, 3H), 6.85 (dd, *J* = 8.8, 3.2 Hz, 1H), 6.75 (d, *J* = 2.8 Hz, 1H), 4.13 (s, 1H), 3.78 (s, 3H), 3.33 (s, 3H), 2.57 (dd, *J* = 9.2, 2.0 Hz, 1H), 1.70 – 1.63 (m, 1H), 1.04 (d, *J* = 6.6 Hz, 3H), 0.98 (d, *J* = 6.8 Hz, 3H). ^{13}C NMR (100 MHz, $CDCl_3$) δ 170.31, 155.69, 141.99, 133.86, 128.82, 128.23, 127.19, 126.84, 115.88, 115.52, 112.61, 56.64, 55.56, 45.37, 29.73, 28.55, 21.19, 21.05. IR (cm^{-1}): 3060, 3025, 2960, 2933, 2872, 2835, 1731, 1666, 1590,1503, 1469, 1432, 1387, 1341, 1249, 1034, 910, 810, 699, 621. HRMS: calcd. for $C_{20}H_{23}NO_2$ Na^+ $[M+Na]^+$: 332.1621; Found: 332.1610.

(5) 2, 6-di-*tert*-butyl-4-isopropyl-4-methylcyclohexa-2,5-dienone[2]

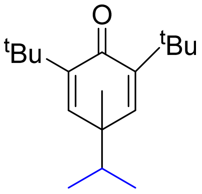

^{1}H NMR (400 MHz, $CDCl_3$) δ 6.44 (s, 2H), 1.80 – 174 (m, 1H), 1.24 (s, 18H), 1.17 (s, 3H), 0.84 (d, *J* = 6.8 Hz, 6H). ^{13}C NMR (100 MHz, $CDCl_3$) δ 186.90, 146.95, 145.80, 42.43, 37.55, 34.89, 29.70, 24.72, 18.09. IR (cm^{-1}): 3001, 2961, 2876, 1658, 1643, 1460, 1374, 1267, 1061, 867, 751.

IV. References

1. Zhang, H.-L.; Gu, Z.-X.; Xu, P.; Hu, H.-W.; Cheng, Y.-X.; Zhu, C.-J. Metal-free tandem oxidative C(sp3)-H bond functionalization of alkanes and dearomatization of *N*-phenyl-cinnam-amides: access to alkylated 1-azaspiro[4.5]decanes. *Chem. Commun*. **2016**, *52, 477-480.*
2. Gao, R.-X.; Luan, X.-Q.; Xie, Z.-Y.; Yang, L.; Pei, Y. Fe-Catalyzed decarbonylative cascade reaction of *N*-aryl cinnamamides with aliphatic aldehydes to construct 3,4-dihydroquinolin-2(1H)-ones. *Org. Biomol. Chem.* **2019**, *17, 5262-5268.*

V. Copies of ^{1}H and ^{13}C NMR spectra of products 3a-3l, 4b-4k, 6, 5

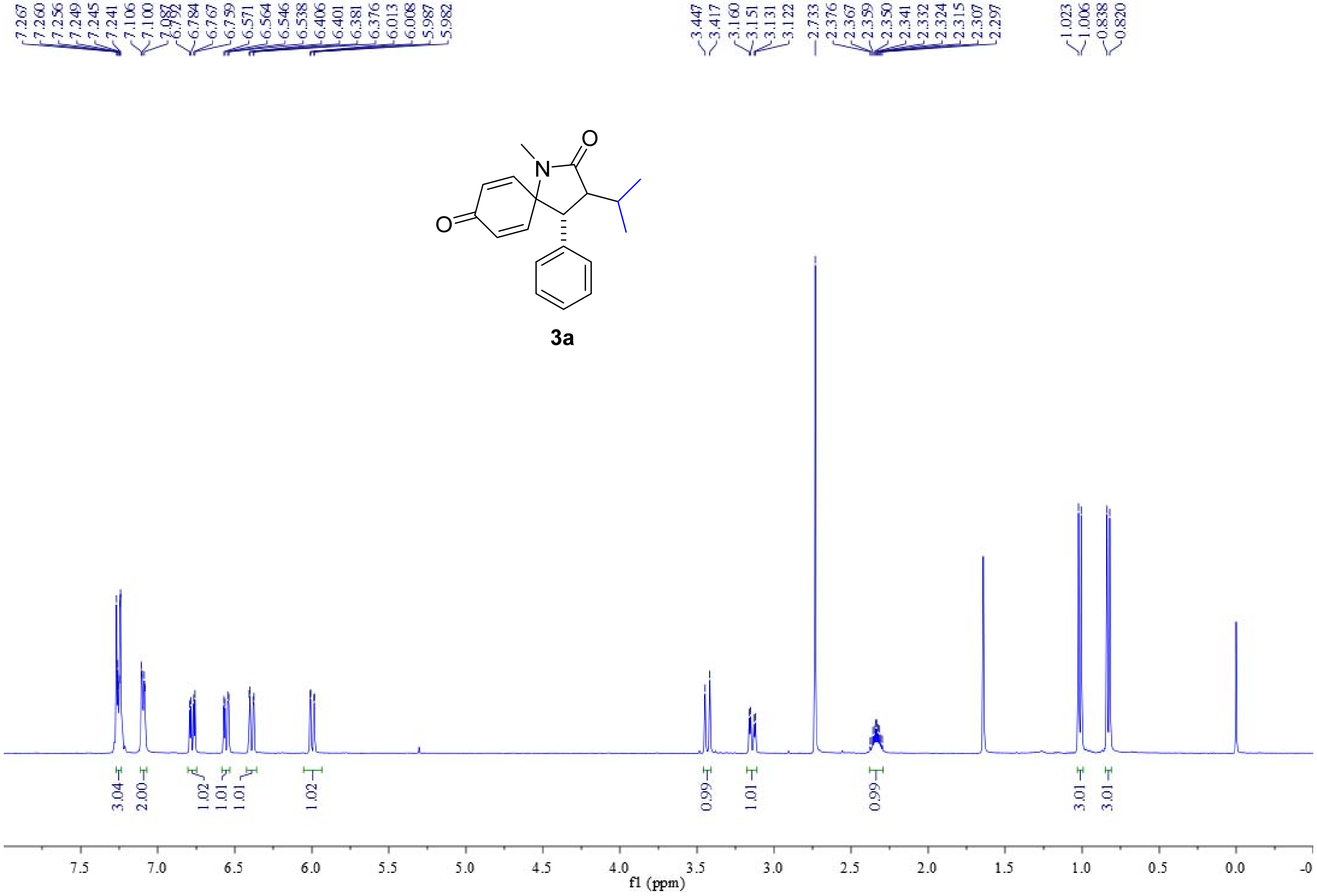

3a
f1 (ppm)

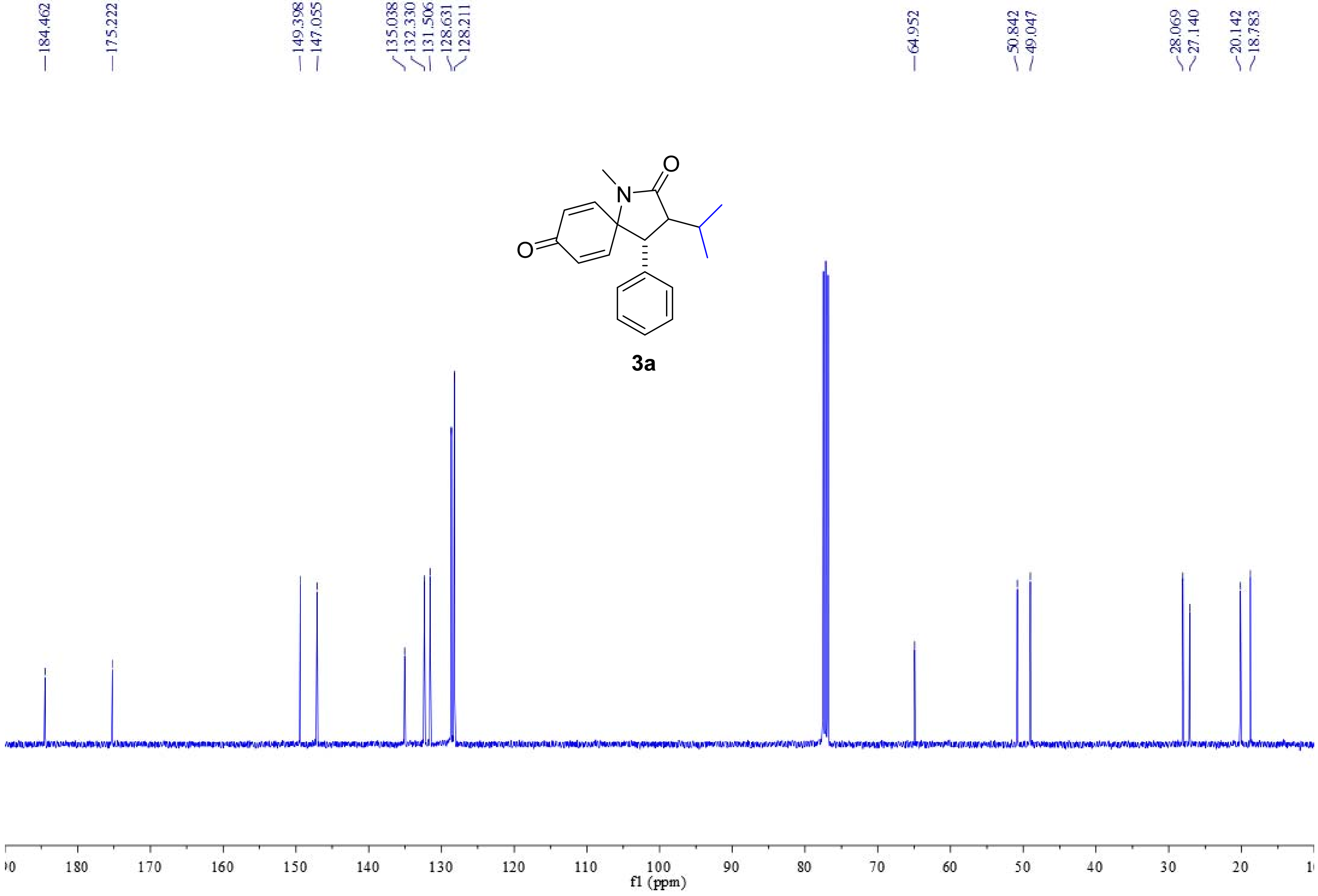
184.462
175.222
149.398
147.055
135.038
132.330
131.506
128.631
128.211
64.952
50.842
49.047
28.069
27.140
20.142
18.783
O
N
O
3a
180
170
160
150
140
130
120
110
100
90
80
70
60
50
40
30
20
f1 (ppm)

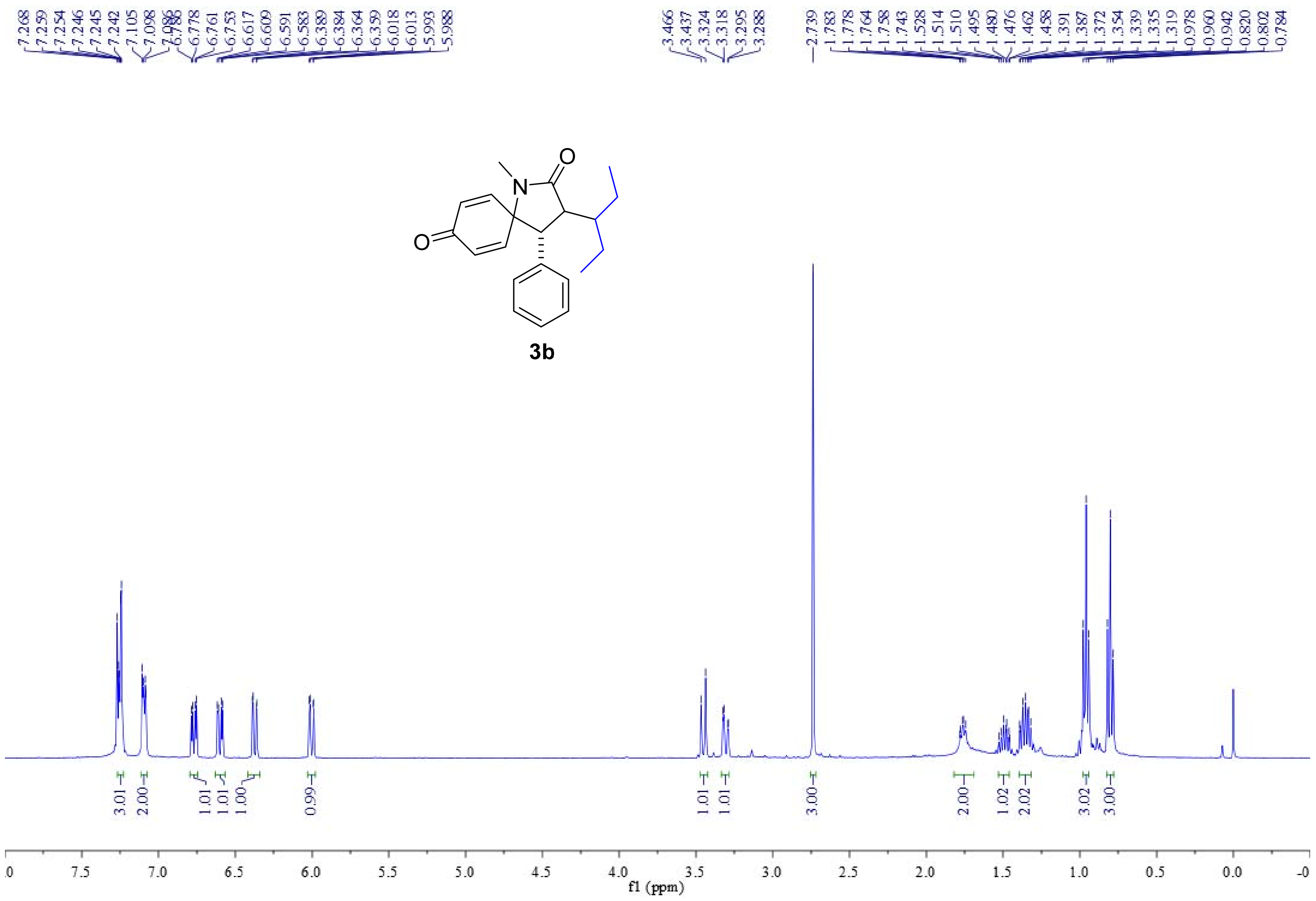
3b
f1 (ppm)

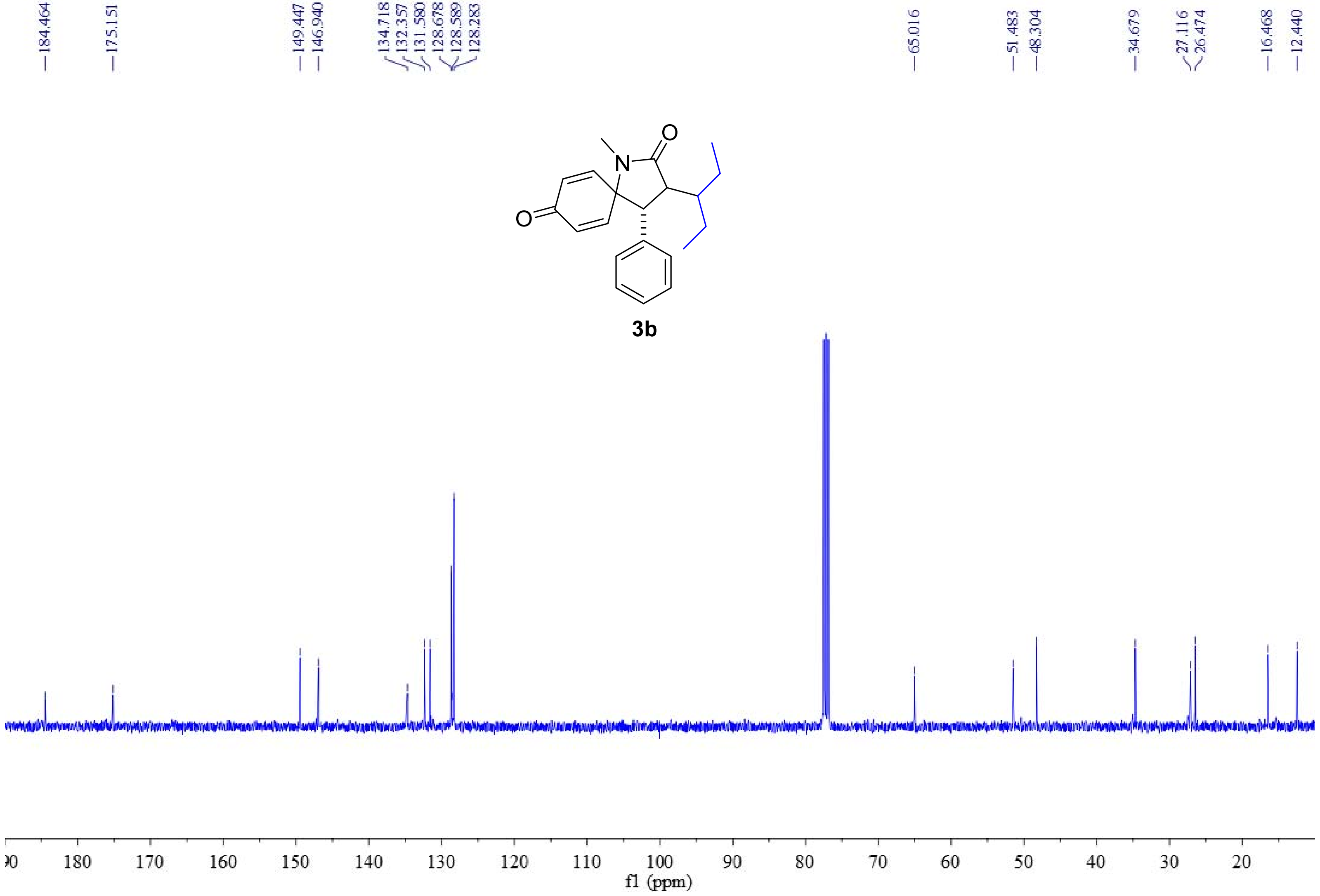
184.464
175.151
149.447
146.940
134.718
132.357
131.580
128.678
128.589
128.283
65.016
51.483
48.304
34.679
27.116
26.474
16.468
12.440
O
N
O
3b
90
180
170
160
150
140
130
120
110
100
90
80
70
60
50
40
30
20
f1 (ppm)

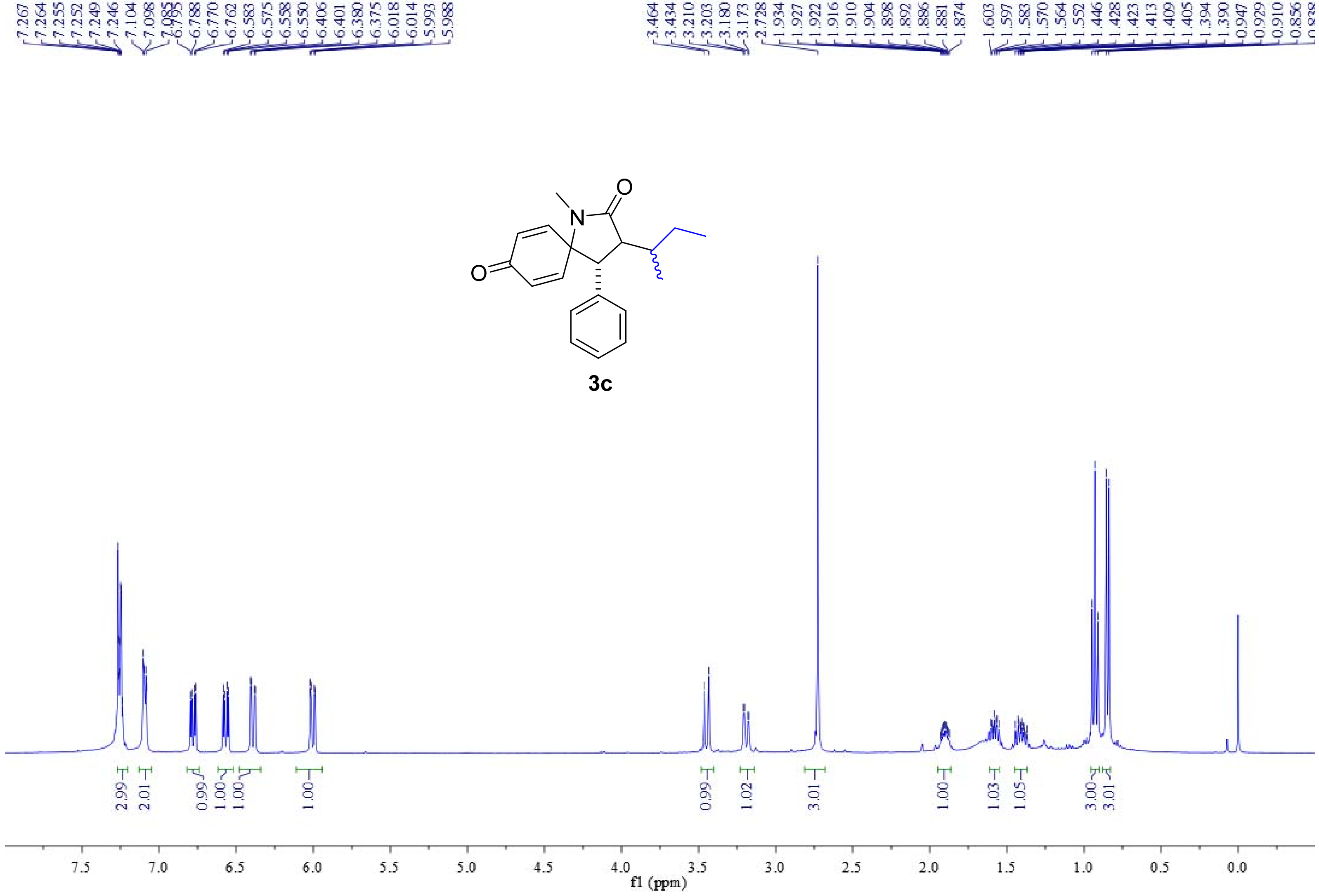

3c

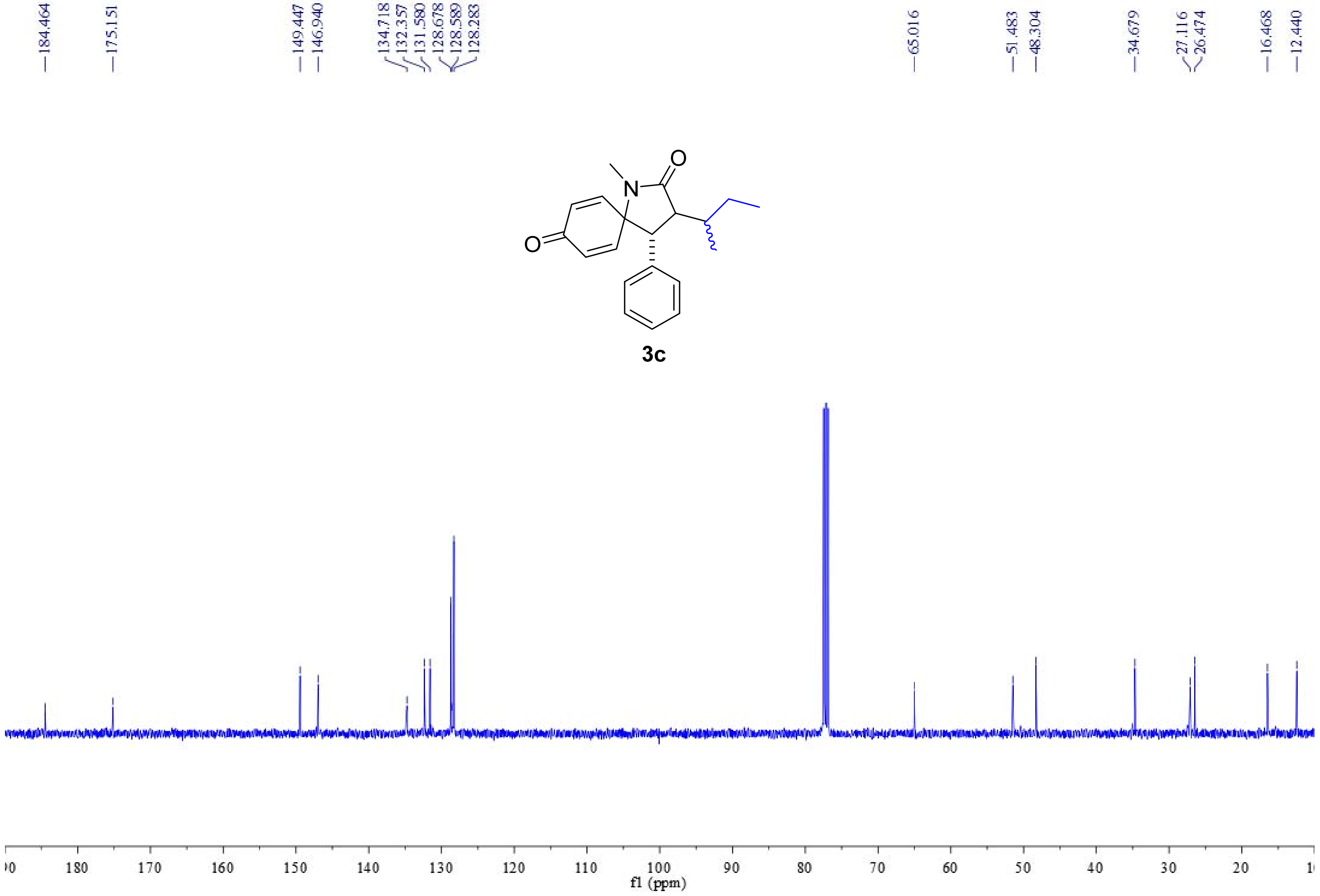
184.464
175.151
149.447
146.940
134.718
132.357
131.580
128.678
128.589
128.283
65.016
51.483
48.304
34.679
27.116
26.474
16.468
12.440
O
N
O
3c
180
170
160
150
140
130
120
110
100
90
80
70
60
50
40
30
20
f1 (ppm)

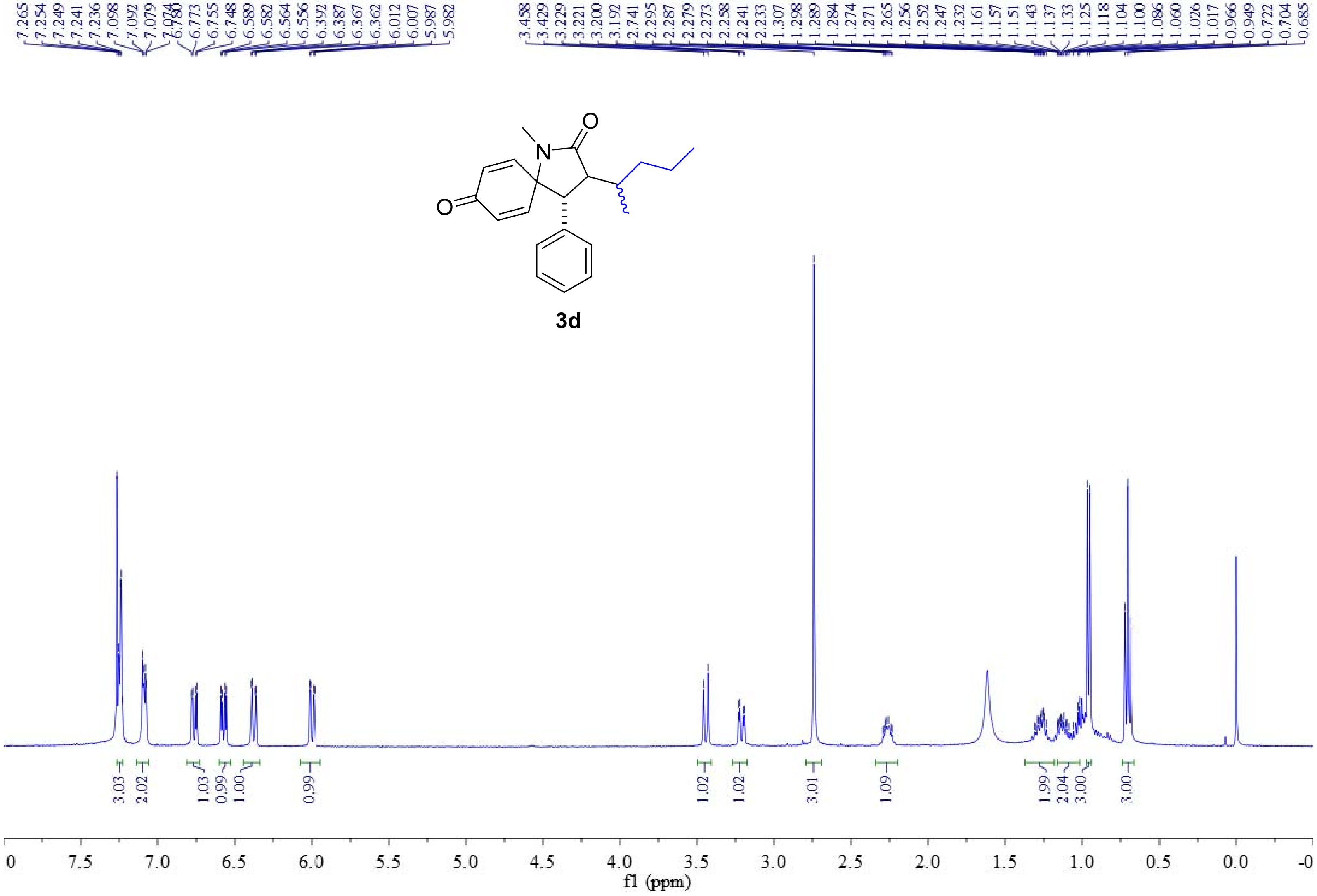

3d

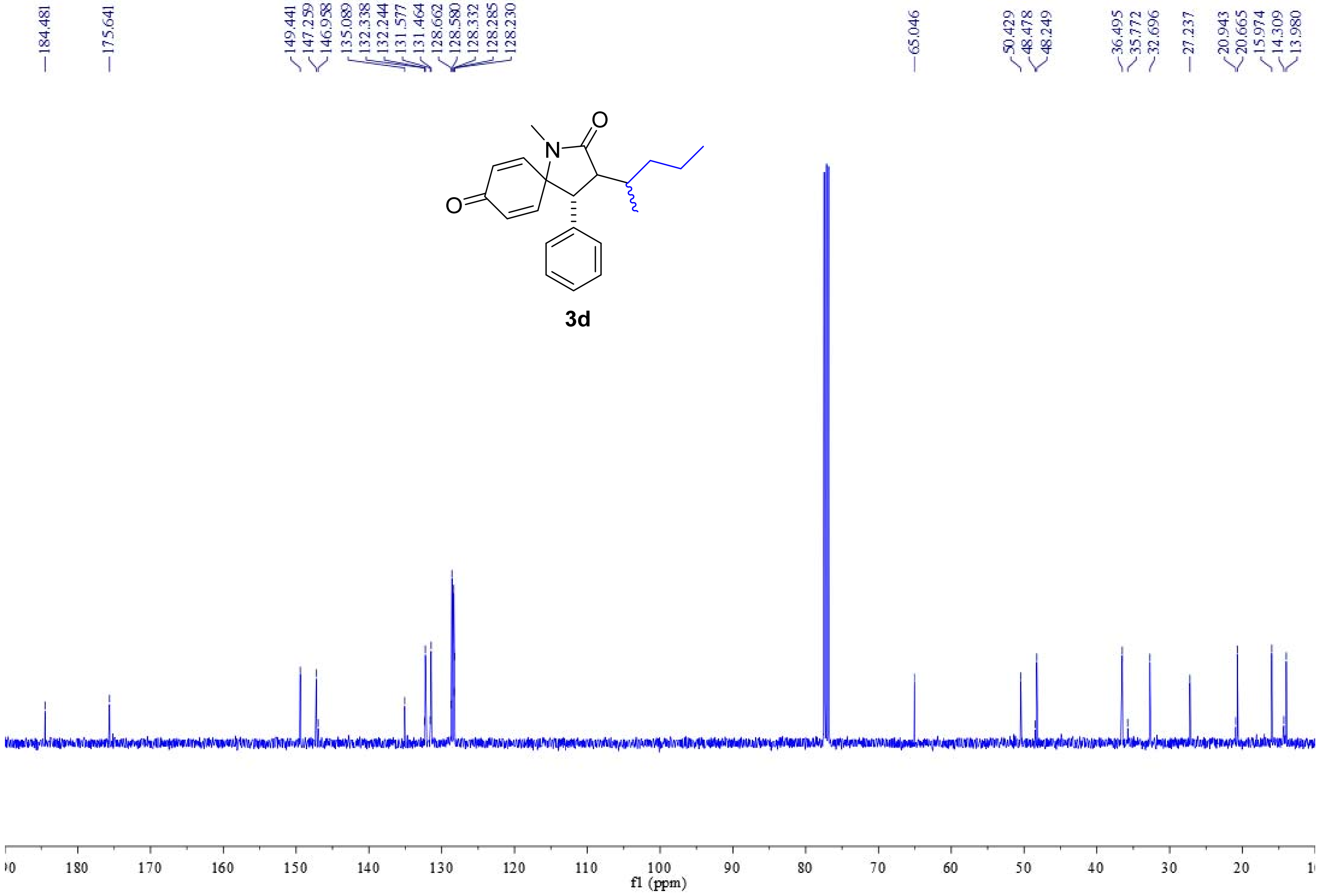
184.481
175.641
149.441
147.259
146.958
135.089
132.338
132.244
131.577
131.464
128.662
128.580
128.332
128.285
128.230
65.046
50.429
48.478
48.249
36.495
35.772
32.696
27.237
20.943
20.665
15.974
14.309
13.980
N
O
O
3d
180
170
160
150
140
130
120
110
100
90
80
70
60
50
40
30
20
f1 (ppm)

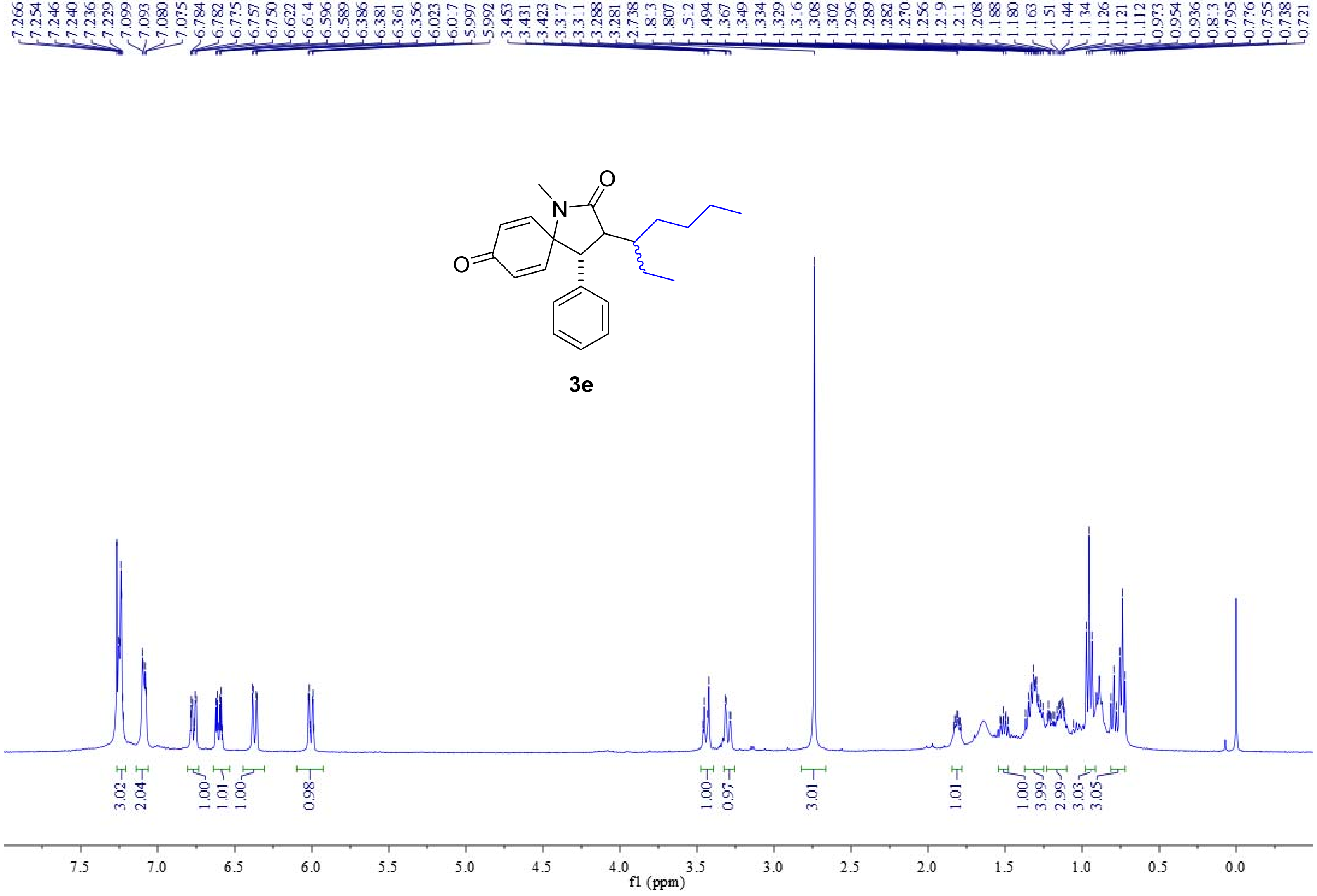

3e

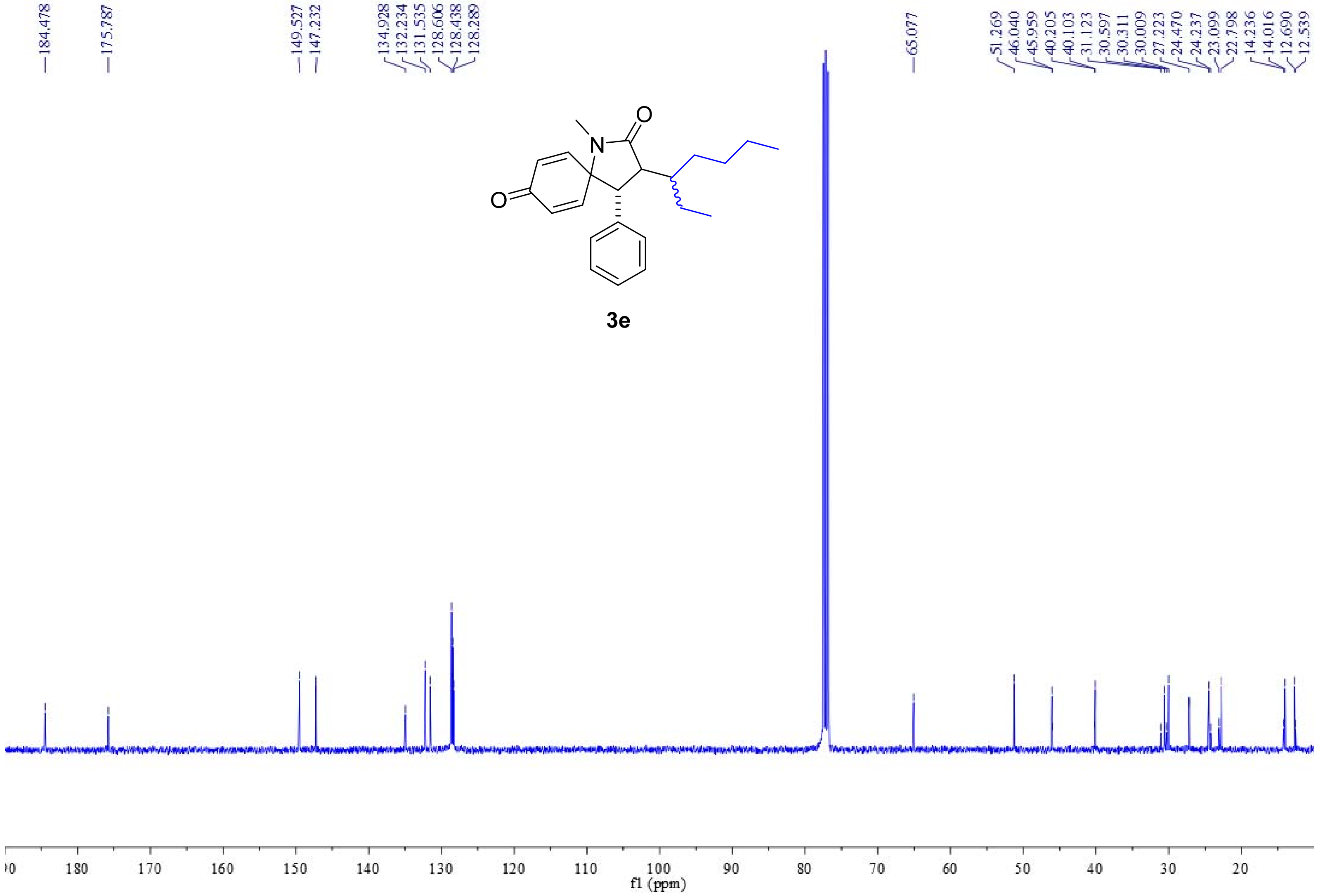
184.478
175.787
149.527
147.232
134.928
132.234
131.535
128.606
128.438
128.289
65.077
51.269
46.040
45.959
40.205
40.103
31.123
30.597
30.311
30.009
27.223
24.470
24.237
23.099
22.798
14.236
14.016
12.690
12.539
O
N
O
3e
180
170
160
150
140
130
120
110
100
90
80
70
60
50
40
30
20
f1 (ppm)

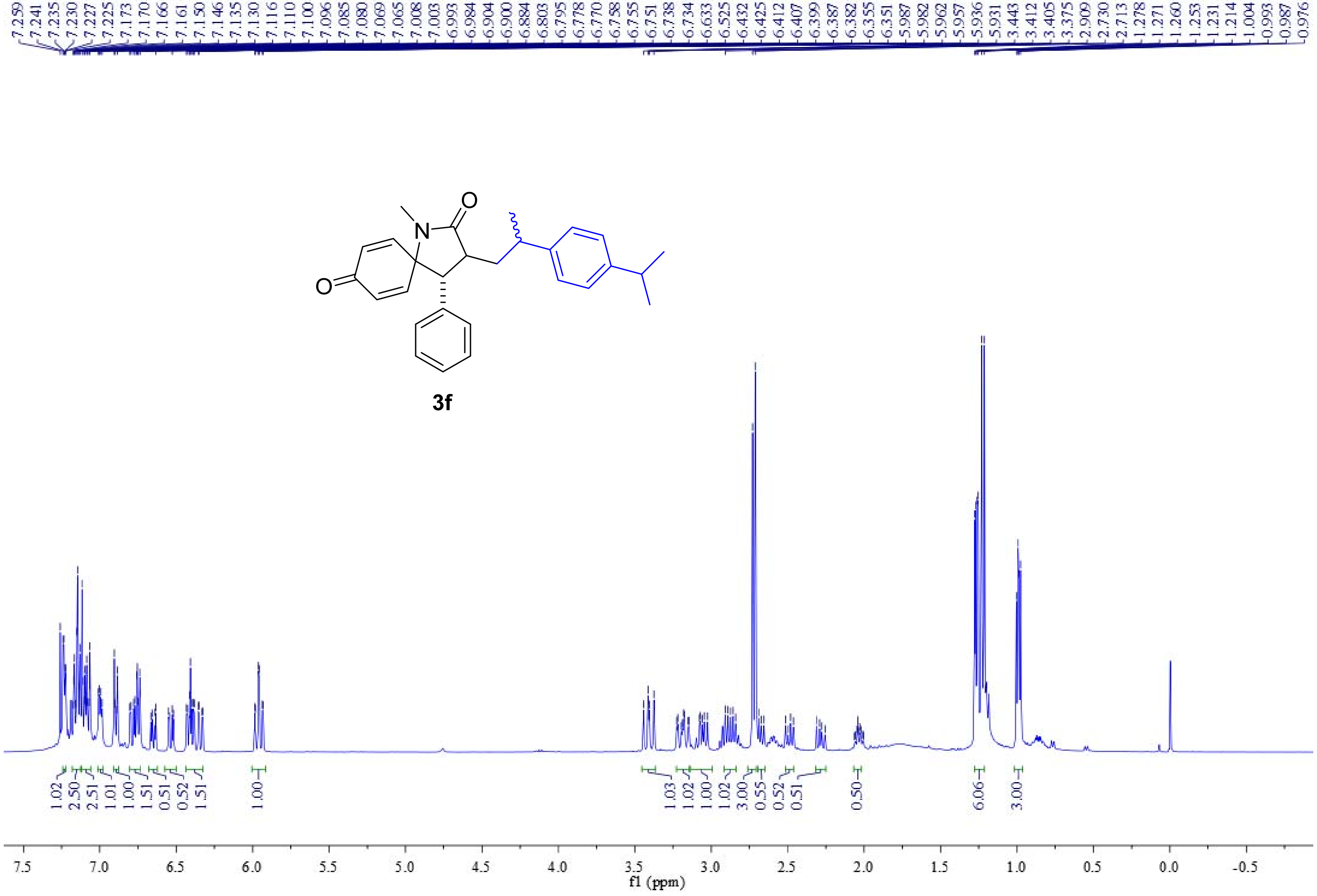

3f

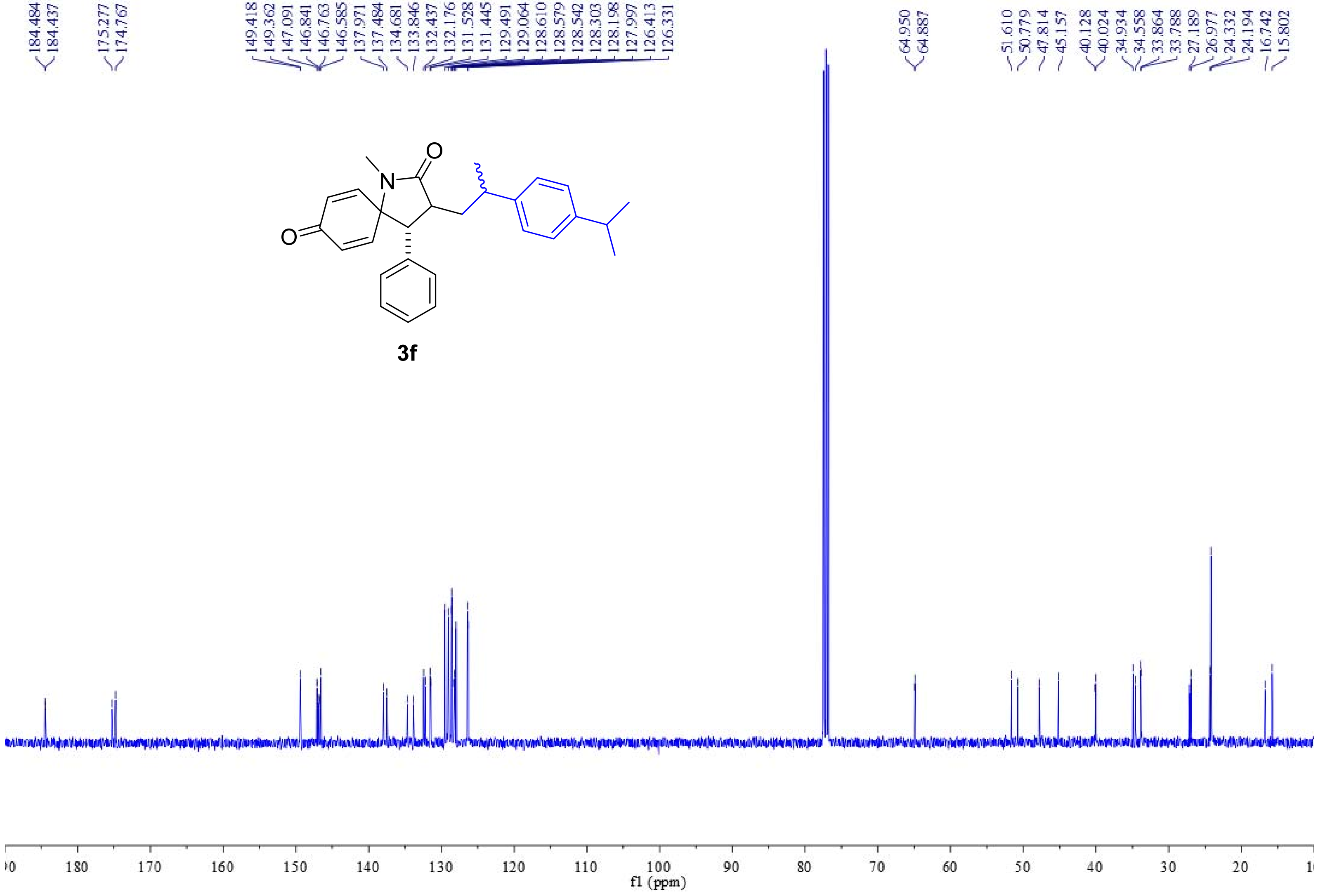
184.484
184.437
175.277
174.767
149.418
149.362
147.091
146.841
146.763
146.585
137.971
137.484
134.681
133.846
132.437
132.176
131.528
131.445
129.491
129.064
128.610
128.579
128.542
128.303
128.198
127.997
126.413
126.331
64.950
64.887
51.610
50.779
47.814
45.157
40.128
40.024
34.934
34.558
33.864
33.788
27.189
26.977
24.332
24.194
16.742
15.802
3f
f1 (ppm)

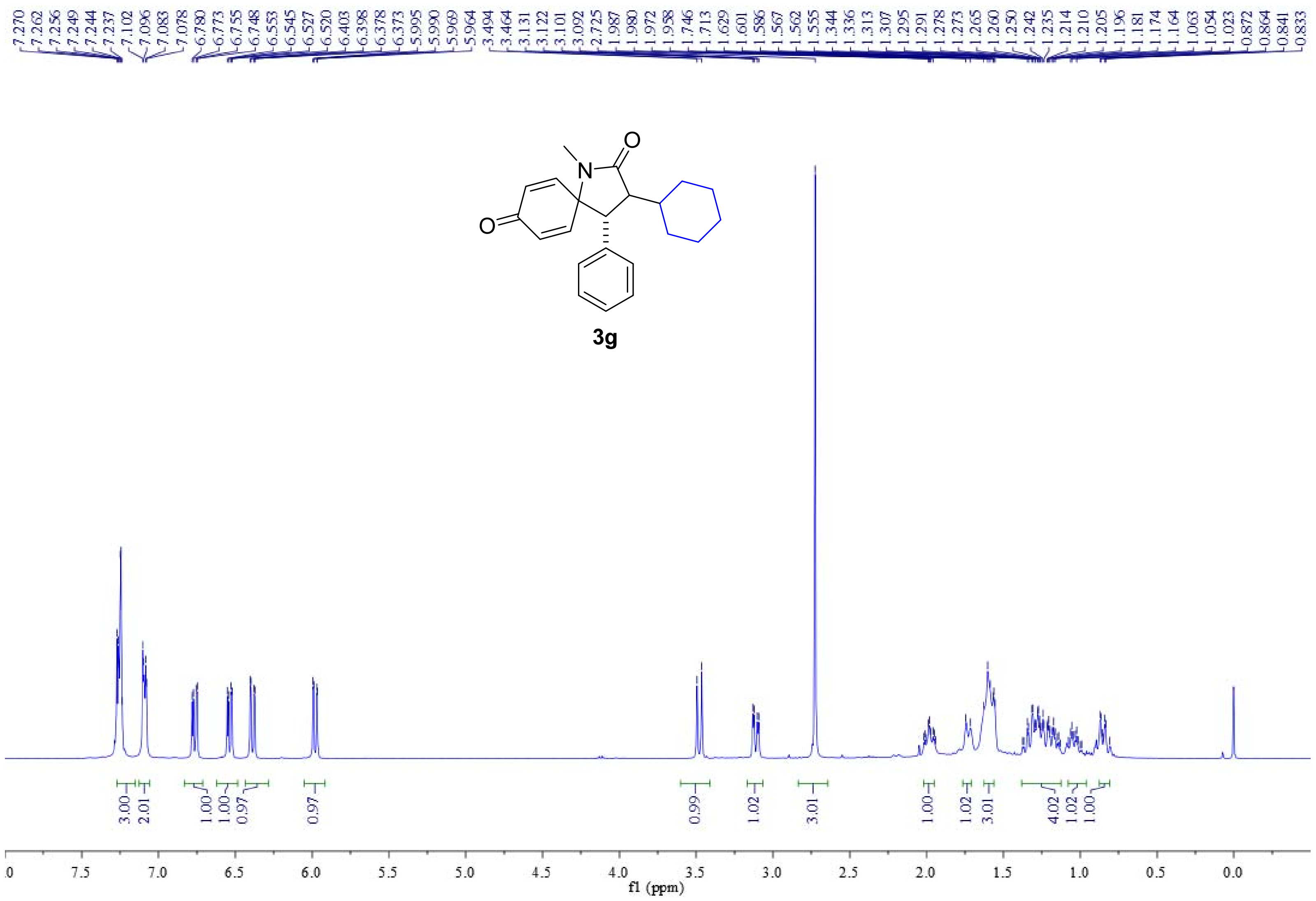

3g

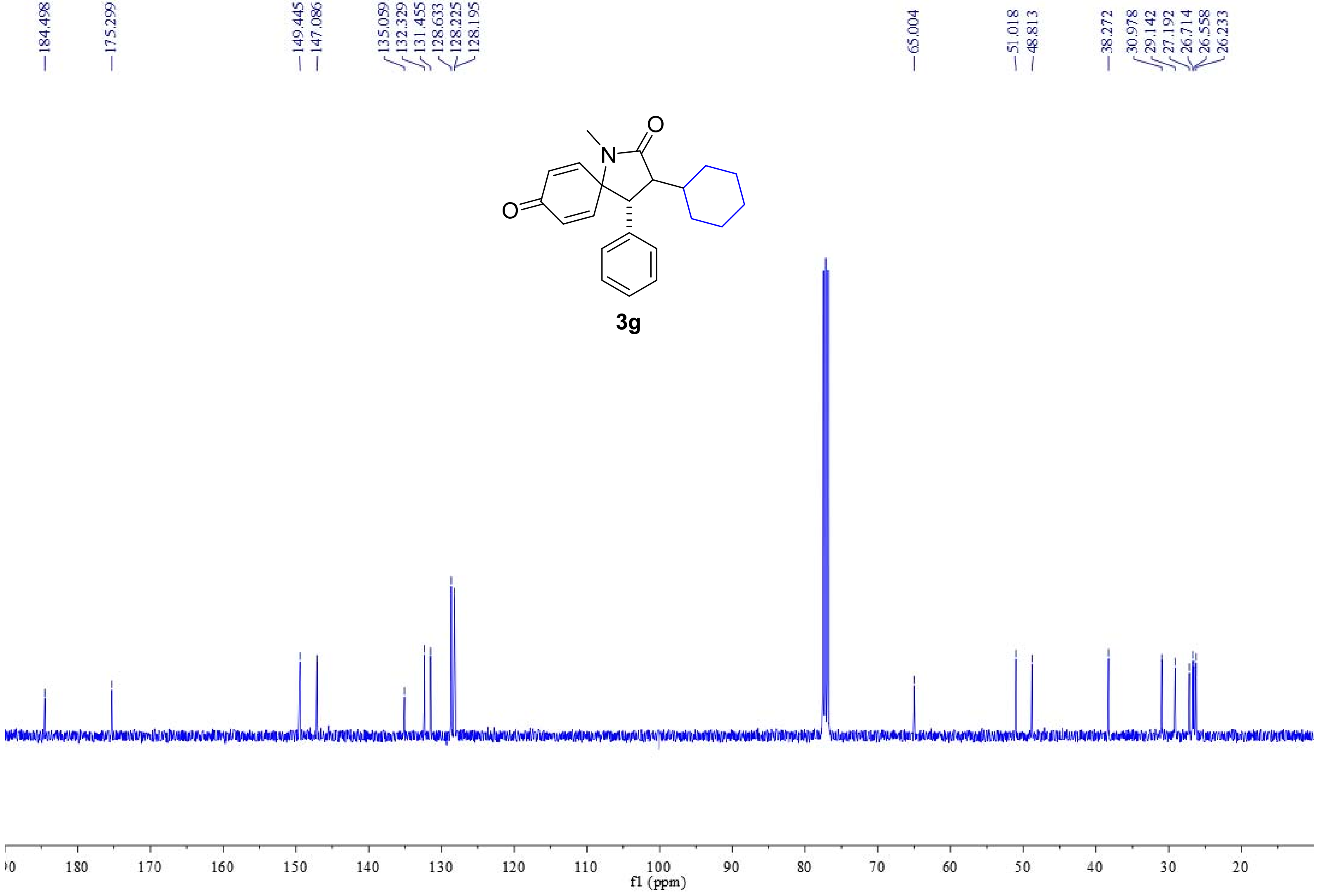
184.498
175.299
149.445
147.086
135.059
132.329
131.455
128.633
128.225
128.195
65.004
51.018
48.813
38.272
30.978
29.142
27.192
26.714
26.558
26.233
O
N
O
3g
180 170 160 150 140 130 120 110 100 90 80 70 60 50 40 30 20
f1 (ppm)

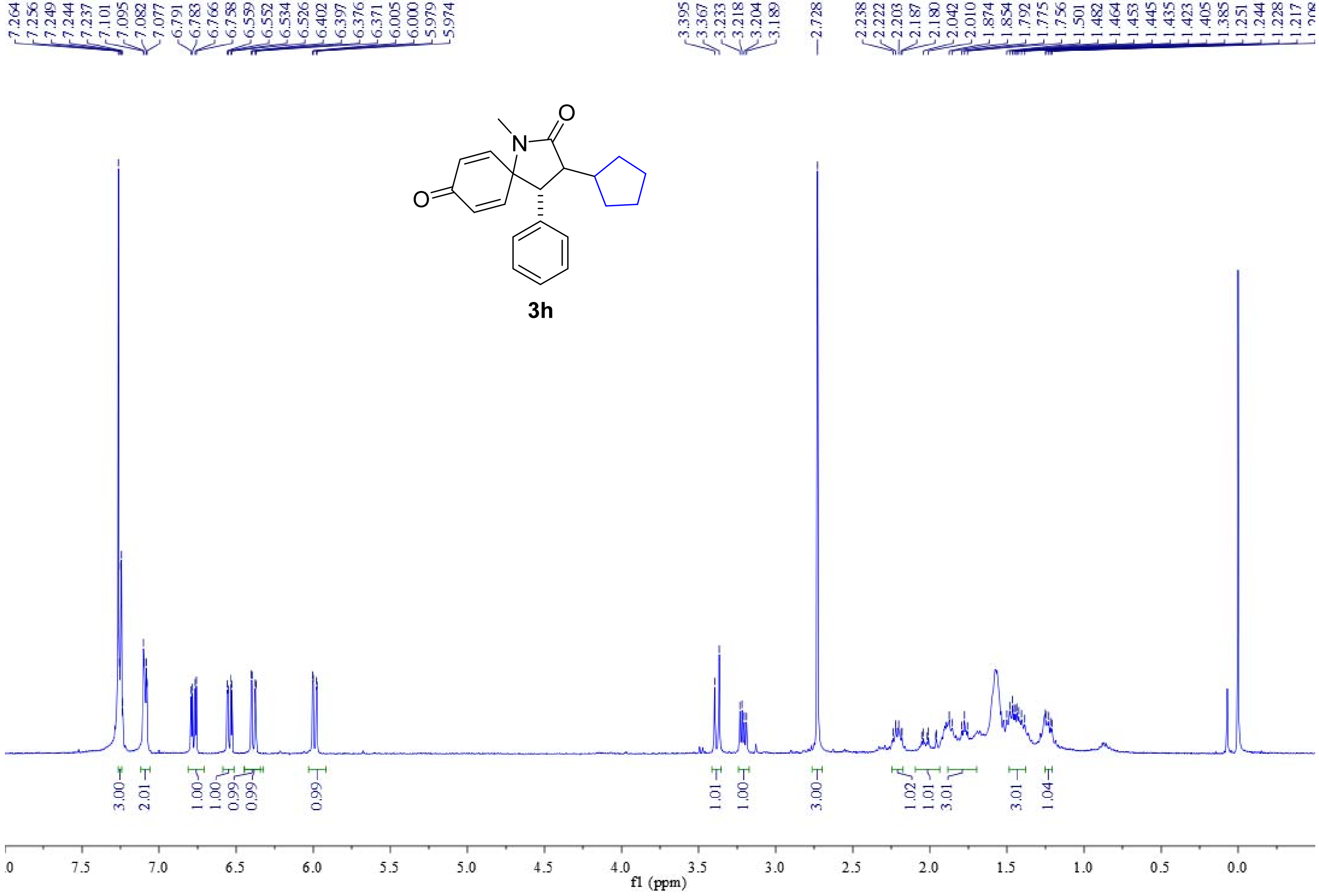
3h
f1 (ppm)

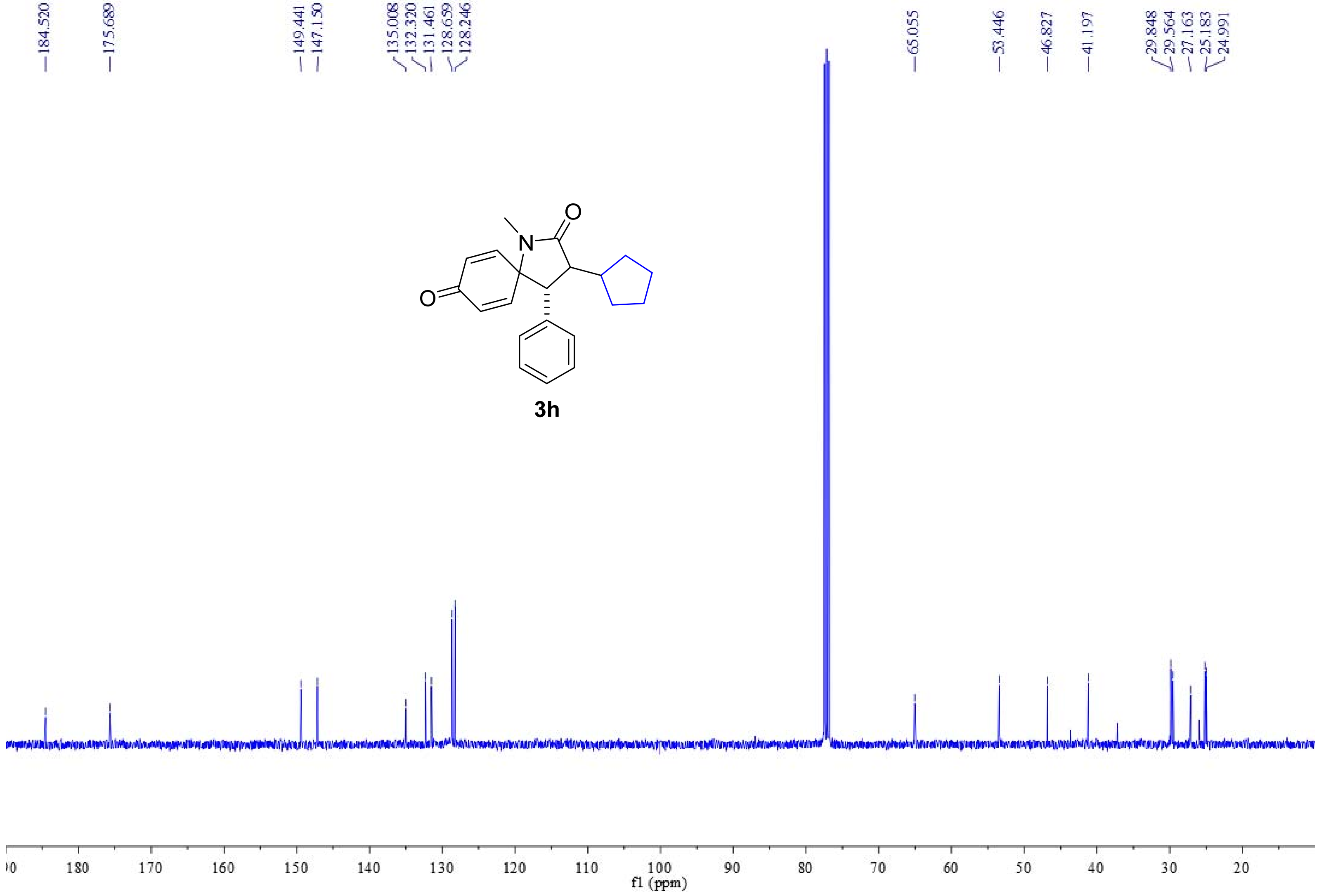
184.520
175.689
149.441
147.150
135.008
132.320
131.461
128.659
128.246
65.055
53.446
46.827
41.197
29.848
29.564
27.163
25.183
24.991
O
N
O
3h
180 170 160 150 140 130 120 110 100 90 80 70 60 50 40 30 20
f1 (ppm)

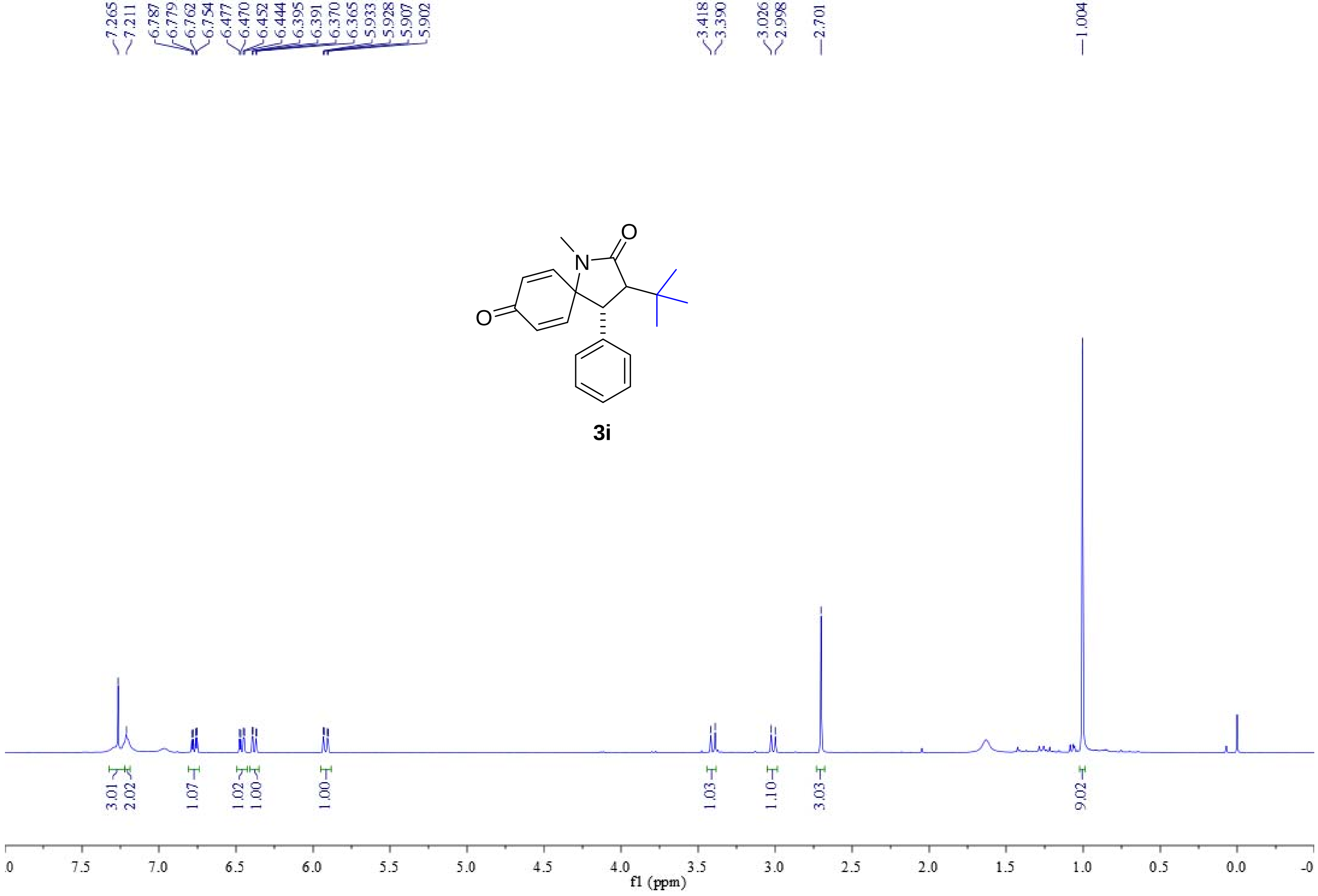

7.265
7.211
6.787
6.779
6.762
6.754
6.477
6.470
6.452
6.444
6.395
6.391
6.370
6.365
5.933
5.928
5.907
5.902
3.418
3.390
3.026
2.998
2.701
1.004
3i
3.01
2.02
1.07
1.02
1.00
1.00
1.03
1.10
3.03
9.02
f1 (ppm)

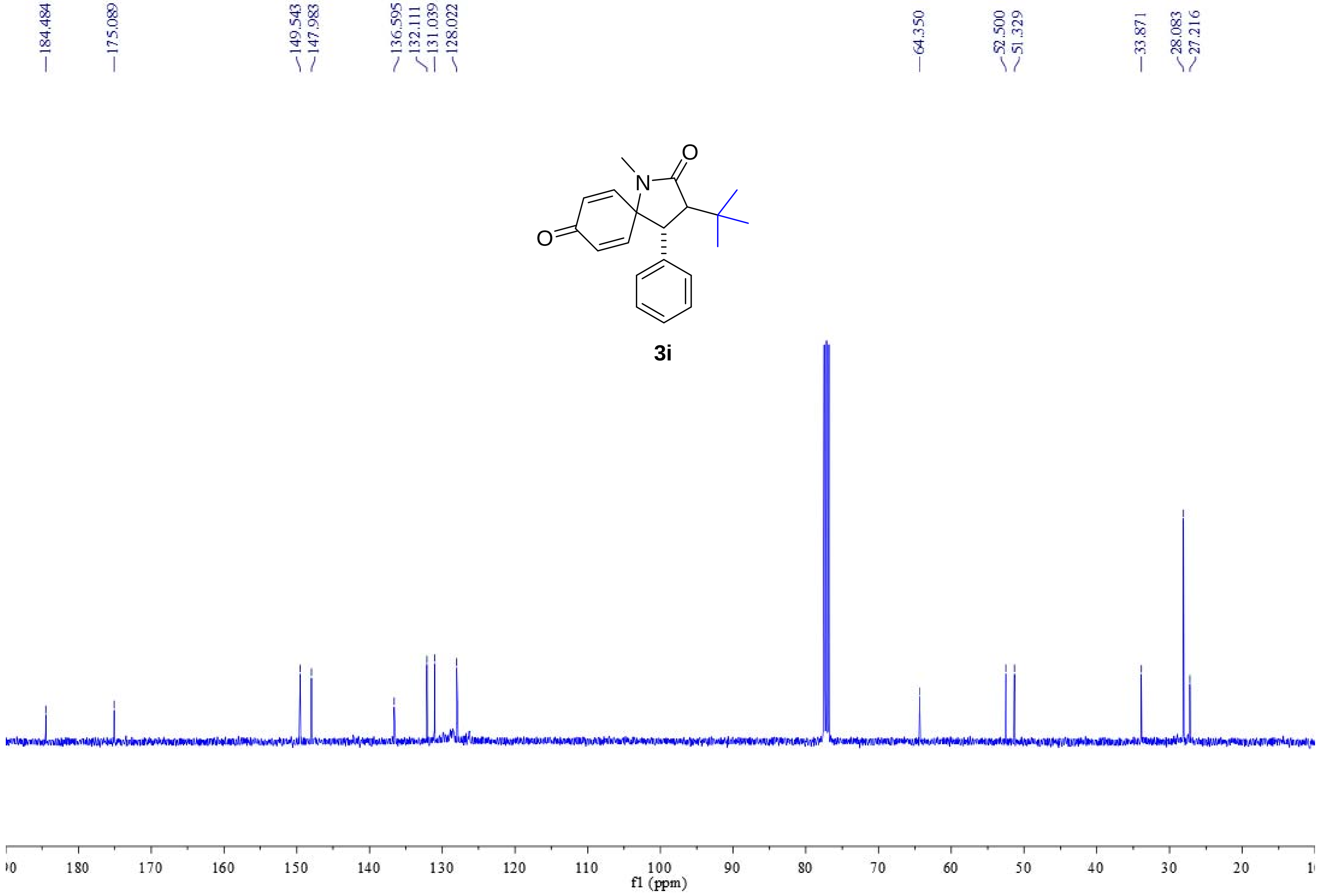
184.484
175.089
149.543
147.983
136.595
132.111
131.039
128.022
64.350
52.500
51.329
33.871
28.083
27.216
3i
f1 (ppm)

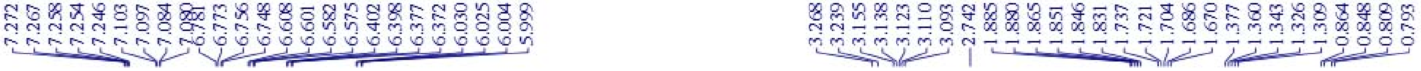

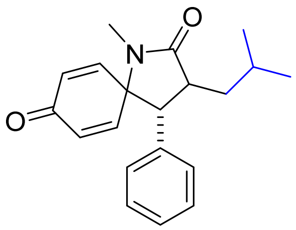

3j

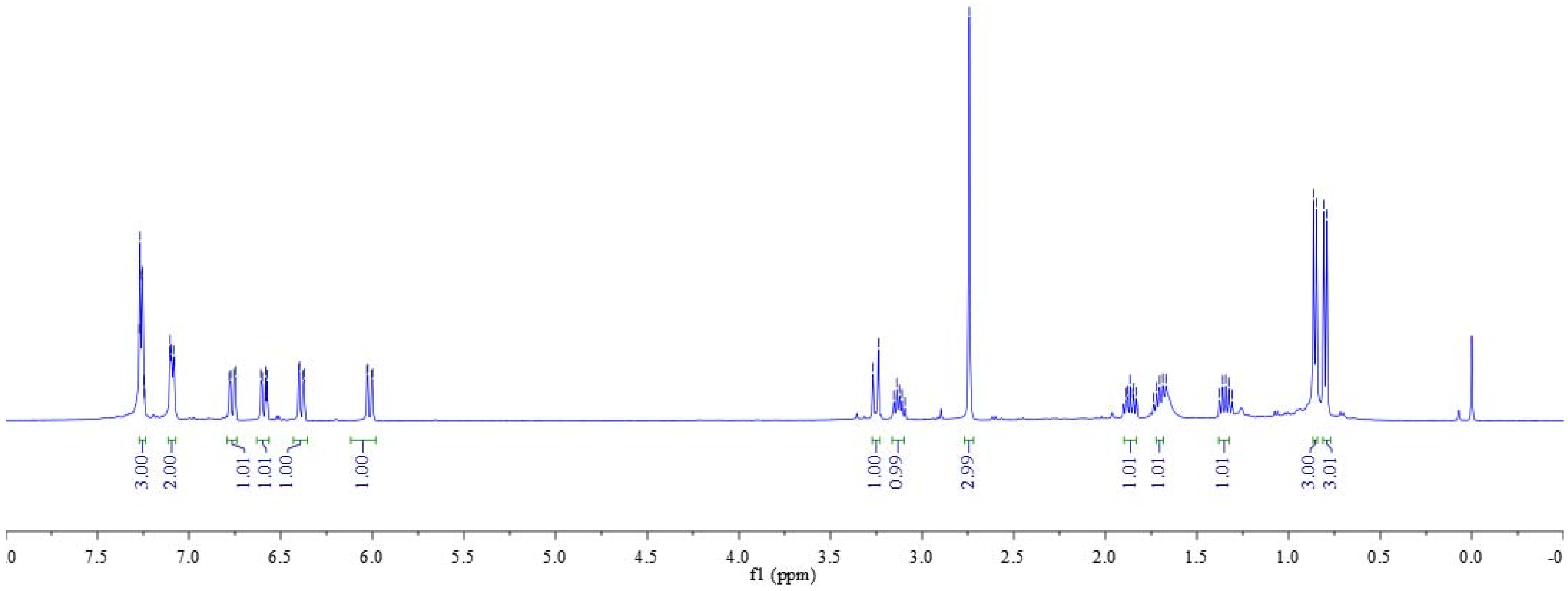

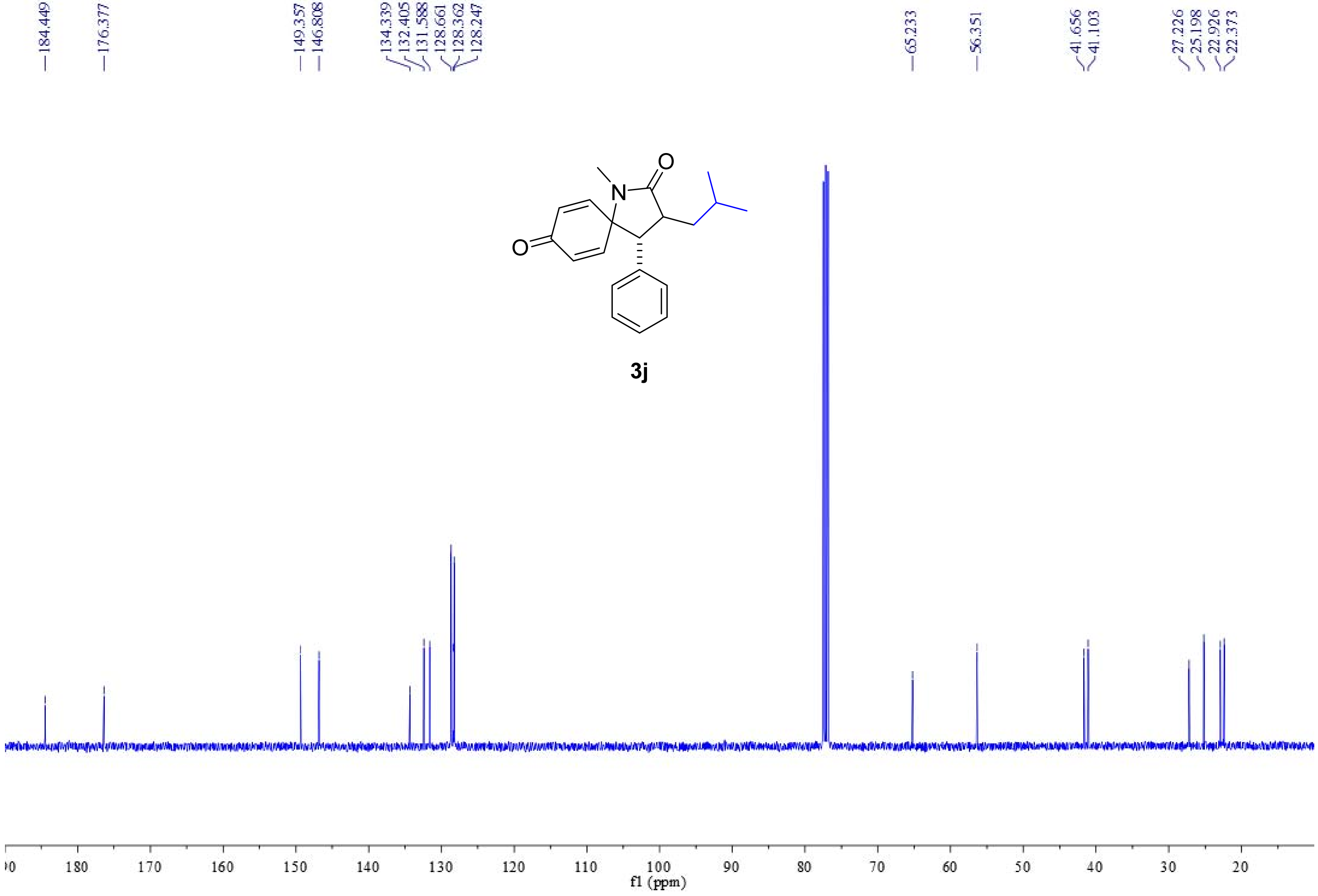
184.449
176.377
149.357
146.808
134.339
132.405
131.588
128.661
128.362
128.247
65.233
56.351
41.656
41.103
27.226
25.198
22.926
22.373
3j
f1 (ppm)

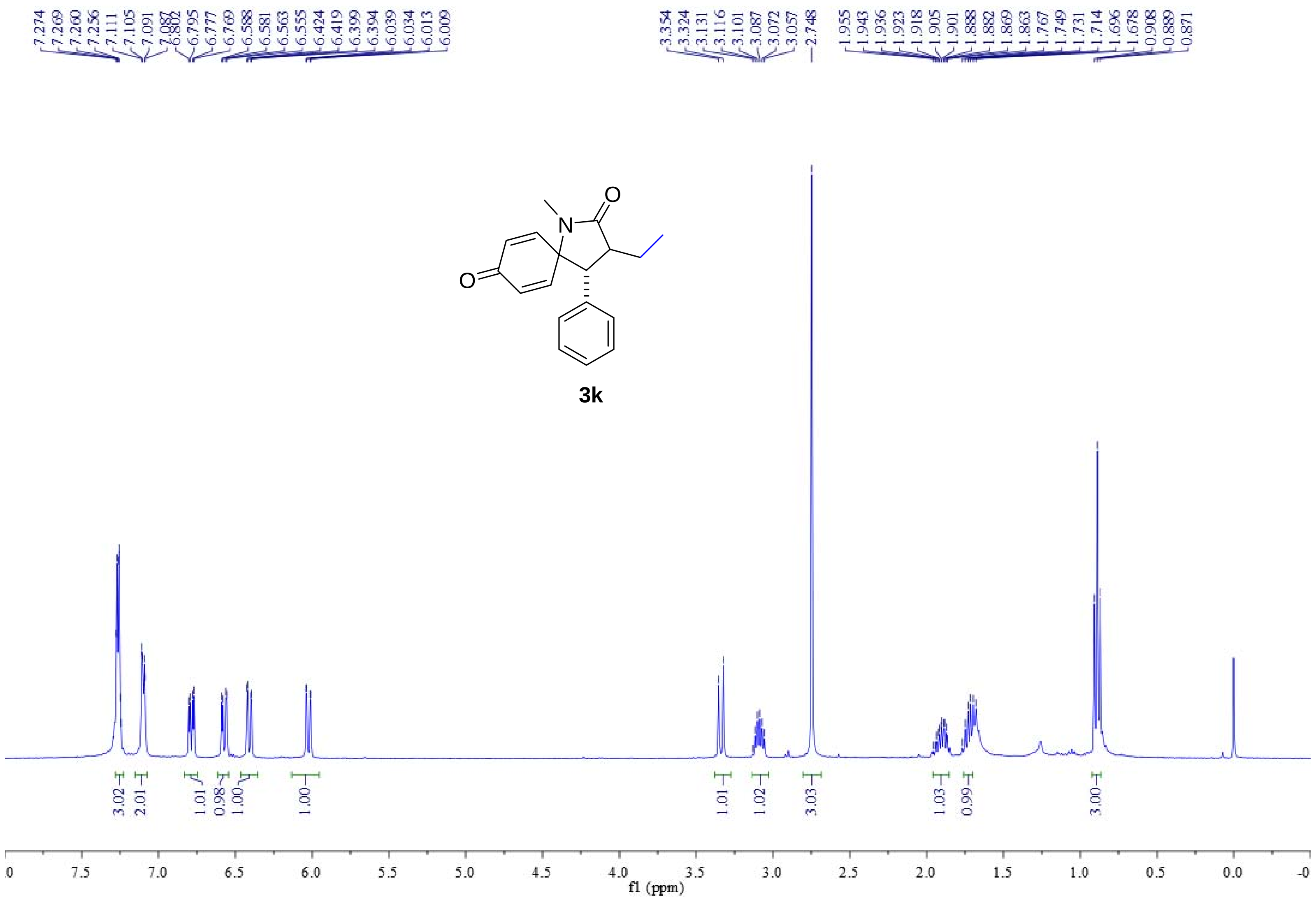

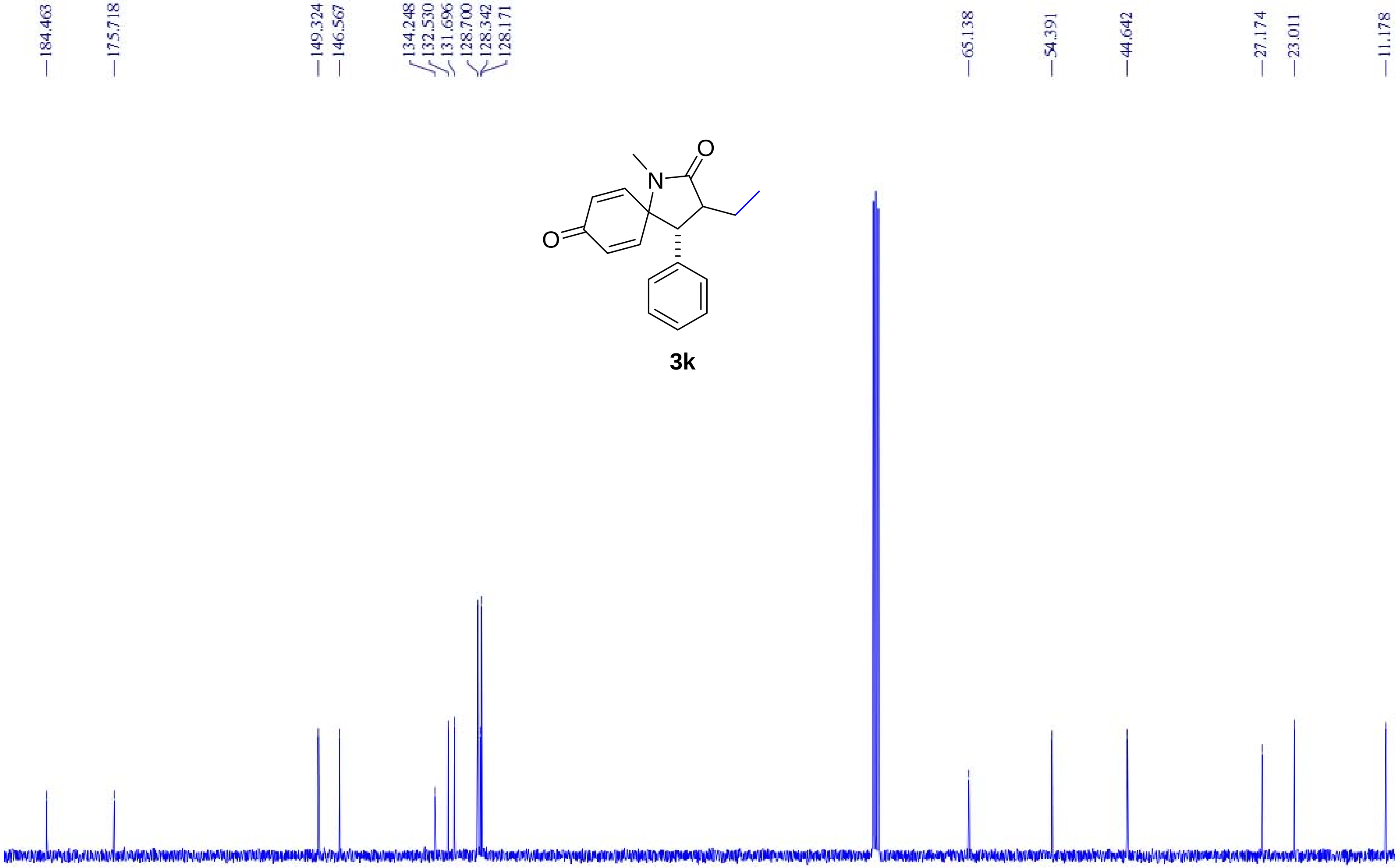
184.463
175.718
149.324
146.567
134.248
132.530
131.696
128.700
128.342
128.171
65.138
54.391
44.642
27.174
23.011
11.178
O
N
O
3k

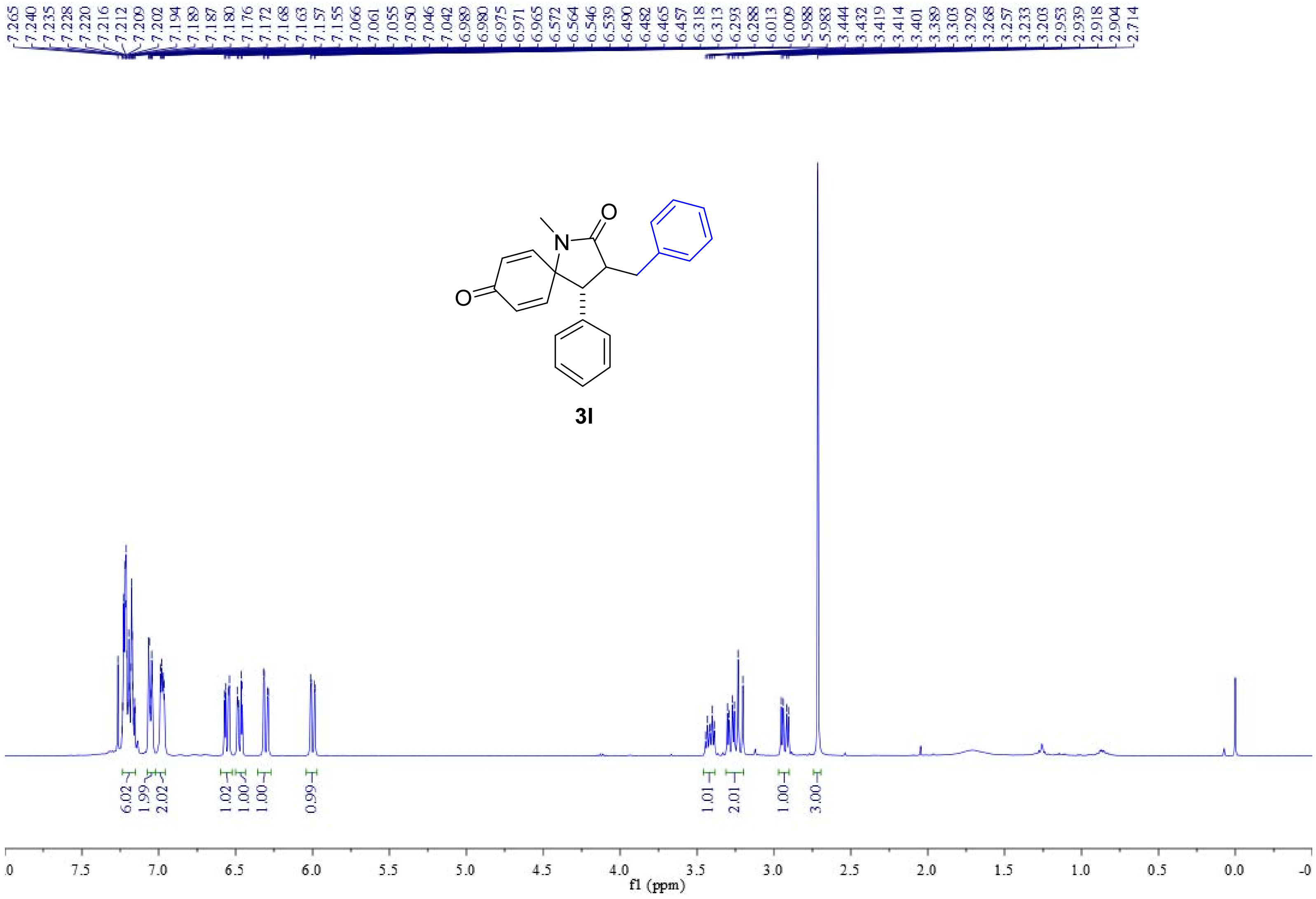

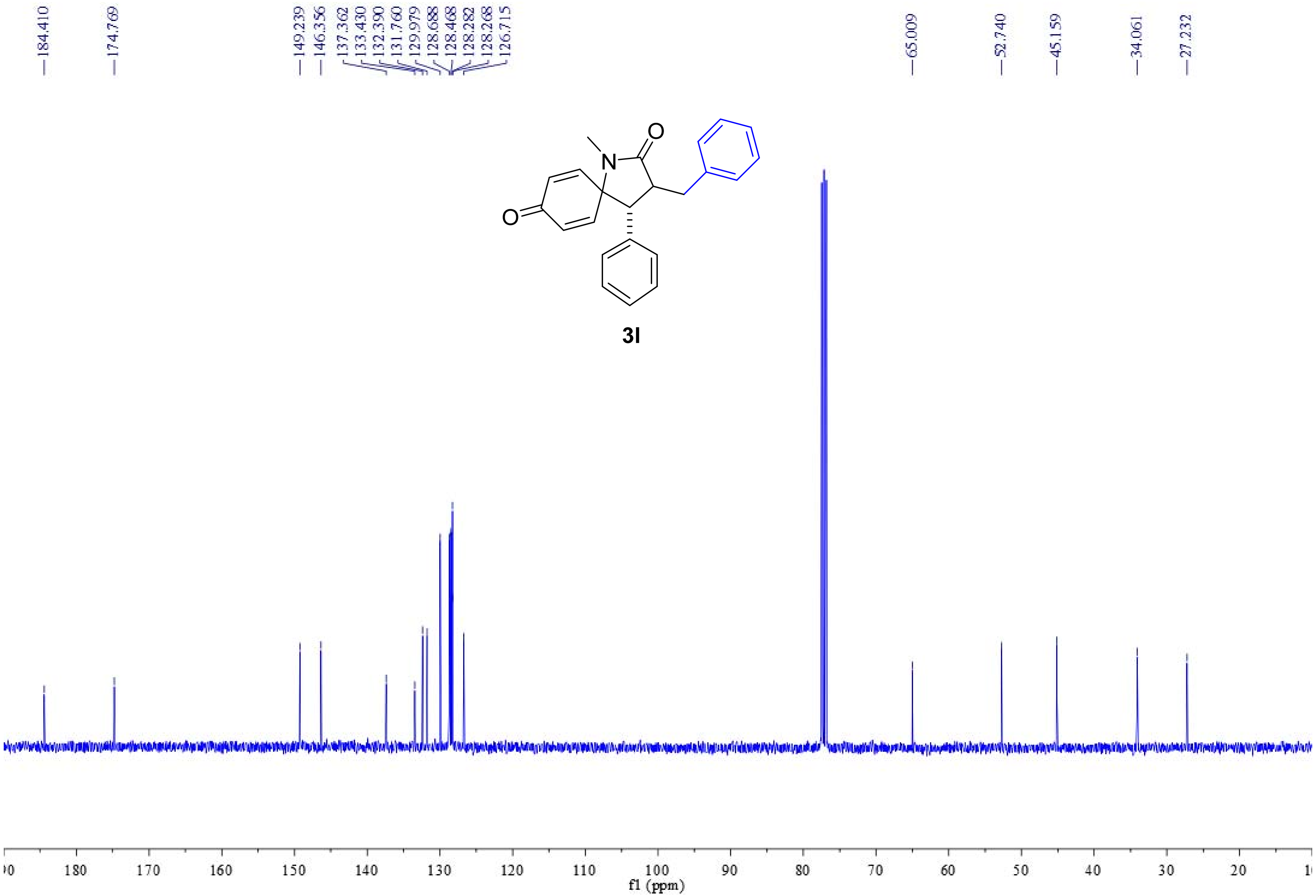
184.410
174.769
149.239
146.356
137.362
133.430
132.390
131.760
129.979
128.688
128.468
128.282
128.268
126.715
65.009
52.740
45.159
34.061
27.232
3l
180
170
160
150
140
130
120
110
100
90
80
70
60
50
40
30
20
f1 (ppm)

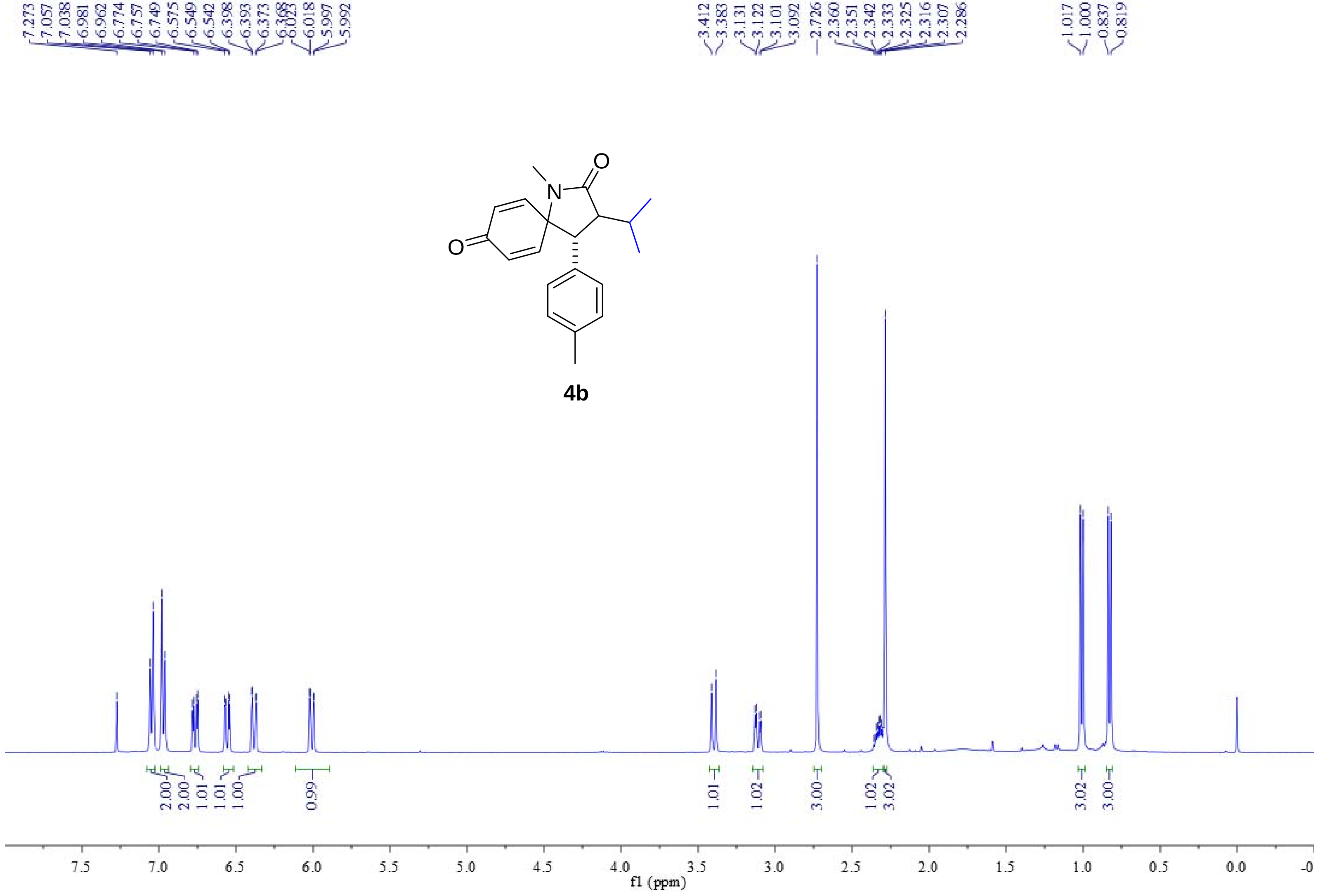

4b
f1 (ppm)

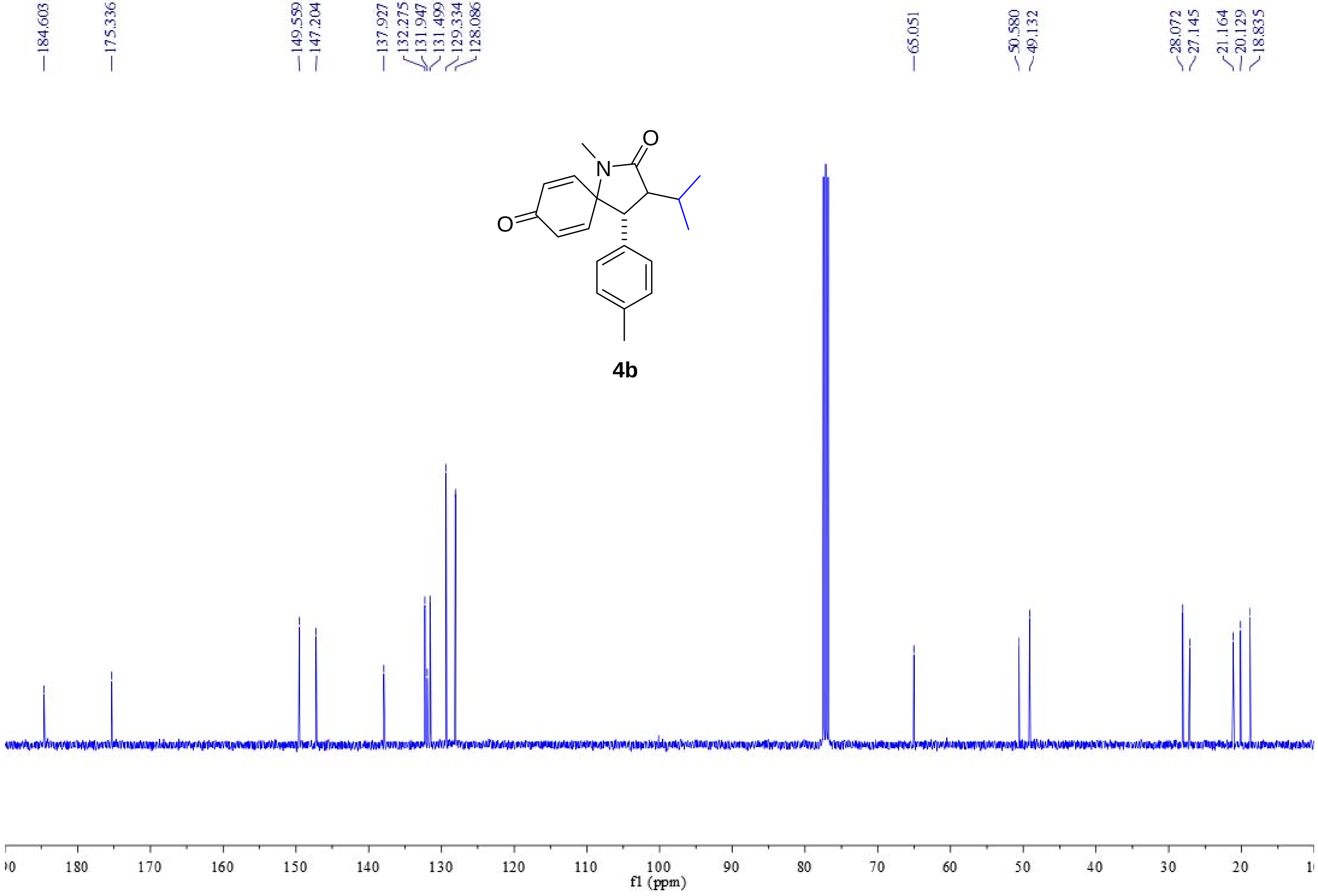
184.603
175.336
149.559
147.204
137.927
132.275
131.947
131.499
129.334
128.086
65.051
50.580
49.132
28.072
27.145
21.164
20.129
18.835
O
N
O
4b
f1 (ppm)

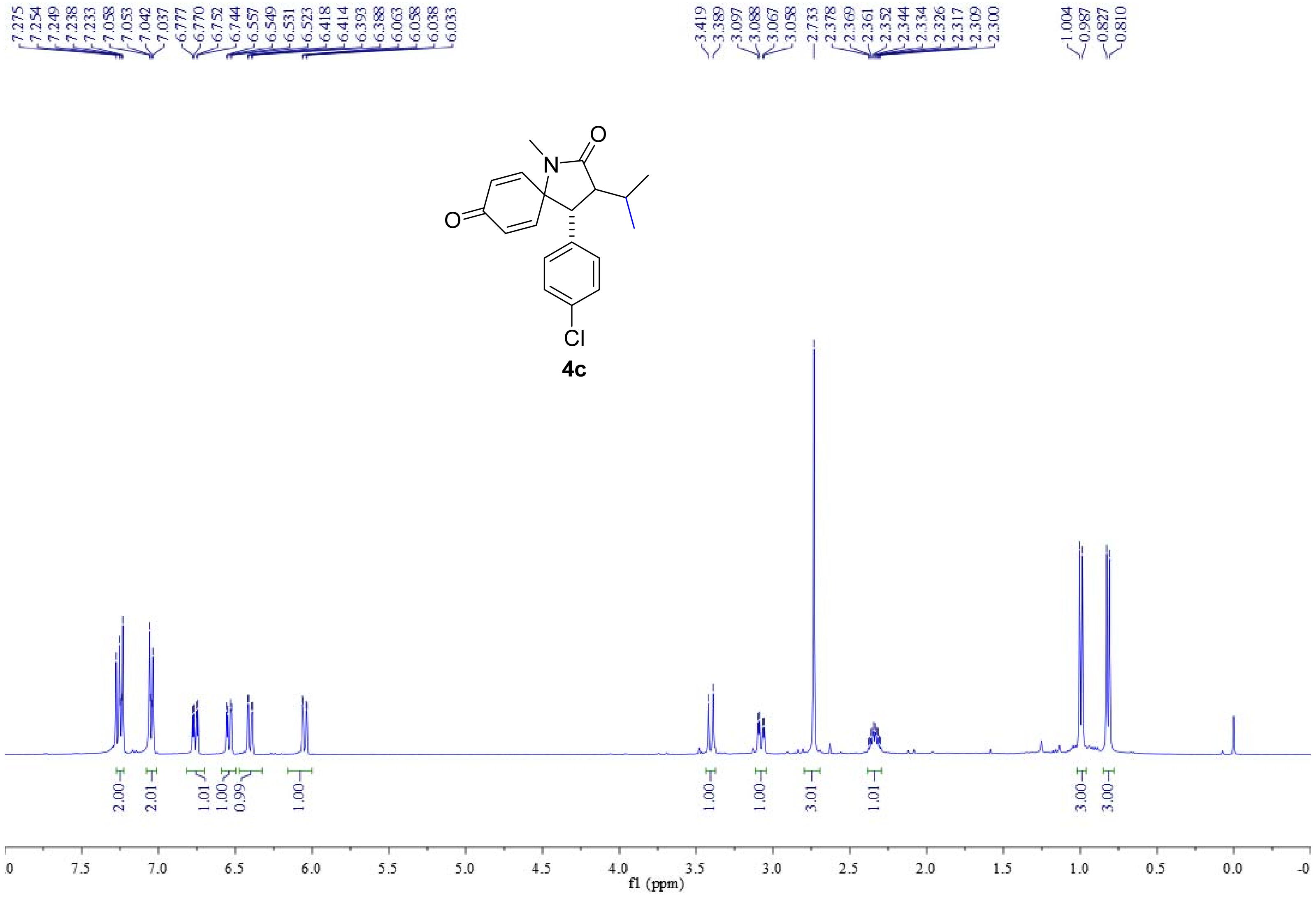

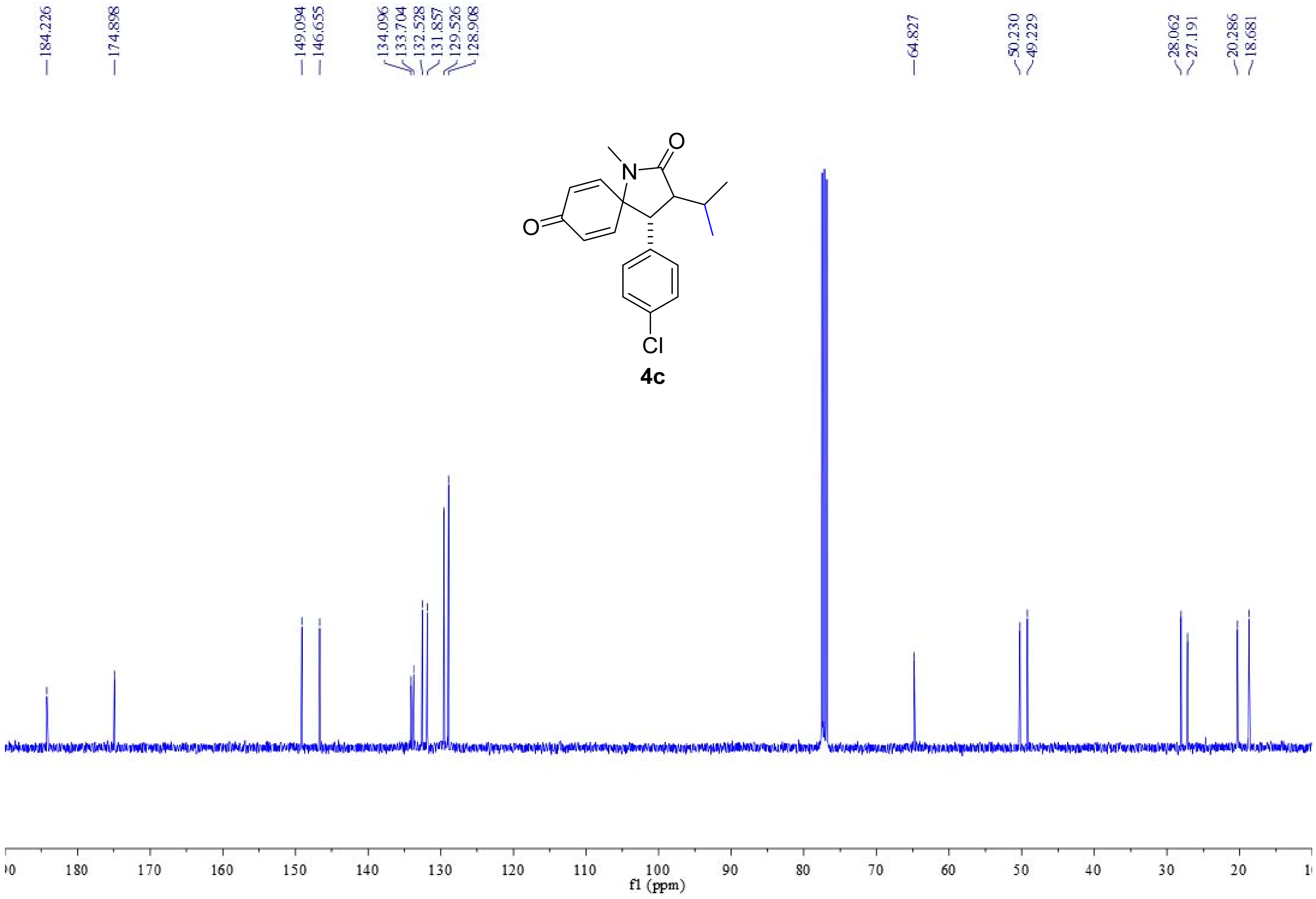

184.226
174.898
149.094
146.655
134.096
133.704
132.528
131.857
129.526
128.908
64.827
50.230
49.229
28.062
27.191
20.286
18.681
O
N
O
Cl
4c
180
170
160
150
140
130
120
110
100
90
80
70
60
50
40
30
20
f1 (ppm)

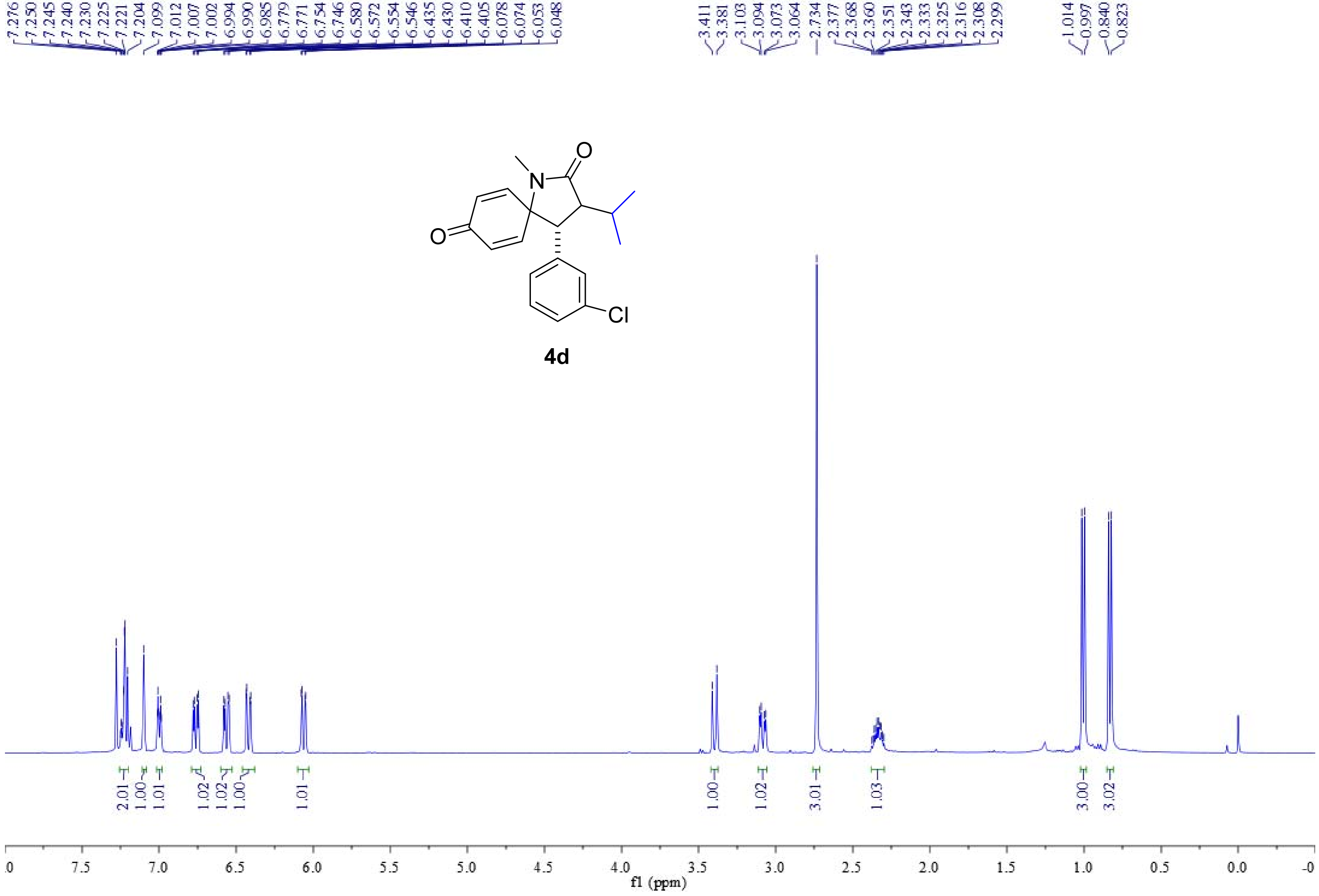

7.276
7.250
7.245
7.240
7.230
7.225
7.221
7.204
7.099
7.012
7.007
7.002
6.994
6.990
6.985
6.779
6.771
6.754
6.746
6.580
6.572
6.554
6.546
6.435
6.430
6.410
6.405
6.078
6.074
6.053
6.048
3.411
3.381
3.103
3.094
3.073
3.064
2.734
2.377
2.368
2.360
2.351
2.343
2.333
2.325
2.316
2.308
2.299
1.014
0.997
0.840
0.823
N
O
O
Cl
4d
2.01
1.00
1.01
1.02
1.02
1.00
1.01
1.00
1.02
3.01
1.03
3.00
3.02
7.5
7.0
6.5
6.0
5.5
5.0
4.5
4.0
3.5
3.0
2.5
2.0
1.5
1.0
0.5
0.0
f1 (ppm)

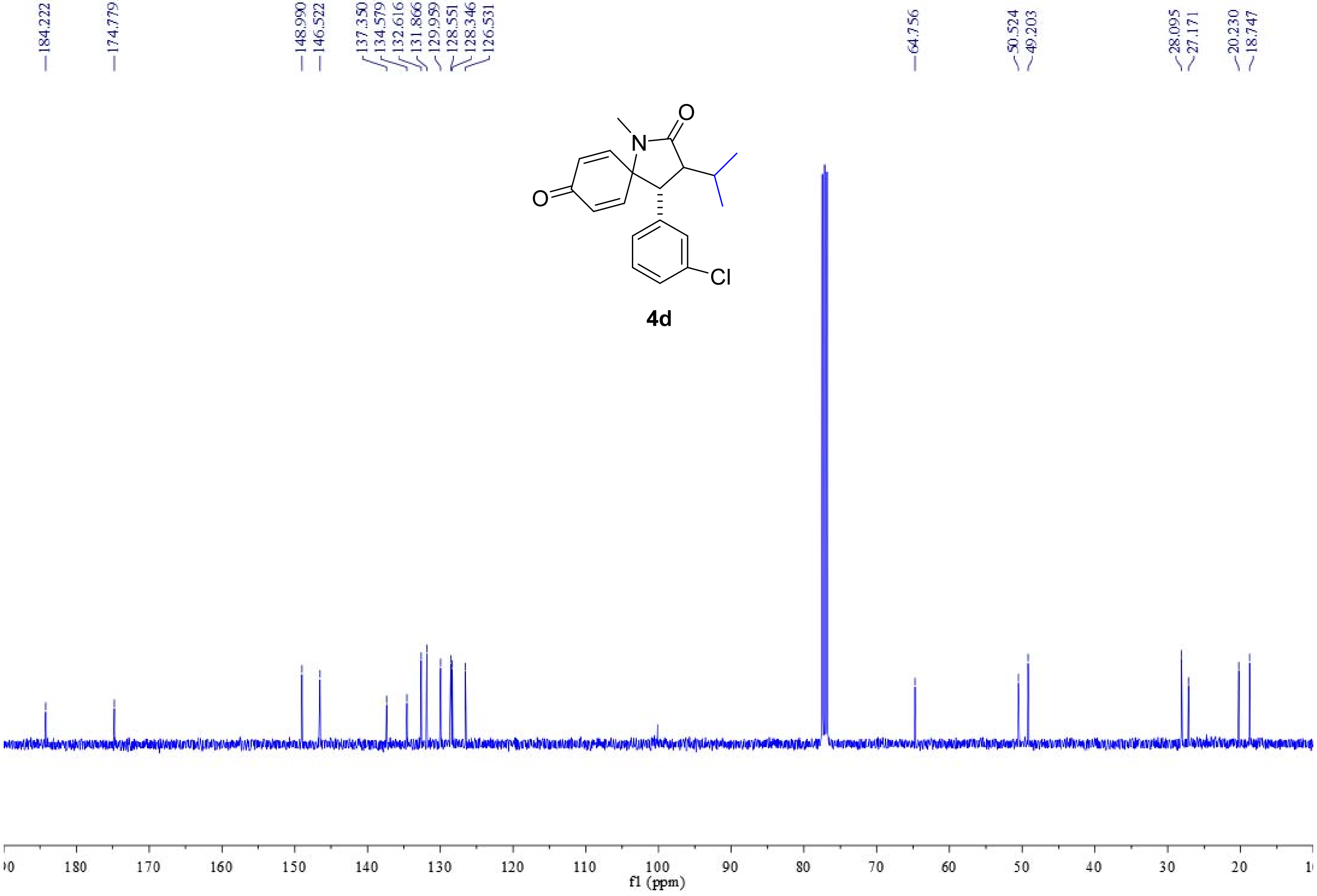
184.222
174.779
148.990
146.522
137.350
134.579
132.616
131.866
129.959
128.551
128.346
126.531
64.756
50.524
49.203
28.095
27.171
20.230
18.747
O
N
O
Cl
4d
180
170
160
150
140
130
120
110
100
90
80
70
60
50
40
30
20
f1 (ppm)

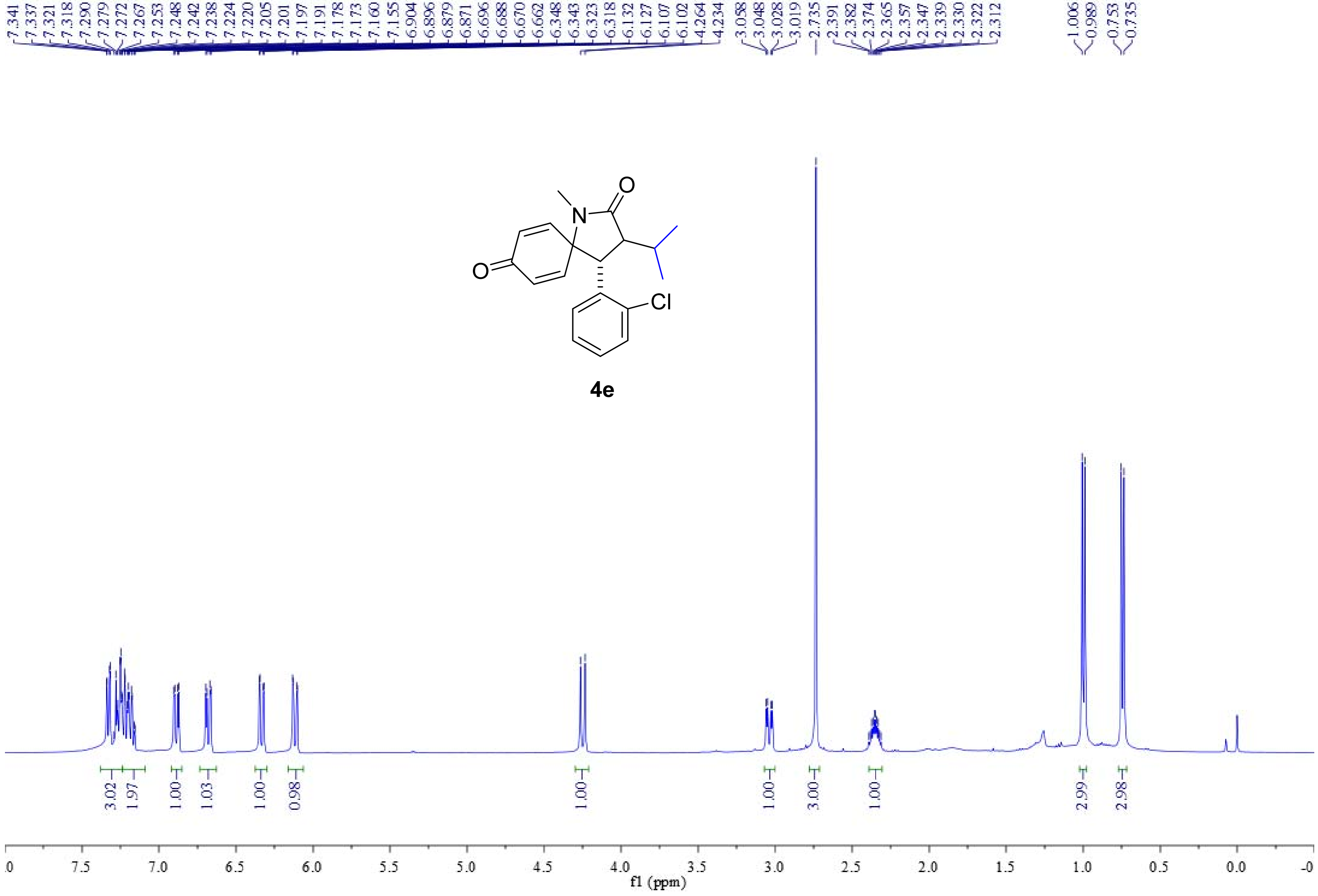
7.341 7.337 7.321 7.318 7.290 7.279 7.272 7.267 7.253 7.248 7.242 7.238 7.224 7.220 7.205 7.201 7.197 7.191 7.178 7.173 7.160 7.155 6.904 6.896 6.879 6.871 6.696 6.688 6.670 6.662 6.348 6.343 6.323 6.318 6.132 6.127 6.107 6.102 4.264 4.234
3.058 3.048 3.028 3.019 2.735 2.391 2.382 2.374 2.365 2.357 2.347 2.339 2.330 2.322 2.312
1.006 0.989 0.753 0.735
O
N
O
Cl
4e
3.02 1.97 1.00 1.03 1.00 0.98 1.00 1.00 3.00 1.00 2.99 2.98
f1 (ppm)

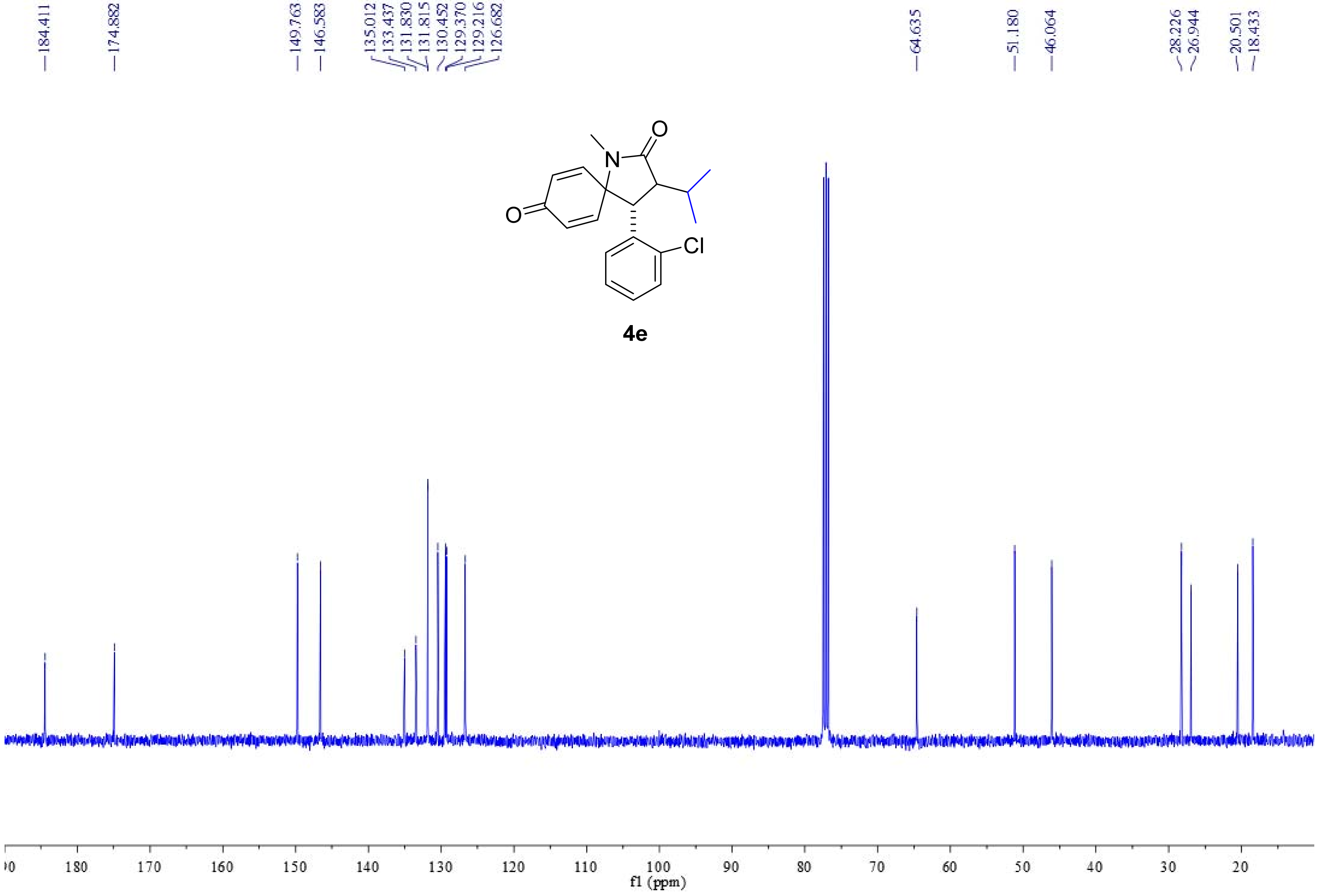
184.411
174.882
149.763
146.583
135.012
133.437
131.830
131.815
130.452
129.370
129.216
126.682
64.635
51.180
46.064
28.226
26.944
20.501
18.433
N
O
O
Cl
4e
180 170 160 150 140 130 120 110 100 90 80 70 60 50 40 30 20
f1 (ppm)

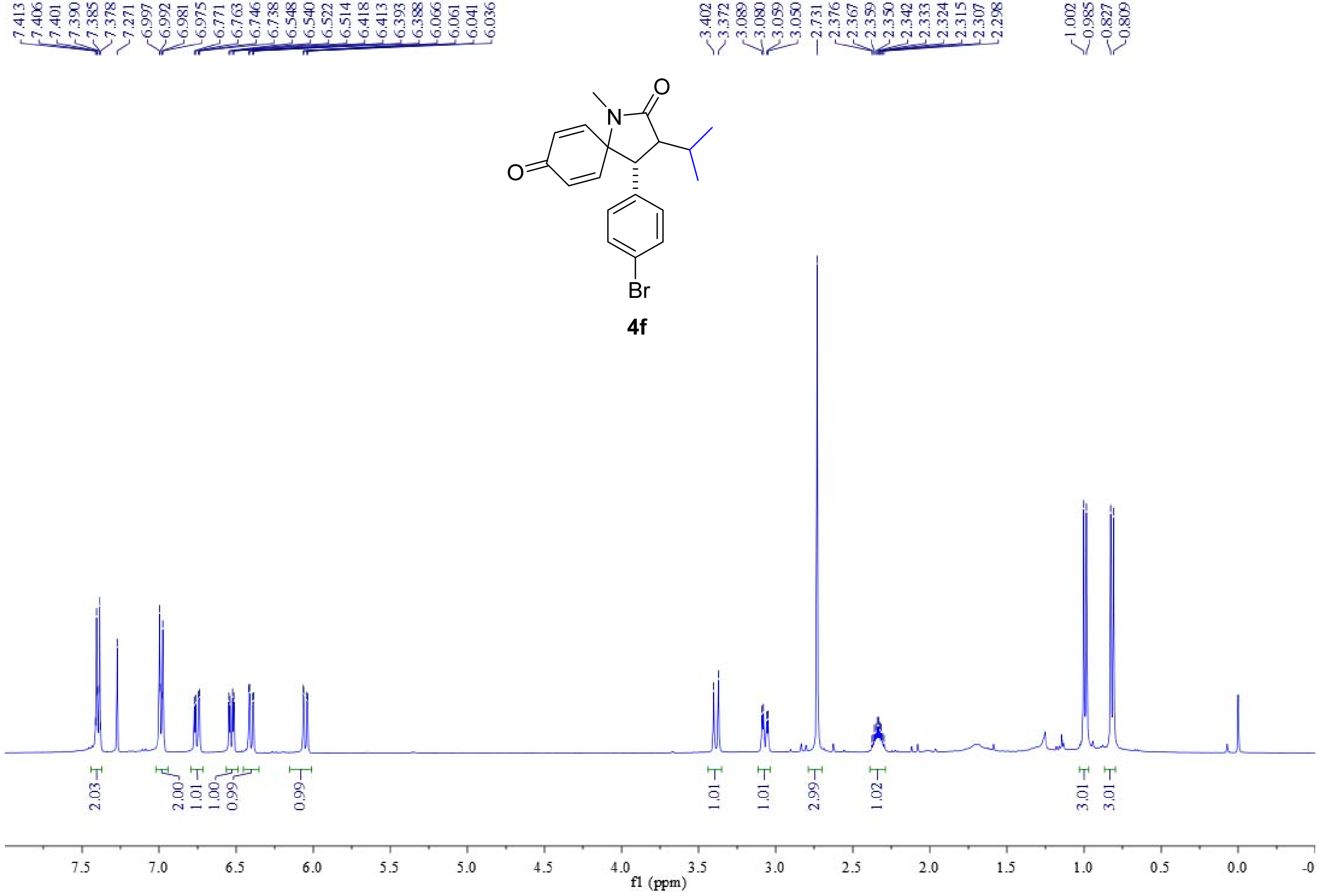

7.413
7.406
7.401
7.390
7.385
7.378
7.271
6.997
6.992
6.981
6.975
6.771
6.763
6.746
6.738
6.548
6.540
6.522
6.514
6.418
6.413
6.393
6.388
6.066
6.061
6.041
6.036
3.402
3.372
3.089
3.080
3.059
3.050
2.731
2.376
2.367
2.359
2.350
2.342
2.333
2.324
2.315
2.307
2.298
1.002
0.985
0.827
0.809
O
N
O
Br
4f
2.03
2.00
1.01
1.00
0.99
0.99
1.01
1.01
2.99
1.02
3.01
3.01
f1 (ppm)

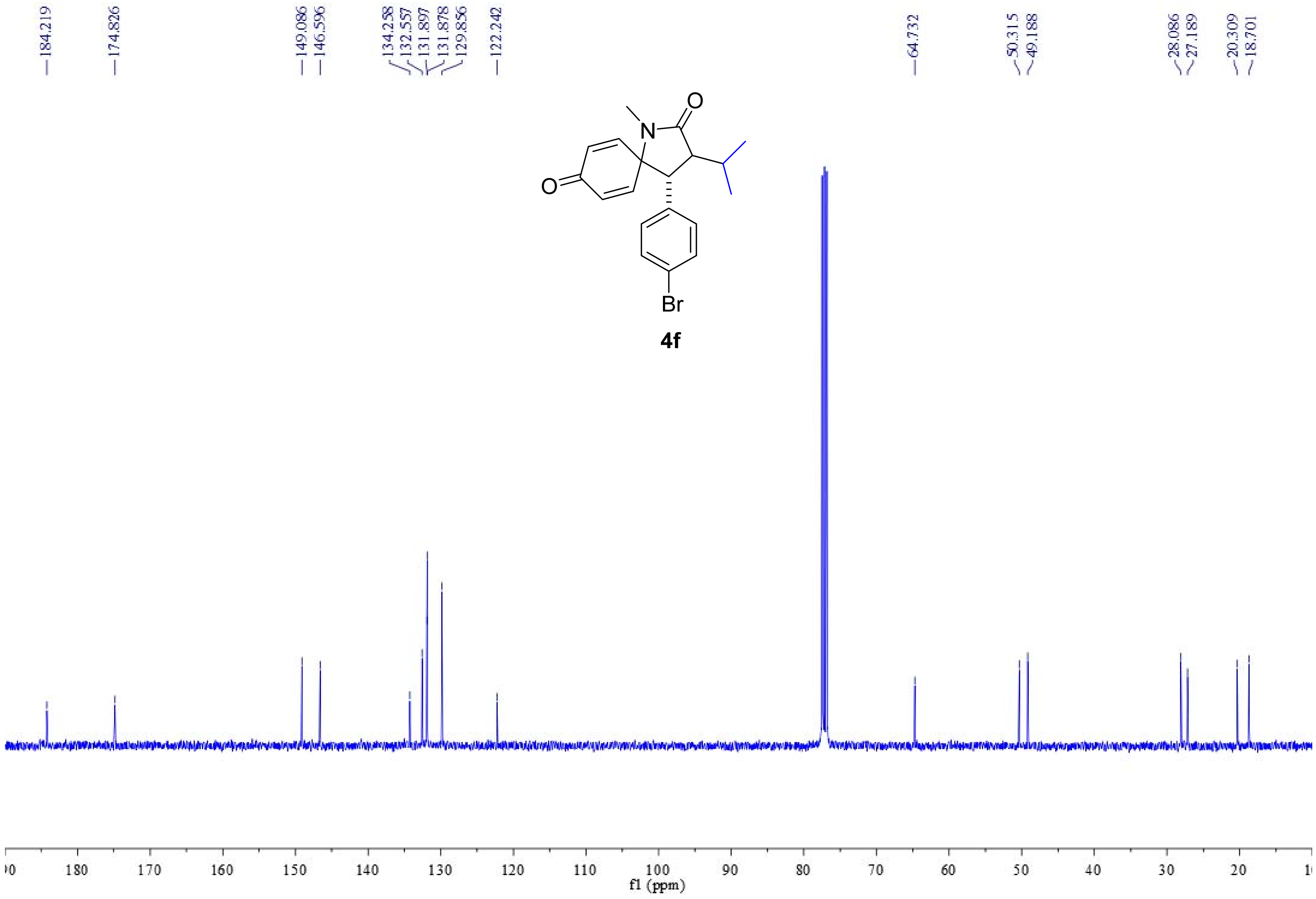

184.219
174.826
149.086
146.596
134.258
132.557
131.897
131.878
129.856
122.242
64.732
50.315
49.188
28.086
27.189
20.309
18.701
O
N
O
Br
4f
180
170
160
150
140
130
120
110
100
90
80
70
60
50
40
30
20
f1 (ppm)

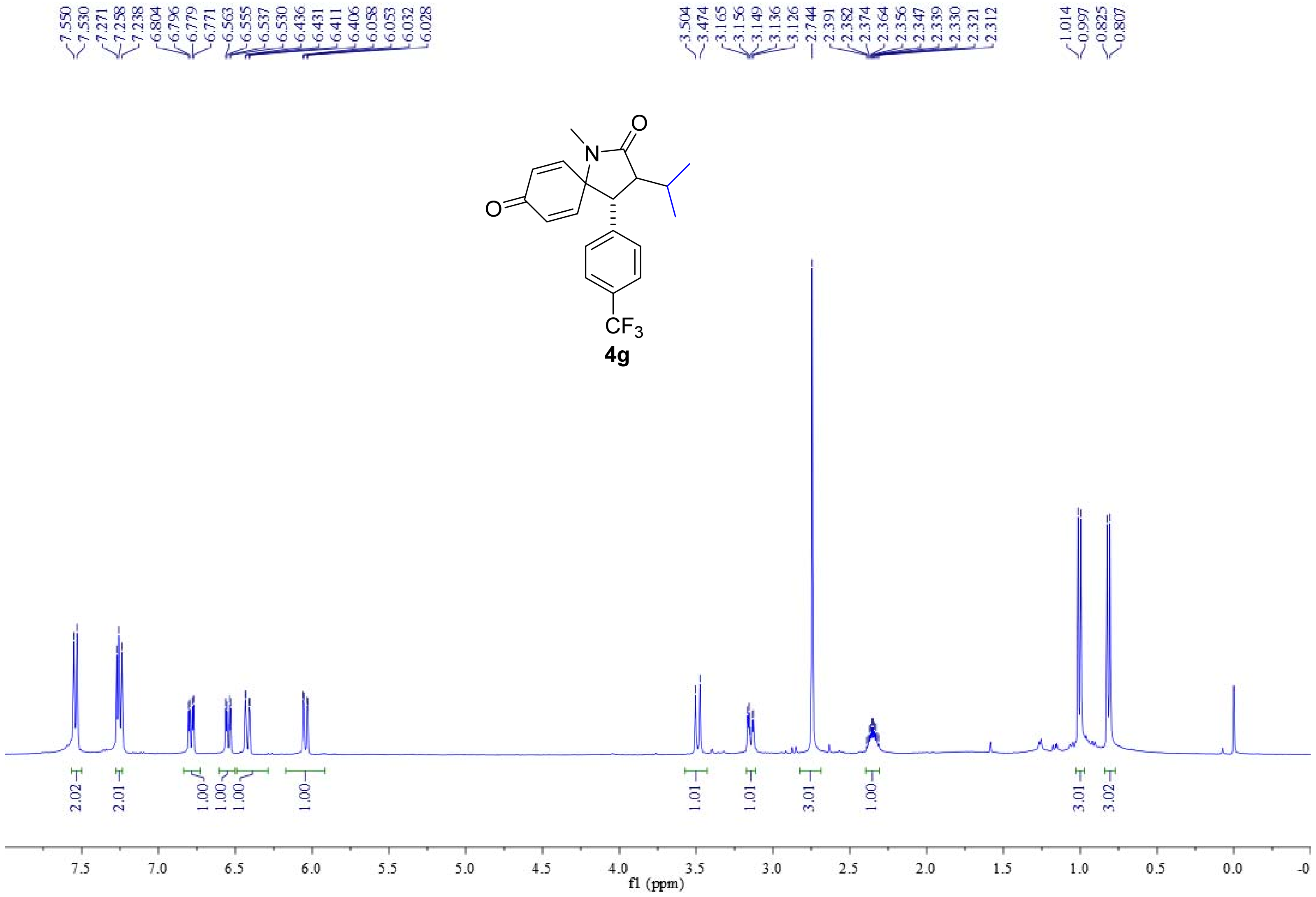
7.550
7.530
7.271
7.258
7.238
6.804
6.796
6.779
6.771
6.563
6.555
6.537
6.530
6.436
6.431
6.411
6.406
6.058
6.053
6.032
6.028
3.504
3.474
3.165
3.156
3.149
3.136
3.126
2.744
2.391
2.382
2.374
2.364
2.356
2.347
2.339
2.330
2.321
2.312
1.014
0.997
0.825
0.807
N
O
O
CF3
4g
2.02
2.01
1.00
1.00
1.00
1.00
1.01
1.01
3.01
1.00
3.01
3.02
7.5
7.0
6.5
6.0
5.5
5.0
4.5
4.0
3.5
3.0
2.5
2.0
1.5
1.0
0.5
0.0
-0
f1 (ppm)

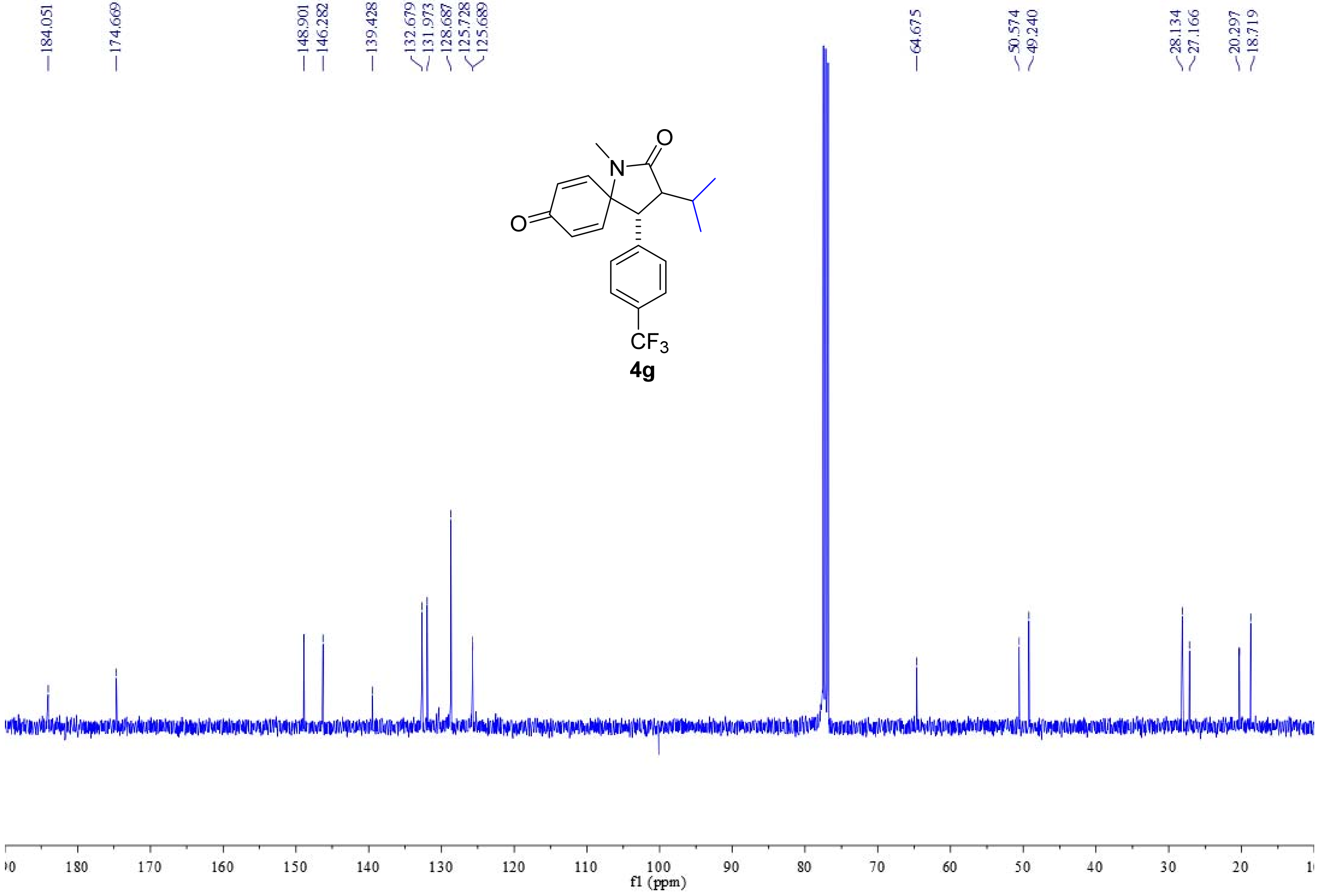

184.051
174.669
148.901
146.282
139.428
132.679
131.973
128.687
125.728
125.689
64.675
50.574
49.240
28.134
27.166
20.297
18.719
O
N
O
CF3
4g
180
170
160
150
140
130
120
110
100
90
80
70
60
50
40
30
20
f1 (ppm)

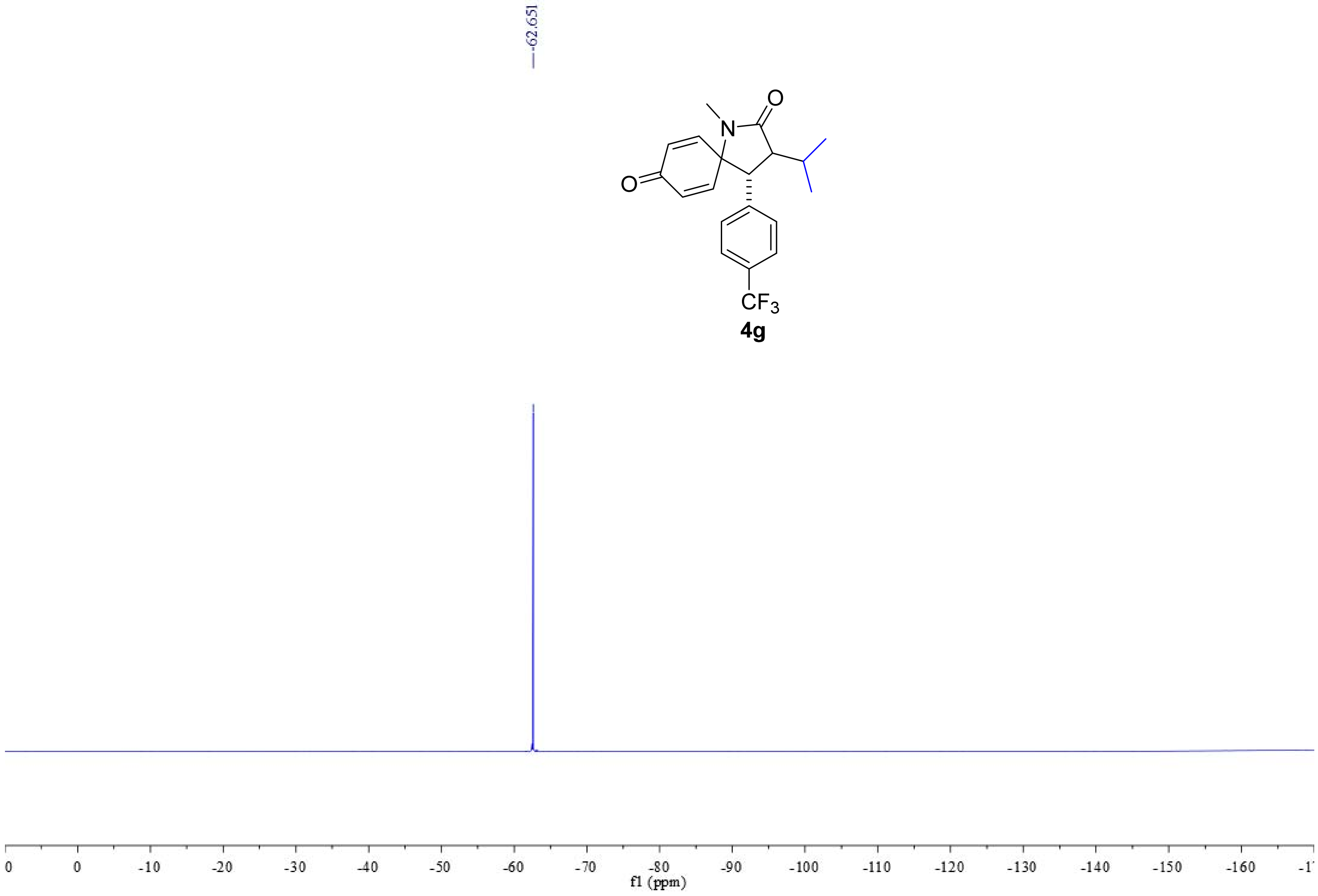
-62.651
O
N
O
CF3
4g
0 0 -10 -20 -30 -40 -50 -60 -70 -80 -90 -100 -110 -120 -130 -140 -150 -160
f1 (ppm)

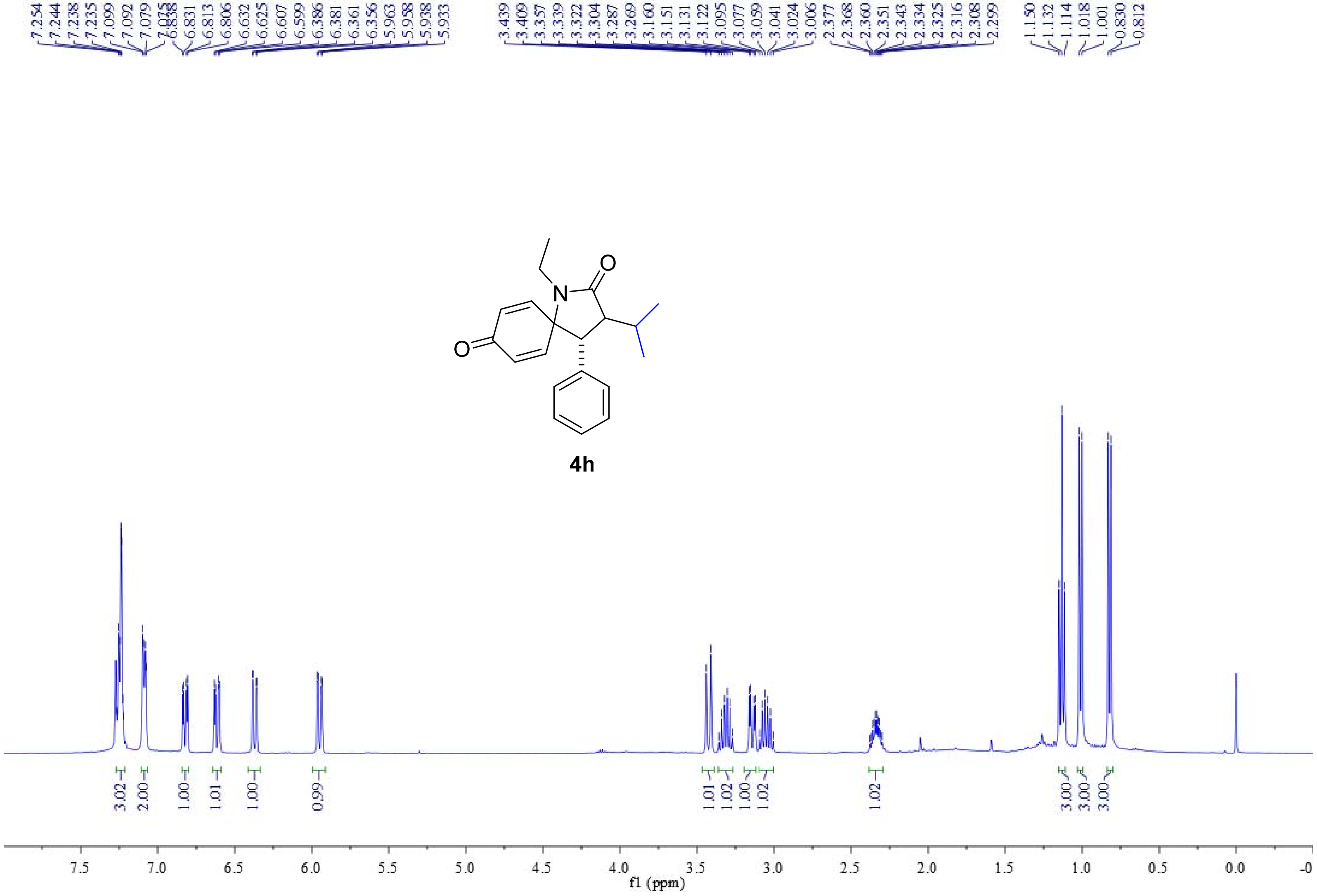
7.254
7.244
7.238
7.235
7.099
7.092
7.079
7.075
6.838
6.831
6.813
6.806
6.632
6.625
6.607
6.599
6.386
6.381
6.361
6.356
5.963
5.958
5.938
5.933
3.439
3.409
3.357
3.339
3.322
3.304
3.287
3.269
3.160
3.151
3.131
3.122
3.095
3.077
3.059
3.041
3.024
3.006
2.377
2.368
2.360
2.351
2.343
2.334
2.325
2.316
2.308
2.299
1.150
1.132
1.114
1.018
1.001
0.830
0.812
N
O
O
4h
3.02
2.00
1.00
1.01
1.00
0.99
1.01
1.02
1.00
1.02
1.02
3.00
3.00
3.00
7.5
7.0
6.5
6.0
5.5
5.0
4.5
4.0
3.5
3.0
2.5
2.0
1.5
1.0
0.5
0.0
-0
f1 (ppm)

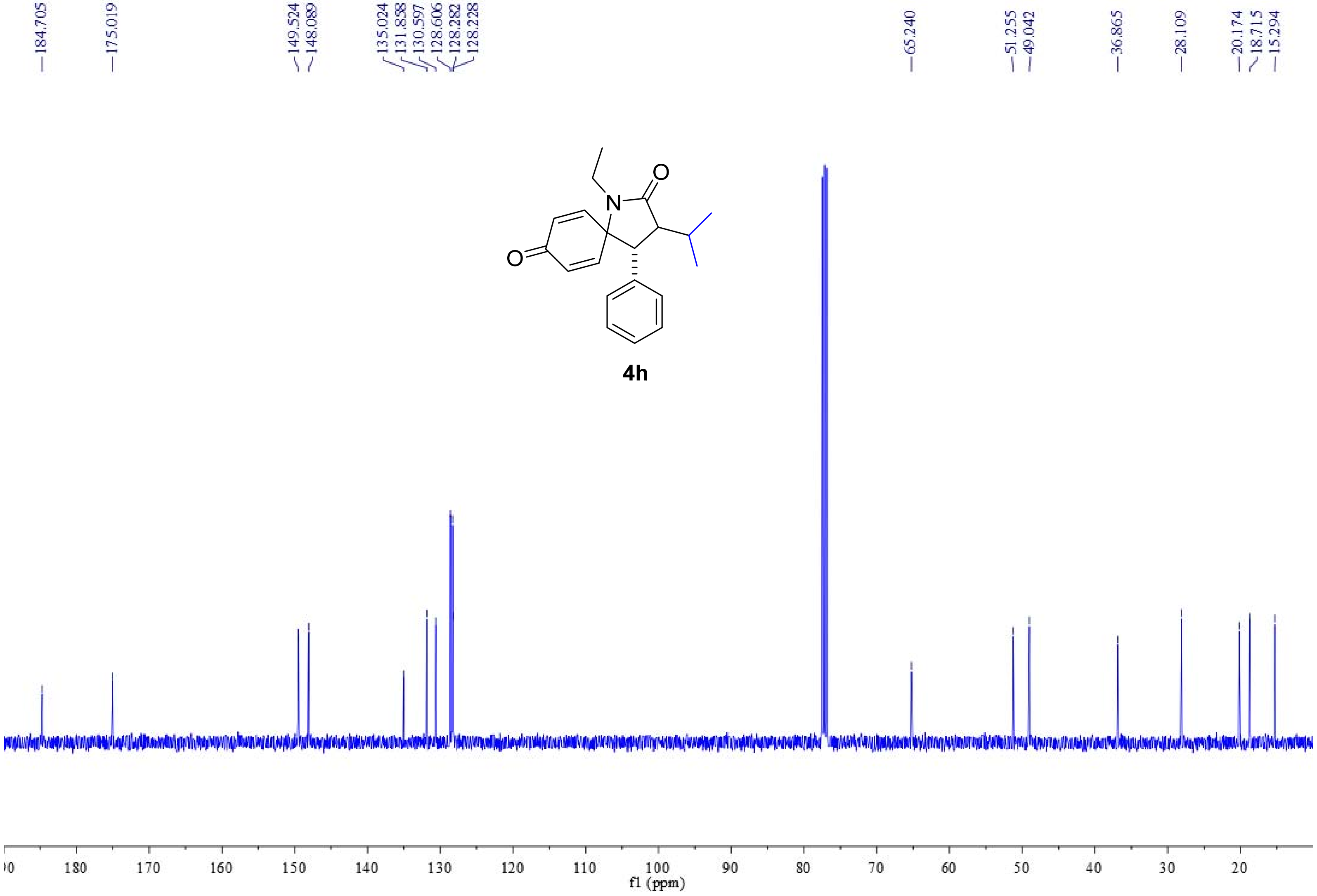

184.705
175.019
149.524
148.089
135.024
131.858
130.597
128.606
128.282
128.228
65.240
51.255
49.042
36.865
28.109
20.174
18.715
15.294
O
N
O
4h
180 170 160 150 140 130 120 110 100 90 80 70 60 50 40 30 20
f1 (ppm)

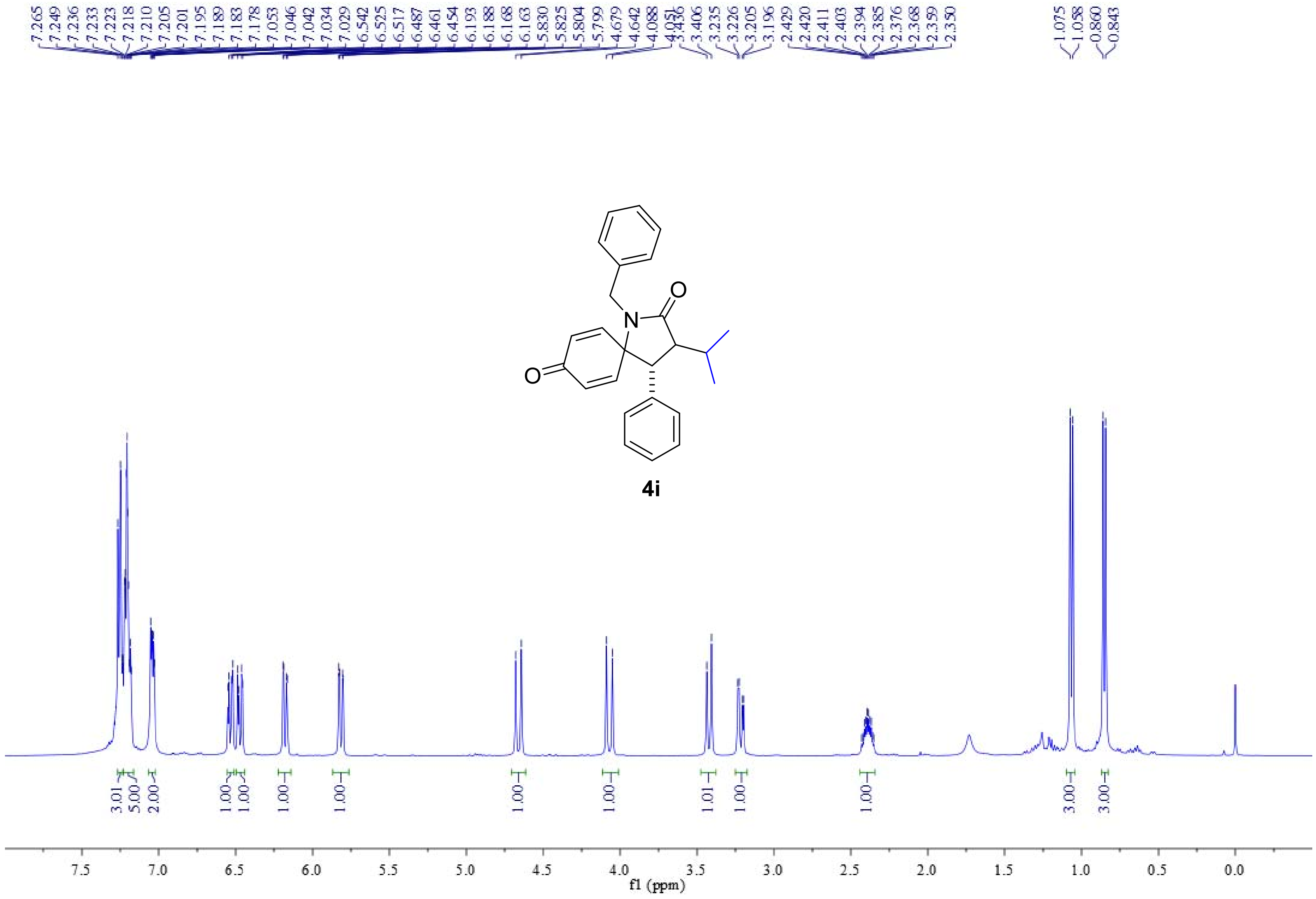

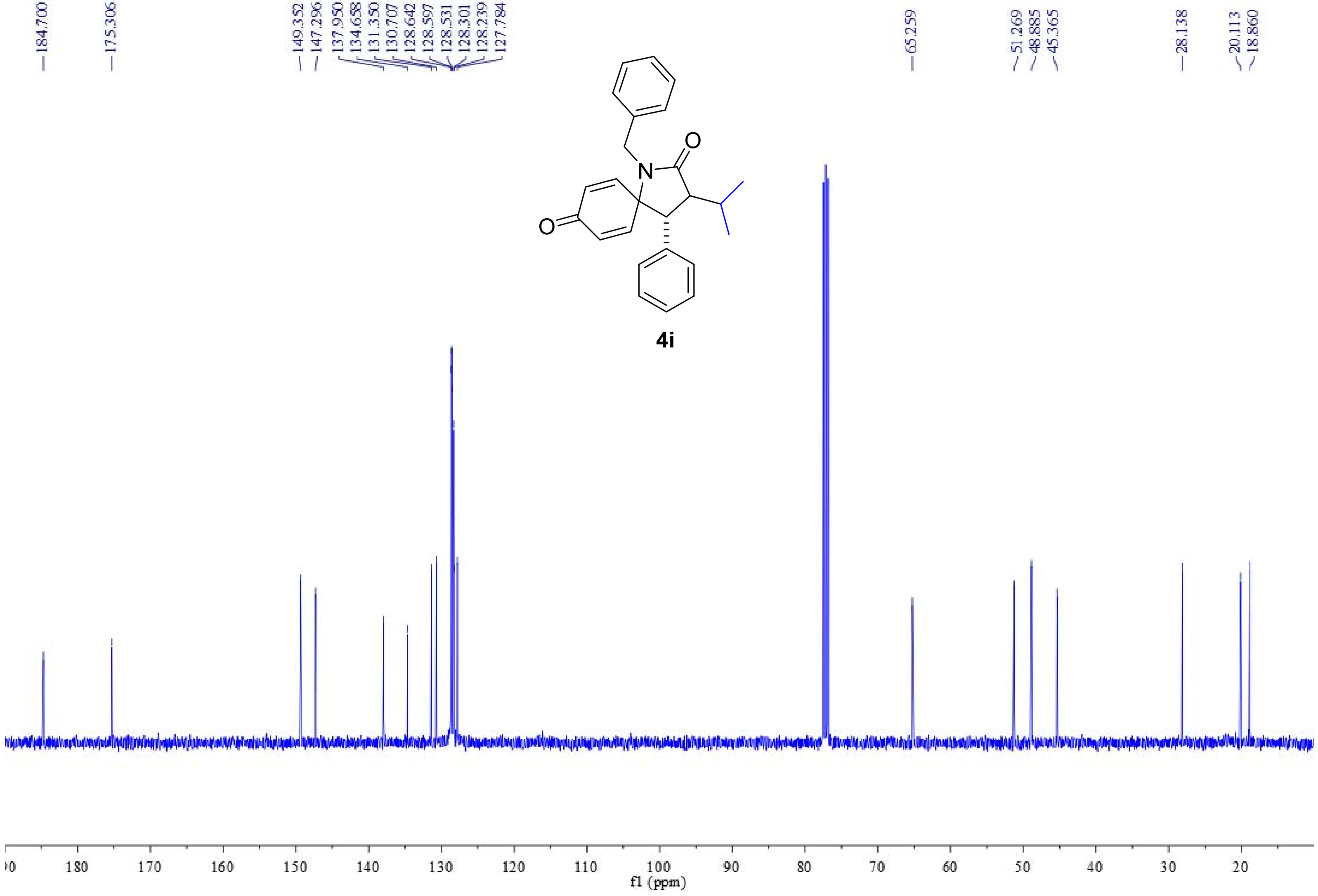
184.700
175.306
149.352
147.296
137.950
134.658
131.350
130.707
128.642
128.597
128.531
128.301
128.239
127.784
65.259
51.269
48.885
45.365
28.138
20.113
18.860
4i
f1 (ppm)

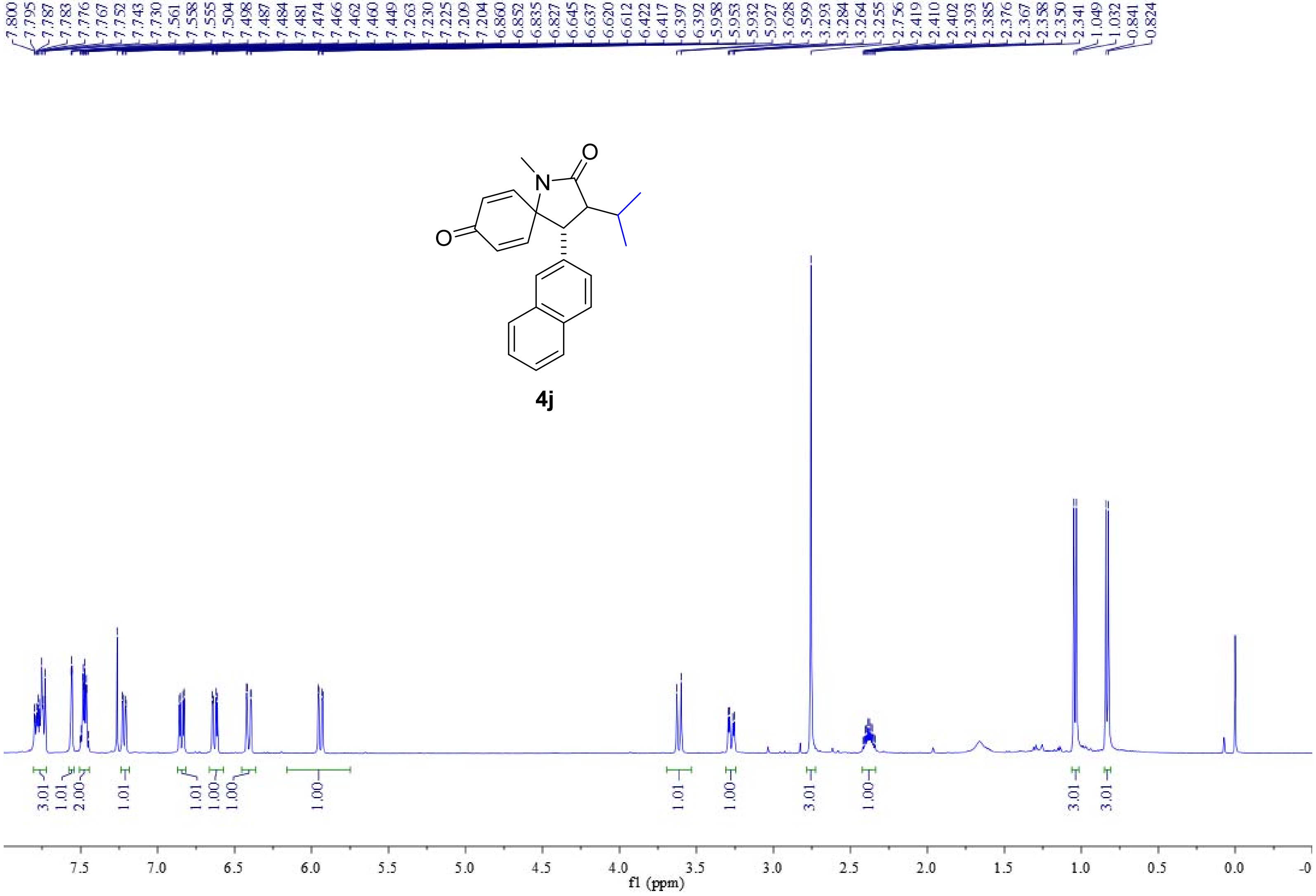
7.800 7.795 7.787 7.783 7.776 7.767 7.752 7.743 7.730 7.561 7.558 7.555 7.504 7.498 7.487 7.484 7.481 7.474 7.466 7.462 7.460 7.449 7.263 7.230 7.225 7.209 7.204 6.860 6.852 6.835 6.827 6.645 6.637 6.620 6.612 6.422 6.417 6.397 6.392 5.958 5.953 5.932 5.927 3.628 3.599 3.293 3.284 3.264 3.255 2.756 2.419 2.410 2.402 2.393 2.385 2.376 2.367 2.358 2.350 2.341 1.049 1.032 0.841 0.824
O
N
O
4j
3.01 1.01 2.00 1.01 1.01 1.00 1.00 1.00 1.01 1.00 3.01 1.00 3.01 3.01
7.5 7.0 6.5 6.0 5.5 5.0 4.5 4.0 3.5 3.0 2.5 2.0 1.5 1.0 0.5 0.0 -0
f1 (ppm)

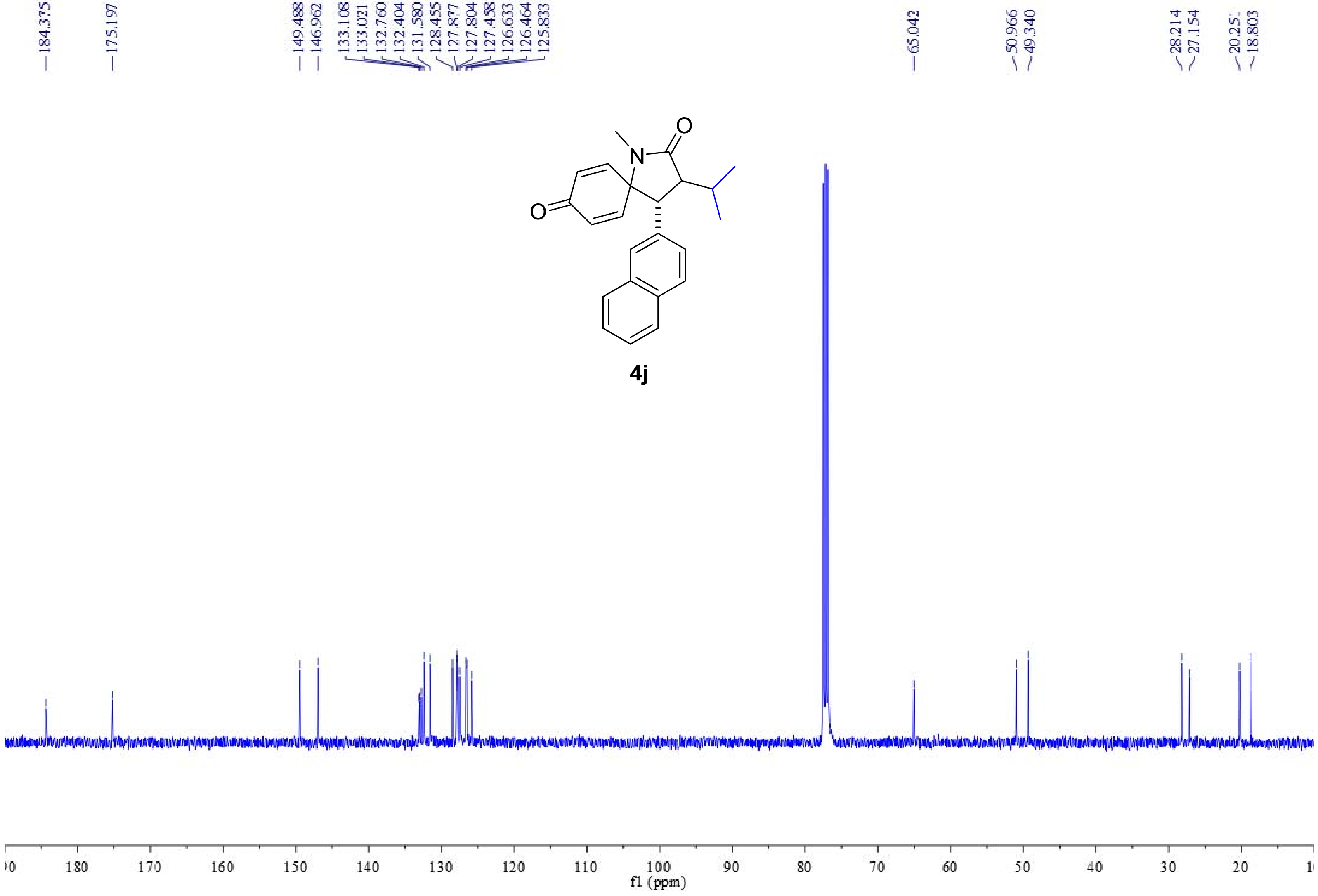
184.375
175.197
149.488
146.962
133.108
133.021
132.760
132.404
131.580
128.455
127.877
127.804
127.458
126.633
126.464
125.833
65.042
50.966
49.340
28.214
27.154
20.251
18.803
N
O
O
4j
180
170
160
150
140
130
120
110
100
90
80
70
60
50
40
30
20
f1 (ppm)

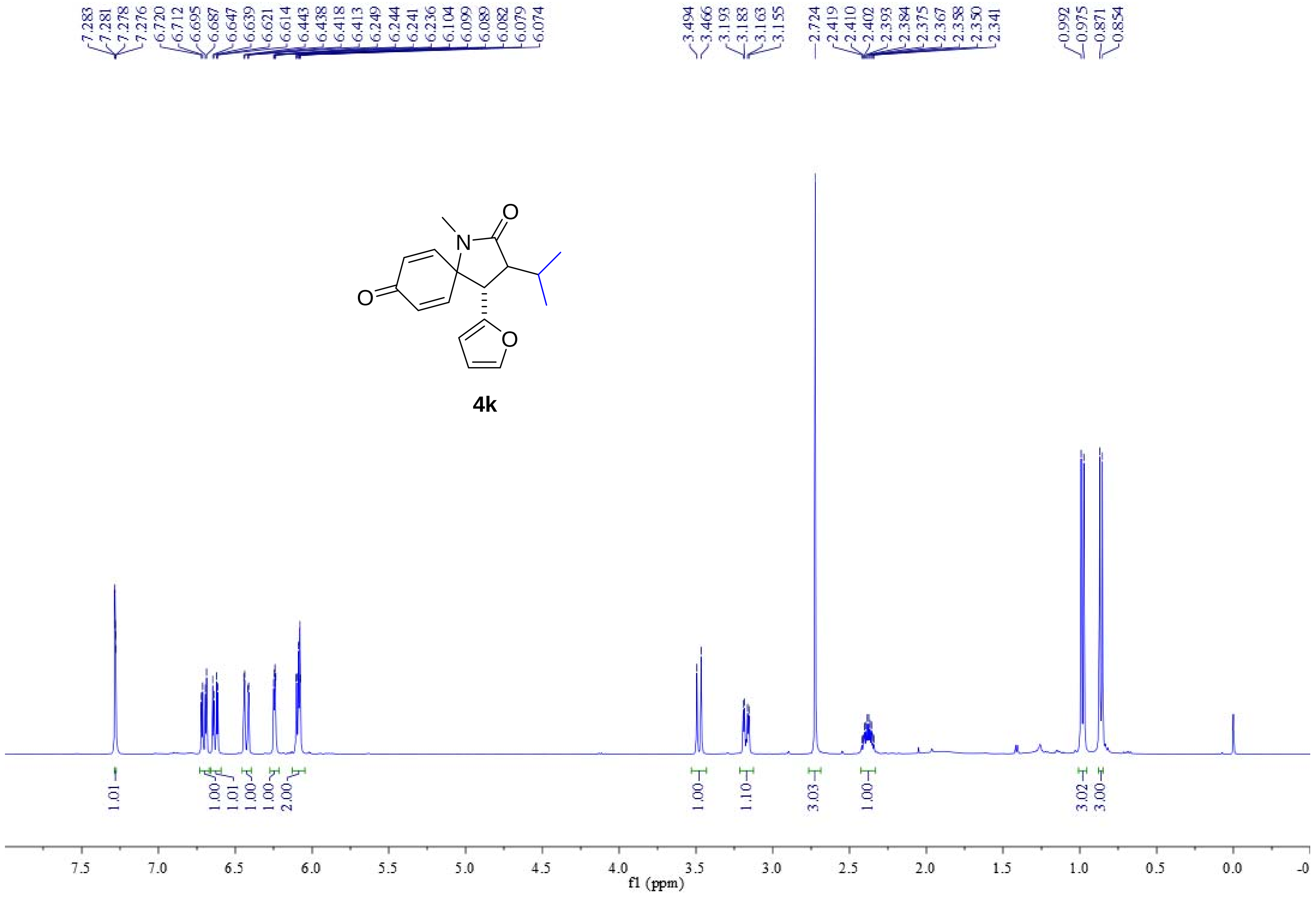

N
O
O
O
4k
f1 (ppm)

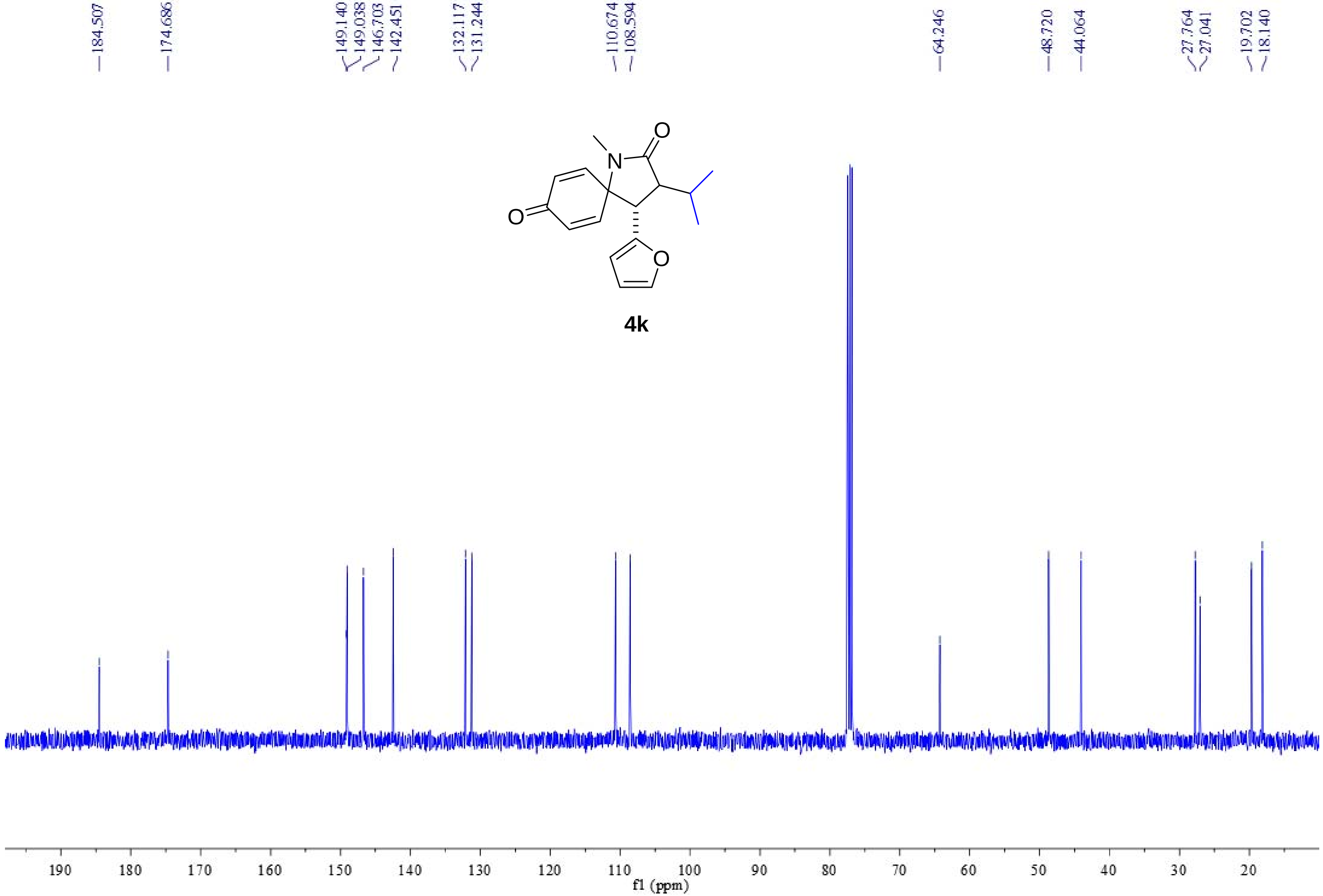

184.507
174.686
149.140
149.038
146.703
142.451
132.117
131.244
110.674
108.594
64.246
48.720
44.064
27.764
27.041
19.702
18.140
O
N
O
O
4k
190 180 170 160 150 140 130 120 110 100 90 80 70 60 50 40 30 20
f1 (ppm)

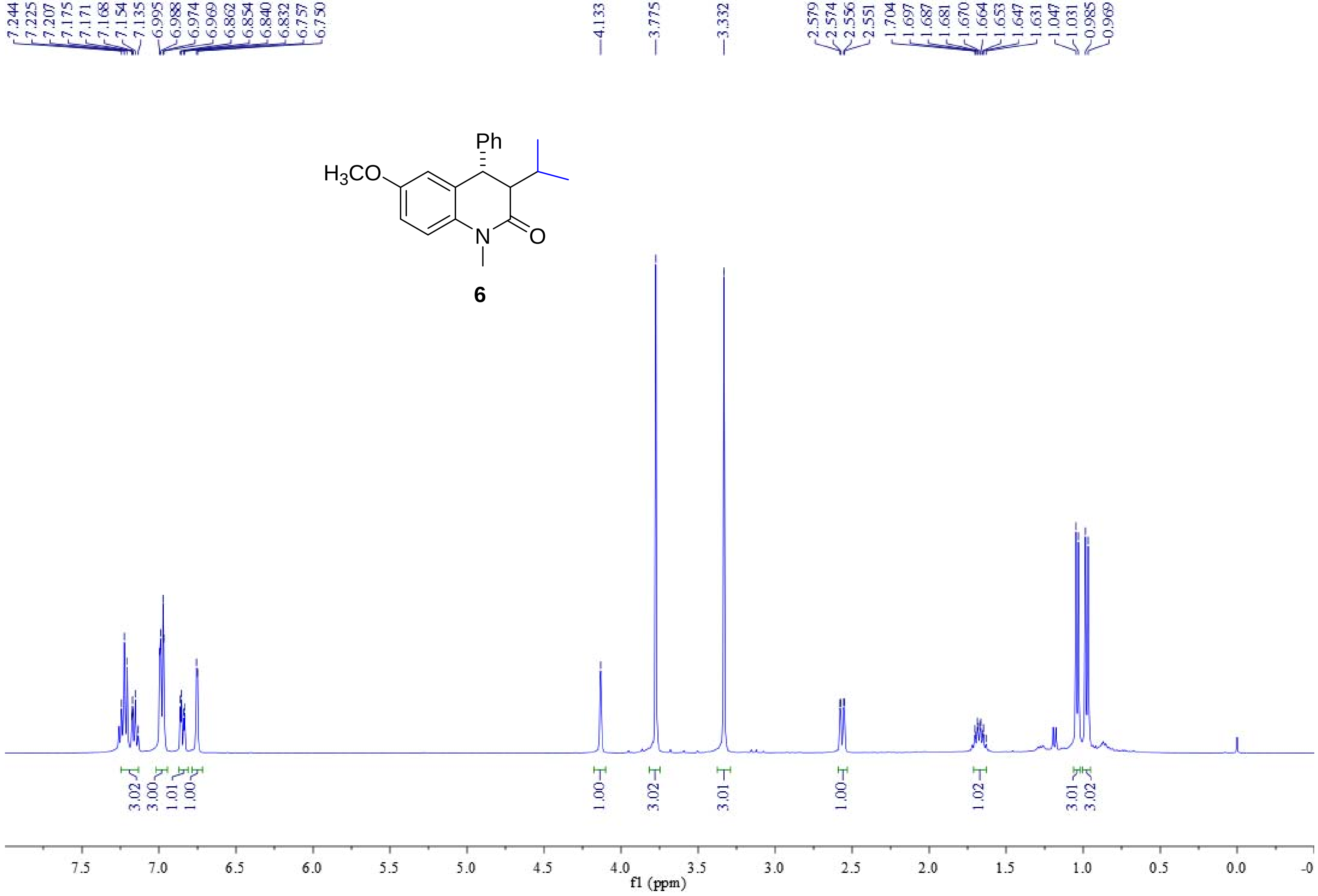
7.244
7.225
7.207
7.175
7.171
7.168
7.154
7.135
6.995
6.988
6.974
6.969
6.862
6.854
6.840
6.832
6.757
6.750
4.133
3.775
3.332
2.579
2.574
2.556
2.551
1.704
1.697
1.687
1.681
1.670
1.664
1.653
1.647
1.631
1.047
1.031
0.985
0.969
Ph
H3CO
N
O
3.02
3.00
1.01
1.00
1.00
3.02
3.01
1.00
1.02
3.01
3.02
7.5
7.0
6.5
6.0
5.5
5.0
4.5
4.0
3.5
3.0
2.5
2.0
1.5
1.0
0.5
0.0
-0
f1 (ppm)

6

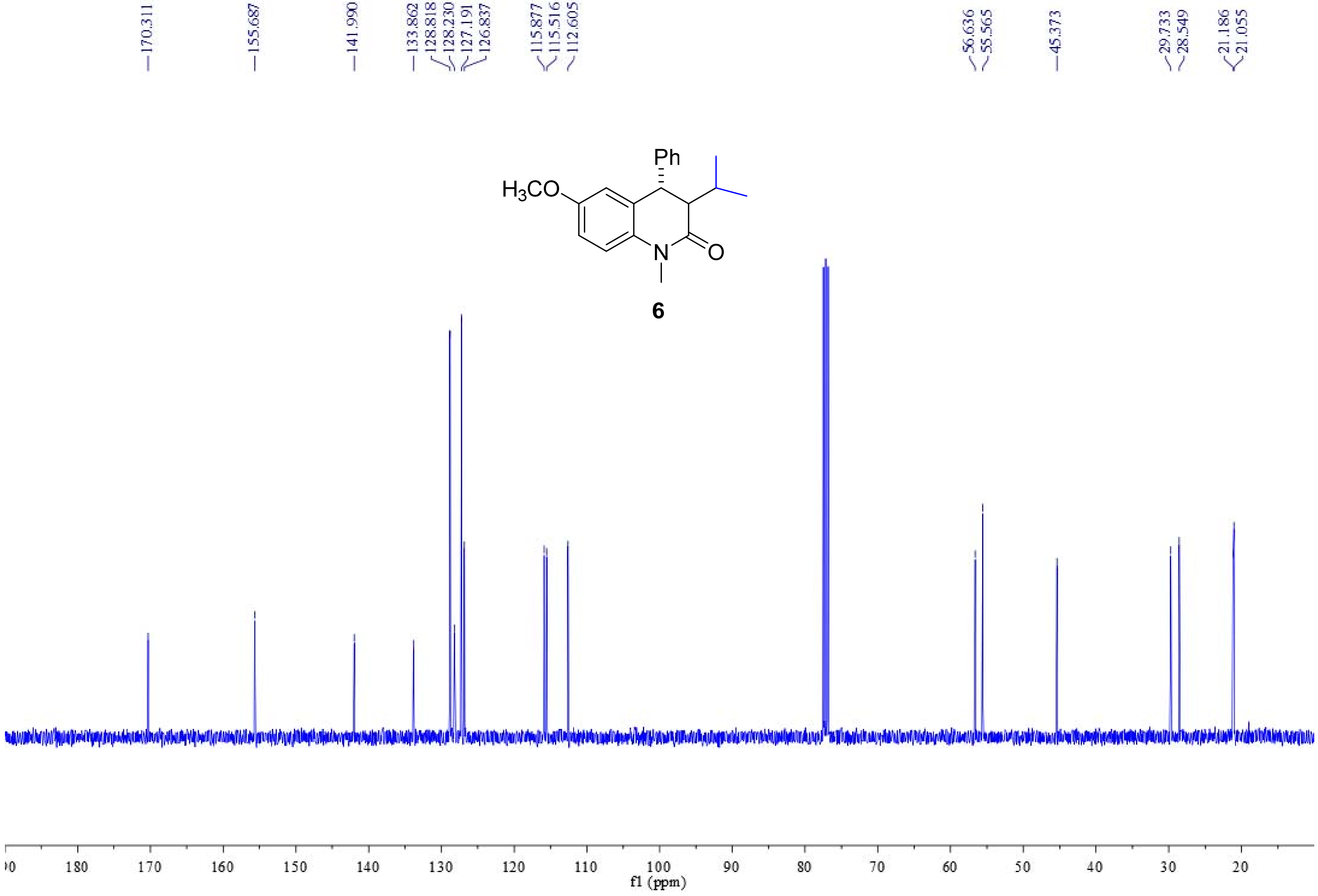
170.311
155.687
141.990
133.862
128.818
128.230
127.191
126.837
115.877
115.516
112.605
56.636
55.565
45.373
29.733
28.549
21.186
21.055
Ph
H3CO
N
O
6
f1 (ppm)

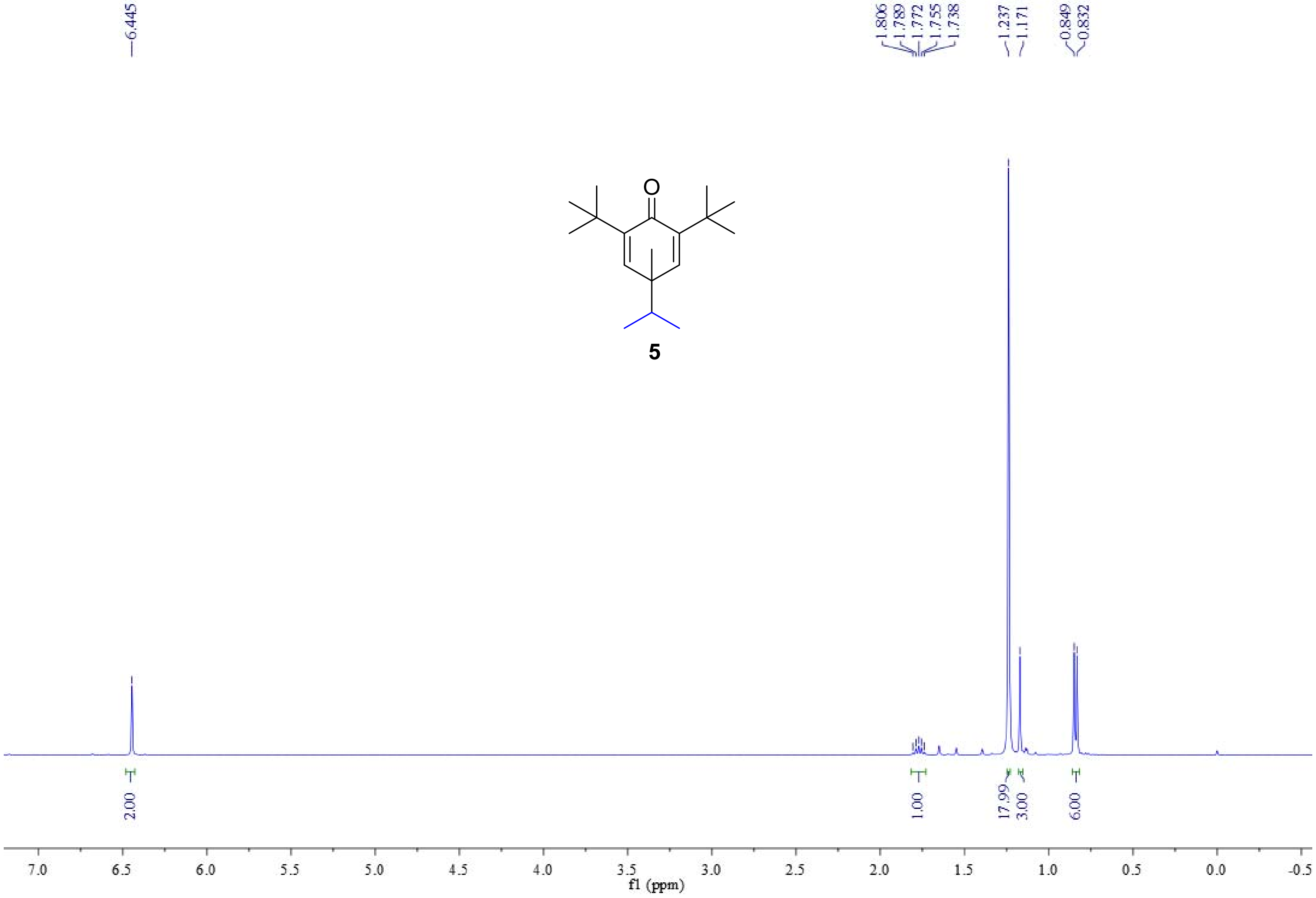

6.445
1.806
1.789
1.772
1.755
1.738
1.237
1.171
0.849
0.832
O
5
2.00
1.00
17.99
3.00
6.00
7.0
6.5
6.0
5.5
5.0
4.5
4.0
3.5
3.0
2.5
2.0
1.5
1.0
0.5
0.0
-0.5
f1 (ppm)

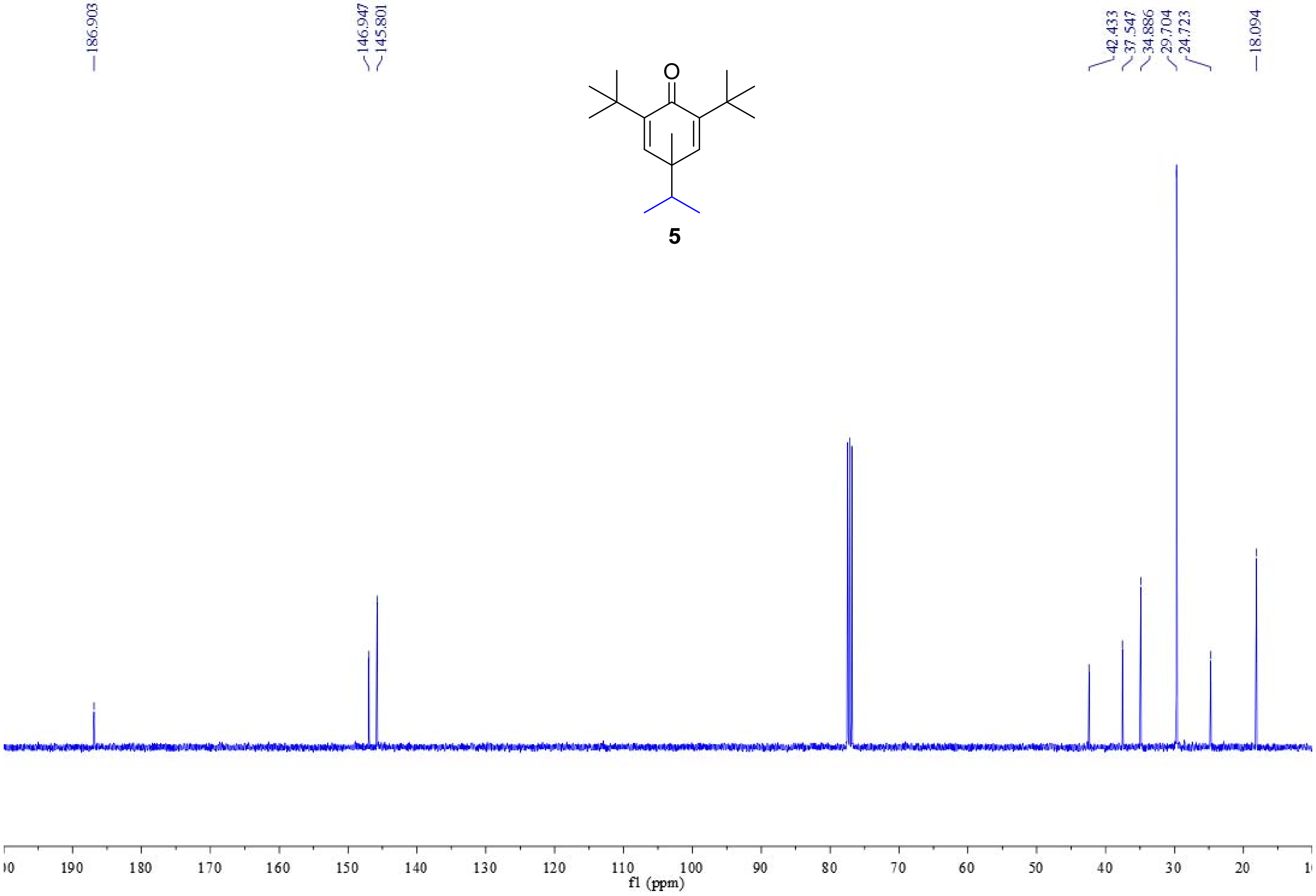
186.903
146.947
145.801
42.433
37.547
34.886
29.704
24.723
18.094
O
5
190 180 170 160 150 140 130 120 110 100 90 80 70 60 50 40 30 20
f1 (ppm)
